# Preliminary evidence for association of genetic variants in pri-miR-34b/c and abnormal miR-34c expression with attention deficit and hyperactivity disorder

**DOI:** 10.1038/tp.2016.151

**Published:** 2016-08-30

**Authors:** I Garcia-Martínez, C Sánchez-Mora, M Pagerols, V Richarte, M Corrales, C Fadeuilhe, B Cormand, M Casas, J A Ramos-Quiroga, M Ribasés

**Affiliations:** 1 Psychiatric Genetics Unit, Vall d'Hebron Research Institute, Universitat Autònoma de Barcelona, Barcelona, Spain; 2 Department of Psychiatry, Hospital Universitari Vall d'Hebron, Barcelona, Spain; 3 Biomedical Network Research Centre on Mental Health (CIBERSAM), Madrid, Spain; 4 Department of Psychiatry and Legal Medicine, Universitat Autònoma de Barcelona, Barcelona, Spain; 5 Departament de Genètica, Microbiologia i Estadística, Facultat de Biologia, Universitat de Barcelona, Barcelona, Spain; 6 Centro de Investigación Biomédica en Red de Enfermedades Raras, Madrid, Spain; 7 Institut de Biomedicina de la Universitat de Barcelona, Barcelona, Spain; 8 Institut de Recerca Pediàtrica, Hospital Sant Joan de Déu, Esplugues, Barcelona, Spain

## Abstract

Attention deficit and hyperactivity disorder (ADHD) is a prevalent neurodevelopmental disorder characterized by impairment to sustain attention and inability to control impulses and activity level. The etiology of ADHD is complex, with an estimated heritability of 70–80%. Under the hypothesis that alterations in the processing or target binding of microRNAs (miRNAs) may result in functional alterations predisposing to ADHD, we explored whether common polymorphisms potentially affecting miRNA-mediated regulation are involved in this psychiatric disorder. We performed a comprehensive association study focused on 134 miRNAs in 754 ADHD subjects and 766 controls and found association between the miR-34b/c locus and ADHD. Subsequently, we provided preliminary evidence for overexpression of the miR-34c-3p mature form in peripheral blood mononuclear cells of ADHD subjects. Next, we tested the effect on gene expression of single-nucleotide polymorphisms within the ADHD-associated region and found that rs4938923 in the promoter of the pri-miR-34b/c tags *cis* expression quantitative trait loci for both miR-34b and miR-34c and has an impact on the expression levels of 681 transcripts in *trans*, including genes previously associated with ADHD. This gene set was enriched for miR-34b/c binding sites, functional categories related to the central nervous system, such as axon guidance or neuron differentiation, and serotonin biosynthesis and signaling canonical pathways. Our results provide preliminary evidence for the contribution to ADHD of a functional variant in the pri-miR-34b/c promoter, possibly through dysregulation of the expression of mature forms of miR-34b and miR-34c and some target genes. These data highlight the importance of abnormal miRNA function as a potential epigenetic mechanism contributing to ADHD.

## Introduction

Attention deficit and hyperactivity disorder (ADHD) is a highly prevalent neuropsychiatric disorder characterized by severe impairment to sustain attention, inability to control impulses and difficulties to modulate activity levels. Epidemiological studies report a worldwide ADHD prevalence of 5–12% for children and 3–8% for adults.^1^ The etiology of ADHD is complex, with both genetic and environmental factors having key roles.^2^ Twin, family and adoption studies suggest a major component of genetic risk factors, pointing to an estimated heritability around 70–80%.^3^ The Psychiatric Genomics Consortium has recently shown direct, empirical and quantified molecular evidence for a significant genetic contribution to ADHD and estimated a single-nucleotide polymorphism (SNP)-based heritability of 0.28, which is distant to the total ADHD heritability and highlights the need for additional studies to unravel the ADHD genetic background.^4^

Although association, linkage and animal model strategies have been followed to uncover the genetic background of ADHD, consistent genetic risk factors with a major role in its susceptibility have not yet been discovered.^5^ Pathogenic models of ADHD have traditionally focused on the dysregulation of the dopaminergic, serotoninergic and noradrenergic neurotransmission systems, the regulation of neurotransmitter release (SNARE complex) and on neurotrophic factors.^6^ In addition to candidate–gene association studies, genome-wide association studies (GWAS) have also been carried out in nine independent data sets in ADHD, seven of which focused on childhood samples and two on adults.^3, 5, 7, 8^ However, none of them reported genome-wide significant findings or highlighted previous candidate genes for ADHD or identified associations overlapping with those of other GWAS.

Research on ADHD has traditionally focused on protein-coding sequences, but some data suggest that most of the common phenotypic variation would stem from variants in regulatory elements rather than in protein-coding sequences.^9^ Thus, there is a growing interest into genetic variation affecting epigenetic mechanisms involved in the dysregulation of gene expression. Among them, microRNAs (miRNAs) have emerged as strong candidates. These small non-coding single-stranded RNA molecules have important gene-regulatory roles by pairing to the messenger RNA (mRNAs) of protein-coding genes to direct their posttranscriptional repression.^10^ Some observations, however, suggest that miRNAs can also target other genomic regions, such as 5′-UTR elements, promoters or coding sequences, and additionally they may even be able to stimulate gene expression. The miRNAs configure a complex network where individually they may regulate hundreds of genes and collectively they are predicted to modulate from 30 to 80% of the human genome.^11^ Thus, slight variations affecting this system may lead to altered miRNA function, have pleiotropic effects, and contribute to part of the genetic heterogeneity underlying complex diseases. Interestingly, around 70% of all known miRNAs are expressed in the brain and have key roles in cell-fate specification and survival, neurite projection and synaptic plasticity.^12^ Moreover, miRNAs are known to be implicated in psychiatric and neurological disorders. A GWAS in schizophrenia revealed that the strongest association corresponded to miR-137, and SNPs in multiple other miRNAs, such as miR-206, miR-198, miR-24 or miR-30e, have also been associated with this disorder.^13, 14^ Abnormal miRNA biogenesis and expression levels have been observed in schizophrenia, bipolar or autism spectrum disorder (ASD). Moreover, variants within the miR-183-96-182 cluster locus or in the miR-641 binding sites at *SNAP25* gene have been associated with ADHD and impulsivity, respectively.^15, 16^ In addition, a recent study reported altered circulating levels of miRNAs in ADHD subjects and suggested that miR-107 levels below a certain threshold were highly predictive and specific for ADHD.^17^

Considering this background altogether, we attempted to unravel novel susceptibility factors for ADHD by conducting a case–control association study focused on miRNAs and their target genes. We selected miRNAs based on the previous association of their validated targets to psychiatric illness, giving priority to those linked to ADHD. Subsequently, we conducted a case–control association study in 754 adult ADHD subjects and 766 healthy controls. The impact of the associated variants on gene expression was evaluated through analysis of Quantitative Trait Loci (eQTLs) in *cis* and *trans* in the peripheral blood mononuclear cells (PBMCs) from subjects with ADHD. Top hits from the *trans*-eQTL results were considered for functional and canonical pathway overrepresentation and gene-network construction.

## Materials and methods

### Case–control association study

#### Subjects

The clinical sample consisted of 754 Caucasoid Spanish adults with ADHD (61% combined, 36% inattentive and 3% hyperactive/impulsive). Sixty-seven percent of the individuals were male and the average age at assessment was 33 years (s.d.=12.7). The evaluation of the ADHD diagnosis was carried out with the Structured Clinical Interview for DSM-IV Axis I and II Disorders (SCID-I and SCID-II) and with the Conners' Adult ADHD Diagnostic Interview for DSM-IV (CAADID parts I and II).^18^ The severity of ADHD symptoms was assessed with the long version of the Conners' ADHD Rating Scale (self-report [CAARS-S:L] and observer [CAARS-O:L]),^19^ the ADHD rating scale,^20^ the ADHD screening checklist^21^ and the Wender Utah Rating Scale for retrospective symptomatology.^22^ The level of impairment was measured with the Clinical Global Impression and the Sheehan Disability Inventory.^23, 24^ Additional tests used for clinical assessment are available in Ribasés *et al.*^25^ The control sample included 766 unrelated Caucasoid Spanish healthy individuals matched for sex with the clinical group. Sixty-seven percent of the subjects were male with an average age of 57 years (s.d.=18.4). ADHD symptomatology was excluded retrospectively under the following criteria: (1) not having been diagnosed with ADHD previously and (2) answering negatively to the life-time presence of the following ADHD symptoms: (a) often has trouble in keeping attention on tasks; (b) usually loses things needed for tasks; (c) often fidgets with hands or feet or squirms in seat and (d) often gets up from seat when remaining in seat is expected. All the subjects were evaluated and recruited at the Hospital Universitari Vall d'Hebron of Barcelona (Spain) and diagnosis was blind to genotype. The study was approved by the ethics committee of our institution and written informed consent was obtained from all the subjects before the inclusion into the study.

#### DNA isolation, SNP selection and genotyping

Genomic DNA was isolated from peripheral blood leukocytes by the salting-out procedure.

MiRNA and miRNA clusters: On the basis of literature data, 134 miRNAs distributed in 53 loci with at least one confirmed target gene involved in ADHD or other psychiatric disorders, including schizophrenia, bipolar disorder, major depression and/or ASD, were selected from a total of 5201 records of experimentally validated miRNA–target interactions available at the miRecords database (release 2012).^26^ MiRNA clusters were defined as genomic regions containing at least two contiguous miRNAs with an interdistance of <10 kb. Candidate regions spanned 5 kb upstream and 3 kb downstream from each miRNA or from the first and last miRNA of each cluster. When miRNAs were located in a transcriptional unit, some additional 5 kb upstream from the host gene were also included.

MiRNA targets: The analysis of 3′-UTR regions was restricted to target genes of those miRNAs displaying association with ADHD in the case–control association study. Experimentally validated miRNA targets were selected using information available in different databases, including TargetScan (release 6.2), Diana-TarBase (version 7.2), miRTarBase (release 2014, version 6.0), miRBase (release 21) and miRecords (release 2012), or through literature searches (Supplementary Table 1).^26, 27, 28, 29^ Considering only those mRNAs that are targeted by the miRNA found to be associated with ADHD in this study, a total of 12 candidate genes were initially selected on the basis of experimental data supporting each miRNA–mRNA interaction (*BCL2*, *CREB1*, *CRHR1*, *HMGA2*, *JAG1*, *MET*, *MYC*, *NOTCH1*, *NOTCH2*, *NOTCH3*, *NOTCH4* and *VEGFA*; Supplementary Table 1).

Tagged-SNP selection was based on genetic coverage criteria (*r*^2^⩽0.85) considering SNPs with a minor allele frequency (MAF) ⩾0.10 from the CEPH population provided by the HapMap Project Database (http://hapmap.ncbi.nlm.nih.gov/; release 28) and using the *LDSelect* Software (http://bioapp.psych.uic.edu/HapMap-LDSelect-Processor.html, University of Washington, Seattle, WA, USA). Under these conditions, we selected 214 tagSNPs covering, in terms of linkage disequilibrium (LD), genomic regions containing 134 miRNAs. We also selected 20 SNPs that tag the 3′-UTR regions of genes encoding mRNAs that are targeted by the miRNA found to be associated with ADHD in this study. From the 12 target genes initially selected, no SNPs at *NOTCH4* or *MYC* fulfilled the aforementioned criteria and, therefore, only 10 candidate genes were finally considered (*BCL2*, *CREB1*, *CRHR1*, *HMGA2*, *JAG1*, *MET*, *NOTCH1*, *NOTCH2*, *NOTCH3* and *VEGFA*). An additional variant, rs4525537, reported in the 1000 Genomes database (http://browser.1000genomes.org; release 14), was also included owing to its close location to the miR-34c target site in the *CRHR1* 3′-UTR (14 nucleotides upstream from the miRNA–mRNA binding site). From the 235 SNPs that were initially selected, a proper design was not achieved for four of them, and in some additional 10, the genotyping assay failed (genotyping rate of 97.5% for the miRNAs assay and 100% for the target genes assay). All SNPs were genotyped using the PCR-based KASP platform (Progenika Biopharma, A GRIFOLS Company, Bizkaia, Spain).

### Statistical analysis

The minimal statistical power of the sample was estimated *post hoc* using the Power Calculator for Two Stage Association Studies software (CaTS, http://csg.sph.umich.edu//abecasis/CaTS/index.html), assuming an odds ratio (OR) of 1.2, a disease prevalence of 0.05, a significance level (*α*) of 0.05, the lowest MAF observed in our control sample (0.10), and an additive model of inheritance. Potential genetic stratification in our sample was previously discarded.^30^

#### Single-marker analyses

The analysis of Hardy–Weinberg equilibrium (HWE) in the control sample (*P*_HWE_<0.01) and the comparison of genotype frequencies between cases and control subjects under a log-additive model of inheritance were performed using the SNPassoc R Library.^31^ Dominant (11+12 vs 22) and recessive (11 vs 12+22) models of inheritance were only considered for those SNPs displaying nominal association under a log-additive model. Genotype frequencies of SNPs located in chromosome X were only studied in females. All the tests were adjusted by gender. To measure and control the proportion of false positives incurred in the study, we used the false discovery rate (FDR) method, an approach to adjust for multiple comparisons that provides a good balance between discovery of statistically significant findings and controlling type I errors (*α*). The *q*-value was computed using the qvalue R package^32^ considering all the association tests performed for miRNAs and target genes jointly. We considered all the *P*-values from each SNP tested under an additive model of inheritance, as well as the *P*-values from the dominant and recessive models when considered (when the association under the additive model was significant (*P*<0.05)). Under these conditions, a total of 232 *P*-values were included and a unique FDR of 20% that corresponded to *P*<1.58e−03 was considered.

#### Gene-based analysis

Gene-based association analysis was performed using the Versatile Gene-based Association Study (VEGAS) 2 software (https://vegas2.qimrberghofer.edu.au/).^33^ We considered all genomic regions containing miRNA or target genes with more than one genotyped SNP per region. All the genotyped SNPs available from each locus were included in the analysis considering the nominal *P*-value of each association test from the single-marker analysis. Regions were defined spanning 10 kb upstream and downstream from each locus, and European samples from 1000 Genomes were used to assess their LD structure.

#### Analysis of additive and epistatic effects

To evaluate the potential additive and epistatic effects between the identified risk variants, a stepwise logistic regression procedure was implemented using the SPSS 17.0 Statistical Package (SPSS, Chicago, IL, USA), considering the overall, the combined or the inattentive samples as dependent variables and the ADHD-risk variants as independent variables. The hyperactive-impulsive sample was not considered, owing to its limited sample size.

### Gene expression assays

#### RNA isolation

Total RNA was extracted from PBMCs of a medication-naive subset of 45 ADHD individuals from the sample included in the case–control association study (68% were male and mean age was 38 years (s.d.=9.9)) and from 32 additional control subjects (69% were male and mean age was 37 years (s.d.=12.1)) using the Ficoll density gradient method and the RNeasy Midi kit (Qiagen, Hilden, Germany). RNA and miRNA quality assessment was assayed by 2100 Bioanalyzer (Agilent Technologies, Santa Clara, CA, USA).

#### Expression analysis of miR-34b and miR-34c by quantitative real-time reverse transcription in ADHD subjects and controls

In 31 out of the 45 cases mentioned above, the total RNA isolation protocol from PBMCs was performed with further enrichment of miRNAs. Considering only the miRNA found significantly associated with ADHD in this study, the expression levels of the mature forms miR-34b-3p, miR-34b-5p, miR-34c-3p and miR-34c-5p were assessed in these 31 cases and in 32 additional control subjects by reverse transcription reaction using the Taqman MicroRNA Reverse Transcription Kit (Applied Biosystems; Foster City, CA, USA). Quantitative real-time reverse transcription PCR (qRT-PCR) for each assay was run in triplicate using Taqman MicroRNA Assays (Applied Biosystems) and measured in Applied Biosystems 7900HT fast real-time PCR system. The threshold cycle (*C*_T_) was defined as the fractional cycle number at which the fluorescence exceeded the threshold of 0.2. The relative quantification of miRNA expression was calculated by the 2^−ΔΔ^^CT^ method,^34^ considering U6 as endogenous control gene after checking its stability and linearity across all the samples. Batch, gender and age were considered as covariates when a significant association with the study outcome was observed (*P*>0.20) and were kept in the statistical model if the corresponding equation parameter was significant in the fitted model (*P*<0.05). Logistic regression models were applied to compare expression differences between ADHD cases and controls. All the statistical tests were two-sided and the significance threshold was set at *P*<0.05.

#### *Cis*-eQTLs in ADHD subjects by qRT-PCR

The *cis*-eQTL analyses were assessed in the subset of 31 ADHD subjects from which genotype data for rs4938723 and rs28690953 and expression levels of miR-34b-3p, miR-34b-5p, miR-34c-3p and miR-34c-5p were available. Linear regression models were used for eQTL mapping with the SPSS 17.0 Statistical Package (SPSS). All the statistical tests were two-sided and the significance threshold was set at *P*<0.05.

#### *Trans*-eQTLs in ADHD subjects by microarray assay

Genome-wide gene expression data from microarray assays was used for the *trans*-eQTL analyses in 45 ADHD subjects (60% overlap with the ADHD sample included in the qRT-PCR assays). RNA was reverse transcribed using the Ambion WT Expression Kit (Life Technologies, Carlsbad, CA, USA). The cRNA was subsequently fragmented, labeled and hybridized with the GeneChip WT Terminal Labeling and Hybridization Kit (Affymetrix, Santa Clara, CA, USA). The samples were hybridized to the Genechip Human Gene 1.1 ST 96-Array plate (Affymetrix), covering a total of 36 079 transcripts that correspond to 21 014 genes. The array processing and data generation were assessed using the Gene Titan Affymetrix microarray platform. The raw data were pre-processed with the Robust Multichip Analysis method (including the background correction, normalization and summarization of probes values) using the R environment and the Oligo library.^35^ Transcript probes that did not correspond to known genes or matched with more than one gene in the GRCh37/hg19 human genome build (release 32) were discarded, and the study was finally restricted to 19 263 probes corresponding to 18 475 unique genes. *Trans*-eQTL analyses considering rs4938723 were performed with the R environment and the MatrixEQTL Package,^36^ including gender and batch as covariates. FDR correction was applied for multiple comparisons and statistical significance was set at *P*<0.05.

#### Functional- and pathway-enrichment analyses

MiRNA binding-site enrichment was explored using the WEB-based GEne SeT AnaLysis (WebGestalt) Toolkit (http://bioinfo.vanderbilt.edu/webgestalt/).^37^ Functional and cluster overrepresentation analyses were performed considering Biological Process category from Gene Ontology using the Database for Annotation, Visualization and Integrated Discovery resource (http://david.abcc.ncifcrf.gov/).^38^ Evaluation of overall enrichment in direct and indirect interactions between encoded proteins from our gene set was addressed with Disease Association Protein-Protein Link Evaluator software (http://www.broadinstitute.org/mpg/dapple/dapple.php).^39^ Enrichment analysis of canonical pathways and gene networks were performed using the Ingenuity Pathway Analysis software (Ingenuity Systems, Redwood City, CA, USA; http://www.ingenuity.com). Gene networks were considered of true relevance when the network score (*P*-score=−log_10_(*P*-value)) was over 3 (*P*=10^−3^) and the total number of focus molecules was above 20 out of 35. Benjamini–Hochberg's correction was used to adjust for multiple comparisons and significance threshold was set at *P*<0.05.

## Results

### Case–control association study between ADHD and genomic regions containing miRNAs or target genes

Assuming an OR of 1.2, the ADHD sample showed minimum statistical powers of 36%, 27% and 20% in the overall, combined and inattentive ADHD samples, respectively.

#### Single-marker and gene-based analyses

MiRNA and miRNA clusters: We studied 214 tagSNPs in 53 genomic regions spanning 143 miRNAs chosen on the basis of convergent evidence of the involvement of their target genes in ADHD or other psychiatric disorders in a total sample of 754 adults with ADHD and 766 controls. From the 203 successfully genotyped SNPs, eight were monomorphic (MAF<0.10) and three had significant departure from HWE in the control sample (*P*_HWE_<0.01; Supplementary Table 2). After filtering for low call rate (subjects with <90% of successfully genotyped SNPs) or gender discrepancy, a total of 23 individuals were discarded and, therefore, 192 SNPs with an average genotype call rate of 97.5% were finally considered in 748 cases and 749 controls.

When the whole ADHD sample was considered, nominal associations were found for eight SNPs located at five loci: the miR-128-2 locus and the clusters let-7a-1/let-7 f-1/let-7d, let-7a-3/miR-4763/let-7b, miR-34b/c and miR-371/372/373 (Table 1a). None of them was located in the mature or precursor forms of the miRNAs and only rs28690953 within the miR-34b/c locus, 186 bp downstream of *MIR34C* gene, remained associated with ADHD after the FDR correction (*P*=8.8e−04 under a dominant model; Supplementary Figure 1). The gene-based analysis supported the association between miR-34b/c and let-7a-1/let-7f-1/let-7d clusters and ADHD (*P*=2.8e−03 and *P*=2.9e−03, respectively; Supplementary Table 3a).

MiRNA targets: We tested the association between ADHD and tagSNPs covering 3′-UTRs of 10 experimentally validated target genes for miR-34b and/or miR-34c (*BCL2, CREB1, CRHR1, HMGA2, JAG1, MET, NOTCH1, NOTCH2, NOTCH3* and *VEGFA*). Of the 21 SNPs initially selected, three did not pass through the design pipeline, one was monomorphic (MAF<0.10) and another had significant departure from HWE (*P*_HWE_<0.01; Supplementary Table 4). After filtering subjects with low call rate (<90%), a total of 24 individuals were discarded and, therefore, 16 SNPs with an average genotype call rate of 97.5% were finally considered in 751 ADHD cases and 745 controls. The single-marker analysis showed nominal association between ADHD and four SNPs in three genes: rs1621 and rs6566 within the *MET* gene (*P*=8.3e−03, OR=1.23 (1.06−1.44) and *P*=0.017, OR=1.19 (1.03−1.39), respectively), rs699779 in the *NOTCH2* gene (*P*=7.7e−03; OR=3.45 (1.28−9.09)) and rs1175982 in the *HMGA2* gene (*P*=7.5e−03; OR=1.34 (1.08−1.67); Table 1b, Supplementary Figure 1). None of them withstood FDR correction for multiple comparisons. The gene-based analysis supported the association between ADHD and the *MET* and *HMGA2* genes (*P*=5.6e−03 and *P*=7.1e−03, respectively; Supplementary Table 3b). *NOTCH2* was not considered as it contained only one marker.

#### Analysis of additive and epistatic effects

As ADHD was associated with SNPs in the miR-34b/c cluster and in the 3′-UTRs of three validated target genes (*MET*, *NOTCH2* and *HMGA2*), we further tested potential additive and epistatic effects among them. The combined effect of all ADHD risk variants was estimated to account for 3.2 and 4.5% of the variance of the overall (*P*=1.2e−06) and combined ADHD (*P*=3.4e−07), respectively, which resulted in sensibility and specificity values over 55%. No significant interactions between miR-34b/c and any of the targets genes were detected.

### Gene expression assays

#### Expression analysis of miR-34b and miR-34c by qRT-PCR in ADHD subjects and controls

Expression levels of the mature forms of miR-34b and miR-34c were evaluated by qRT-PCR in PBMCs from 31 medication-naive ADHD individuals and 32 controls. Significant overexpression of miR-34c-3p was identified in ADHD subjects (*P*=6.5e−03; Exp(B)=4.19; CI=1.25–14.05), with 1.4-fold higher levels in patients as compared with controls. MiR-34b-3p also showed a trend towards increased expression in ADHD when compared with controls (*P*=0.058; Exp(B)=2,70; CI=0.89–8.20) and no differences were observed for miR-34b-5p or miR-34c-5p.

#### Identification of *cis*-eQTLs in ADHD subjects by qRT-PCR

**Figure 1:**
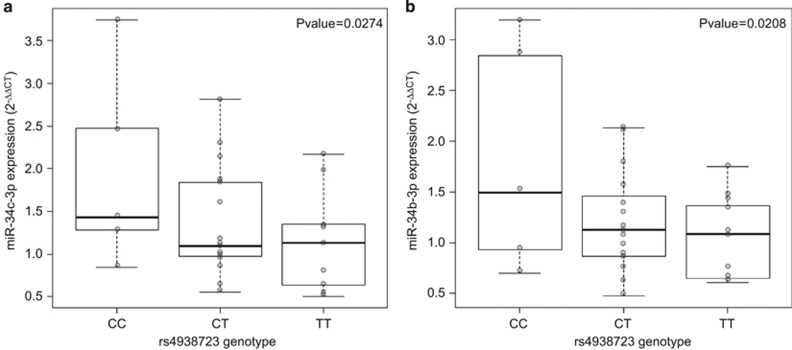
Graphical representation of significant *cis*-eQTL analysis between rs4938723 and 2^−ΔΔCT^ expression values of (**a**) miR-34b-3p and (**b**) miR-34c-3p. eQTL, expression quantitative trait locus.

To determine whether expression differences in miR-34b and miR-34c resulted from allele-specific differences in the identified ADHD-associated risk locus, we searched for *cis*-eQTLs in PBMCs from a subset of 31 ADHD subjects from whom miR-34b/c genotypes and qRT-PCR expression data were available. Interestingly, a significant inverse correlation between the dosage of the rs4938723T allele in the promoter region of the primary transcript of miR-34b/c (pri-miR-34b/c) and expression levels of miR-34b-3p and miR-34c-3p was observed (*P*=0.021, β=−0.41 and *P*=0.027, β=−0.44, respectively; Figure 1). No differences were detected for miR-34b-5p and miR-34c-5p or when rs28690953 was considered.

#### Identification of *trans*-eQTLs in ADHD subjects by microarray assay

Next, we performed a transcriptome-wide *trans-*eQTL analysis considering the dosage of the *cis*-associated rs4938723T allele and microarray expression data from PBMCs of 45 ADHD subjects, which revealed 681 differentially expressed transcripts, 292 of which were upregulated and 389 downregulated (*P*<0.05; Supplementary Table 5). Among them, we identified genes previously associated with ADHD in candidate–gene association studies and GWAS, such as *SYT2*, *HTR2C*, *SHFM1, DCLK1, TMX3, BDNF, VAMP2 or TPH2* (Supplementary Table 6).

#### Functional- and pathway-enrichment analyses

**Figure 2:**
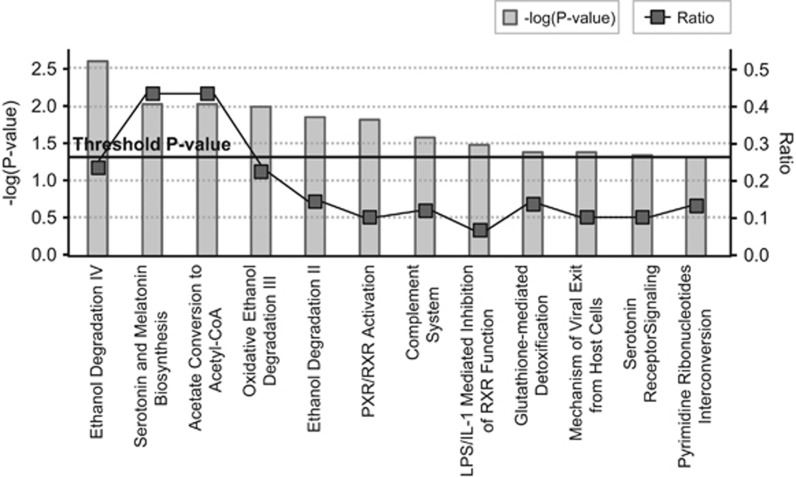
Significantly enriched canonical pathways considering differentially expressed genes identified in the trans-eQTL analysis. Along the *x* axis of the bar chart, the canonical pathways are shown. Along the left *y* axis, the statistical significance is indicated as −log(*P*-value), calculated using the right-tailed Fisher's exact test. Gray bars denote statistical significance of the enrichment for each canonical pathway. The black straight line stands for the threshold above which there is significant enrichment (by default *P*-value <0.05). Along the right *y* axis, the ratio parameter is shown, calculated by the numbers of genes in a given pathway that meet cutoff criteria, divided by total numbers of genes that make up that pathway. Black squares express the ratio for each pathway. eQTL, expression quantitative trait locus.

**Figure 3:**
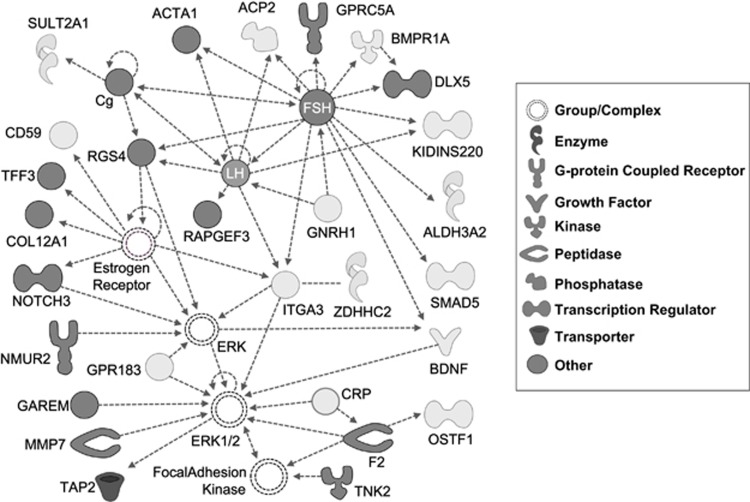
Ingenuity pathway best network (score=37, focus molecules=28) based on the top differentially expressed genes from the *trans*-eQTL analysis considering the rs4938723T risk variant in the pri-miR-34b/c promoter. Mapped molecules from the data set are represented as nodes with colored background and interacting proteins added from the Ingenuity database correspond to clear background nodes. Molecule over- and under-expression are denoted by light and dark gray coloring, respectively. eQTL, expression quantitative trait locus.

Overrepresentation of miR-34b binding sites (*P*=1.64e−03; Supplementary Table 7) as well as enrichment for indirect protein–protein interactions (seed indirect degrees mean=55.11, *P*=0.042) were identified considering the 681 differentially expressed transcripts from the *trans-*eQTL analyses (data not shown). Functional enrichment analyses revealed 36 categories nominally associated with our gene set, mainly related to lipid metabolism, and neuron dynamics and axonogenesis (Supplementary Table 8). Accordingly, the functional clustering showed two significantly enriched groups related to neuron development and differentiation, and axonogenesis (score=1.6), and lipid biosynthesis and metabolism (score=1.5; Supplementary Table 9). We also identified enrichment for categories related to psychiatric disorders such as ‘susceptibility to attention deficit/hyperactivity disorder' (*P*=0.032), ‘refractory schizophrenia' (*P*=0.032), major affective disorder (*P*=0.032), ‘susceptibility to anorexia nervosa type II' (*P*=0.032) or ‘susceptibility to bulimia nervosa type II' (*P*=0.032), as well as for neurologic diseases such as ‘Huntington disease grade II' (*P*=0.032; data not shown). In addition, 12 canonical pathways were overrepresented in the gene set, among which we highlight ‘Serotonin and Melatonin Biosynthesis' (*P*= 9.33e−03) and ‘Serotonin Receptor Signaling' (*P*=0.046; Figure 2). Interesting biological functions were also found enriched, such as ‘synthesis of neurotransmitter' (*P*=0.013), ‘release of neurotransmitter' (*P*=8.43e−03), ‘influx of dopamine' (*P*=0.032), ‘release of GABA' (*P*=0.032), ‘synthesis of 5-hydroxytryptamine' (*P*=9.32e−03), ‘recycling of synaptic vesicles' (*P*=0.032) or ‘declarative memory' (*P*=0.032; data not shown). We also identified 20 significant networks, three of which had more than 20 focus molecules. The best generated network was highly scored (score=37; focus molecules=28), had follicle stimulating hormone as a central node and displayed 312 specific overrepresented functions and diseases (Figure 3, Supplementary Table 10).

## Discussion

Under the hypothesis that alterations affecting the expression, processing or target binding of miRNAs may result in functional alterations predisposing to ADHD, we explored whether common SNPs potentially affecting miRNA-mediated regulation are involved in this psychiatric disorder.

We found association between a sequence variant within the miR-34b/c locus and ADHD and provided preliminary evidence for overexpression of the miR-34c-3p mature form in PBMCs of ADHD subjects. We also assessed whether SNPs in the ADHD-associated locus that contains miR-34b/c were involved in the disorder through an effect on miRNA expression and found that rs4938723, located in the promoter region of the pri-miR-34b/c, tags *cis*-eQTLs for miR-34b-3p and miR-34c-3p. The rs4938723T allele dosage was associated with decreased expression levels of the mature forms of miR-34b and miR-34c in PBMCs of ADHD subjects, which is in concordance with previous studies showing correlation between the rs4938723C allele and a significant increase in luciferase activity.^40^ Interestingly, this sequence variant is located within a CpG island, 423 bp upstream from the transcription start site of this cluster of miRNAs, and may affect a predicted GATA-binding site.^40^

Recent investigations link miRNAs to psychiatric disorders including ADHD, schizophrenia, ASD, bipolar disorder and major depressive disorder.^14, 16, 41^ Specifically, although it has crucial roles in tumorigenesis, the miR-34 family participates in neuronal development, stem cell differentiation, ageing, spermatogenesis, as well as in metabolic and cardiovascular functions, and has been implicated in noncancerous diseases such as brain disorders, osteoporosis or cardiovascular complications.^42^ MiR-34b and miR-34c have been found downregulated in the amygdala, substantia nigra and frontal cortex in Parkinson's disease and have been linked to Alzheimer, anxiety or preclinical manifestation of Huntington's disease.^43, 44, 45, 46^ Regarding psychiatric illness, miR-34a dysregulation has been associated with bipolar disorder, schizophrenia and major depressive disorder and a recent study reported aberrant expression of miR-34b and miR-34c in the peripheral blood of subjects with ASD.^47, 48, 49, 50^

In addition to genetic variants in miRNAs, we focused our case–control association study on several validated targets of miR-34b/c and found significant associations of ADHD with *MET, HMGA2* and *NOTCH2* genes. Although not being previously related to ADHD, *MET* and *NOTCH2* have emerged as candidate genes for ASD.^51, 52^ The *MET* gene might also be involved in schizophrenia, is highly expressed in the developing brain and has been implicated in GABAergic neuronal development, cortical thickness and connectivity in temporal–parietal regions affecting the Default Mode Network.^51, 53, 54, 55^ The *MET* gene is known to mediate the association between air pollution and cognitive development, and independent studies have reported gene–environment interactions between *MET* gene variants and exposure to air pollutants in ASD.^56^ Importantly, as ADHD and ASD often co-occur and share neuropsychological features, neurobiological substrates and increased risk after exposure to air pollutants, further studies are warranted to disentangle the role of *MET* gene in the overlapping genetic background between these disorders.^4, 57^

The transcriptome-wide *trans*-eQTL analysis considering the rs4938723 miR-34b/c polymorphism highlighted 681 transcripts, including genes previously implicated in ADHD. This gene set was mainly related to neurotransmission, neuron development and differentiation, axonal growth and guidance, cellular morphology and projection, as well as to lipid biosynthesis and metabolism, which is of particular importance in central nervous system (CNS) injuries and psychiatric disorders including ADHD.^58, 59^ Among the top three genes, we found Synaptotagmin 2 (*SYT2*), an essential component of the calcium-triggering machinery for neurotransmitter release that was found associated with ADHD.^60^ As expected, among the *trans*-eQTLs, we also found experimentally validated miR-34b/c target genes, such as *NOTCH3,* enrichment for miR-34b/c binding sites, including predicted targets in ADHD-associated genes such as *HTR2C* and *VAMP2*, and genes related to different psychiatric disorders including schizophrenia, major affective disorder or eating disorders.^5^ Taken together, these data reinforce a possible scheme for miR-34b/c having a role in adult ADHD etiopathogenesis through a multifactorial mechanism integrating miRNAs as well as genes previously associated with ADHD and/or other psychiatric conditions.

There are several methodological considerations, however, that should be discussed. (1) The results of the present association study should be considered preliminary until further replication in additional cohorts. In this regard, we explored results of the ADHD GWAS meta-analysis from the Psychiatric Genomics Consortium and observed no significant association between rs28690953 or rs4938723 and ADHD (*P*=0.56 and *P*=0.33, respectively). Heterogeneity between populations, methodologies and statistical tests may explain discordant results across studies, making it difficult to reproduce the same findings and to establish direct comparisons between reports. Our study included only adult ADHD patients from Spain, whereas the Psychiatric Genomics Consortium sample consists mainly of children and adolescent Caucasians of European ancestry. These age differences as well as differential proportion of remitting and persisting ADHD subjects between samples may account for inconsistencies. Other explanations could also contribute to these discordant results, such as sample size, comorbid disorders as well as the proportions of clinical subtypes or genders. In addition, our findings regarding miR-34b and mirR-34c expression levels also require replication in a larger and independent cohort as the limited sample size considered may have led to imprecise estimates of the magnitude of the miRNA expression differences and could have prevented us from detecting significant differences for miR-34b, which only displayed a trend towards significance. (2) The modest sample size of the association study may have prevented us from detecting susceptibility loci with very small effects in ADHD. In addition, although the study was designed to assure a full genetic coverage in terms of LD, the minimum MAF threshold was set at 0.10, which may also have led to an underestimation of the contribution of less common sequence variants. (3) Our inability to detect, in the single-marker analysis, the association between ADHD and rs4938723 that tags eQTLs for miR-34b and miR-34c, may be attributed to the complex genetic background of ADHD as well as potential gene-by-environment interactions, which might be particularly relevant in ADHD. (4) As one of the tagSNPs in the miR-34b/c locus could not be tested owing to experimental constraints, it is still possible that additional sequence variants in the region may contribute to the dysregulation of miR-34b and/or miR-34c. (5) The selection of the FDR approach as the multiple-testing correction method rather than other more conservative strategies, such as Bonferroni, was based on the fact that the latter may be too stringent to identify subtle genetic factors involved in the etiology of a complex disease. In addition, the Bonferroni correction requires independence between all performed tests and, therefore, may be too restrictive in our study as some of the selected SNPs show some degree of LD (LD threshold: *r*^2^=0.85), and the different inheritance models tested for some of the SNPs are not fully independent. (6) Although SNPs in 3′-UTR regions that are targeted by miR-34b or miR-34c were selected solely on the basis of genetic coverage criteria, prediction of the functional significance of the ADHD risk variants in *MET*, *NOTCH2* and *HMGA2* genes supports their impact on miRNA target site recognition but none of them involved miR-34. (7) As we limited the gene expression analyses to miRNAs and SNPs showing significant evidence for association with ADHD, we cannot rule out the contribution to ADHD of other variants affecting the processing or target binding of miRNAs. (8) All the participants were recruited by a single clinical group in a restricted geographic area (Barcelona), and the diagnosis of ADHD was based on structured interviews with no variability in measurements across data. In addition, expression patterns were assessed in medication-naive subjects with no comorbid disorders, which may facilitate the identification of ADHD-related transcriptional signatures that might be neglected by a broader recruitment strategy. (9) The expression experiments were performed in PBMCs and, therefore, further evidence in brain tissues is required to disentangle the involvement of miR-34b and miR-34c in ADHD. It is worth pointing out, however, that the expression profiling of miRNAs shows correlation between brain and PBMCs, where both tissues cluster together based on the pattern of miRNA expression.^61^ A recent meta-analysis also showed that *cis*-eQTLs in the peripheral blood and brain display strong overlap and that there is solid evidence supporting PBMCs as a proxy to study transcriptional and epigenetic biosignature in different psychiatric disorders.^62^ Consistently, at the transcriptome level, whole blood also shares significant gene expression similarities with multiple CNS tissues, suggesting that the use of peripheral gene expression may be a useful surrogate for gene expression in the CNS.^63^ Furthermore, similarities between the mechanisms of transduction and receptor expression in cells of the CNS and blood lymphocytes have also been described.^64^ In this sense, disturbances in cellular functions and metabolism in the CNS, as well as alterations in the major neurotransmitter and hormonal systems, have been identified in neuropsychiatric disorders concomitant with altered functioning and metabolism of lymphocytes, which also support that the latter could reflect the metabolism in brain cells.^64, 65^

In conclusion, to our knowledge, this is the first comprehensive attempt to evaluate whether miRNAs are involved in ADHD and we identified sequence variants in the pri-miR-34b/c associated with ADHD and with expression levels of the mature forms of miR-34b and miR-34c in ADHD subjects. This work increases the understanding of biological mechanisms by which epigenetic functions may contribute to ADHD and highlights the importance of miRNAs in the etiology of the disorder.

## Figures and Tables

**Table 1:** Single-marker association study in 754 adult ADHD patients and 766 sex-matched unrelated controls considering SNPs in (a) microRNA genes and (b) 3′-UTR of experimentally validated target genes

	*SNP (risk allele)*	Genotypes*												
		*Controls* N *(%)*			*Cases* N *(%)*				*Log-additive (22 vs 12 vs 11)*		*Genotypes 11+12 vs 22*		*Genotypes 11 vs 12+22*	
		*11*	*12*	*22*	*11*	*12*	*22*	P*-value*	*OR (95% CI)*	P*-value*	*OR (95% CI)*	P*-value*	*OR (95% CI)*	P*-value*
*(a) miRNA*														
* hsa-miR-128-2*	rs9311107 (T)	205 (27.4)	388 (51.9)	155 (20.7)	245 (32.9)	365 (48.9)	136 (18.2)	0.064	1.18 (1.02–1.40)	0.028	1.18 (0.91–1.52)	0.224	1.30 (1.04–1.61)	0.022
	rs6801590 (A)	367 (49.2)	312 (41.8)	67 (9.0)	402 (53.8)	295 (39.5)	50 (6.7)	0.103	1.19 (1.01–1.39)	0.035	1.36 (0.94–2.00)	0.099	1.20 (0.98–1.47)	0.074
														
* hsa-let-7a-1/hsa-let-7f-1/hsa-let-7d*	rs7865876 (G)	320 (43.0)	352 (47.2)	73 (9.8)	371 (50.3)	308 (41.7)	59 (8.0)	0.017	1.25 (1.06–1.47)	6.2e−03	1.25 (0.87–1.79)	0.222	1.35 (1.10–1.64)	4.7e−03
	rs8115 (G)	9 (1.2)	148 (19.9)	585 (78.9)	9 (1.2)	184 (24.7)	553 (74.1)	0.091	1.25 (1.00–1.56)	0.048	1.30 (1.02–1.65)	0.032	0.99 (0.39–2.52)	0.991
														
* hsa-let-7a-3/hsa-mir-4763/hsa-let-7b*	rs9616084 (C)	432 (58.0)	275 (37.0)	37 (5.0)	476 (64.0)	236 (31.7)	32 (4.3)	0.065	1.23 (1.02–1.45)	0.030	1.62 (0.72–1.89)	0.538	1.28 (1.04–1.59)	0.019
	rs6520050 (A)	309 (41.5)	346 (46.5)	89 (12.0)	339 (45.6)	337 (45.4)	67 (9.0)	0.099	1.18 (1.01–1.37)	0.039	1.36 (0.98–1.92)	0.064	1.18 (1.22–1.45)	0.111
														
* hsa-miR-34b/hsa-miR-34c*	rs28690953 (G)	18 (2.4)	178 (23.8)	553 (73.8)	23 (3.1)	231 (31.0)	492 (65.9)	3.9e−03	1.37 (1.12–1.66)	1.6e−03**	1.46 (1.17–1.82)	8.9e−04**	1.29 (0.69–2.41)	0.420
														
* hsa-miR-371/hsa-miR-372/hsa-miR-373*	rs8103186 (C)	33 (4.4)	256 (34.3)	458 (61.3)	46 (6.2)	279 (37.3)	422 (56.5)	0.099	1.20 (1.01–1.43)	0.033	1.22 (0.99–1.50)	0.058	1.42 (0.90–2.25)	0.132

*(b) Gene*														
* MET*	rs1621 (G)	65 (8.8)	345 (46.5)	332 (44.7)	93 (12.4)	35 (48.1)	295 (39.5)	0.024	1.23 (1.06–1.44)	8.3e–03	1.24 (1.01–1.52)	0.040	1.48 (1.06–2.07)	0.021
	rs6566 (G)	212 (28.8)	367 (49.9)	157 (21.3)	245 (32.8)	377 (50.5)	125 (16.7)	0.048	1.19 (1.03–1.39)	0.017	1.35 (1.04–1.75)	0.024	1.20 (0.97–1.39)	0.096
														
* NOTCH2*	rs699779 (T)	561 (75.6)	164 (22.1)	17 (2.3)	596 (79.8)	146 (19.5)	5 (0.7)	0.011	1.31 (1.05–1.64)	0.015	3.45 (1.28–9.09)	7.7e−03	1.27 (1.00–1.64)	0.052
														
* HMGA2*	rs11175982 (T)	31 (4.2)	192 (26.0)	516 (69.8)	31 (4.1)	244 (32.6)	474 (63.3)	0.019	1.23 (1.02–1.47)	0.026	1.34 (1.08–1.67)	7.5e−03	0.99 (0.59–1.64)	0.957

Abbreviations: ADHD, attention deficit hyperactivity disorder; CI, confidence interval; miRNA, microRNA; OR, odds ratio; SNP, single-nucleotide polymorphism; UTR, untranslated region.

*Allele 1 denotes always risk allele and all calculations are performed considering this allele.

**Statistically significant *P*-values after applying a false discovery rate of 20% (*P*-value <1.58e−03 for the association tests with SNPs both in the miRNA genes and in the selected target genes).
