# Supplementary material for: Preliminary evidence for association of genetic variants in pri-miR-34b/c and abnormal miR-34c expression with attention deficit and hyperactivity disorder
Source: Transl Psychiatry. 2016 Aug 30;6(8):e879–. doi: 10.1038/tp.2016.151 (PMC5022091; doi:10.1038/tp.2016.151)
Supplement: Supplementary Legends [file tp2016151x1.doc]

**SUPPLEMENTARY INFORMATION**

Contents and Summary

**Supplementary Tables**

- **Supplementary Table 1**: MiR-34b and/or miR-34c target genes considered in the case-control association study.
- **Supplementary Table 2:** Description of SNPs in 134 miRNA gene regions that are candidate for ADHD.
- **Supplementary Table 3:** Nominal associations identified in the gene-based association analysis using VEGAS software and considering regions encoding (**a**) miRNA or (**b**) miRNA target genes containing more than one genotyped SNP per region.
- **Supplementary Table 4:** Description of SNPs in 3’UTRs of target genes for miR-34b/c.
- **Supplementary Table 5:** Results from the *trans*-eQTL analyses using the MatrixEQTL R Package (P-value<0.05).
- **Supplementary Table 6:** Genes that have previously been associated with ADHD and with other psychiatric disorders by either (a) candidate-gene association studies (CGAS) and/or (b) genome-wide association studies (GWAS) (coincidence with a GWAS top finding or close vicinity).
- **Supplementary Table 7:** MiRNA binding site enrichment analyses.
- **Supplementary Table 8:** Results from the GO term enrichment analyses using the DAVID tool.
- **Supplementary Table 9:** Significant GO terms and clusters considering the gene set identified in the *trans*-eQTL analysis in 45 adults with ADHD (681 transcripts; enrichment score>2 and P-value<0.05).
- **Supplementary Table 10:** Significant Ingenuity Pathway networks (score>3) and top associated functions and diseases.

**Supplementary Images**

- **Supplementary Figure 1:** Graphical representation of the chromosomal region containing the miR-34b/c cluster and the 3’ Untranslated Regions (3’UTRs) of the *MET*, *NOTCH2* and *HMGA2* genes, including the SNPs selected for the association study and the putative miRNA binding sites. (*) In bold, SNPs associated with ADHD.
