# Supplementary material for: Preliminary evidence for association of genetic variants in pri-miR-34b/c and abnormal miR-34c expression with attention deficit and hyperactivity disorder
Source: Transl Psychiatry. 2016 Aug 30;6(8):e879–. doi: 10.1038/tp.2016.151 (PMC5022091; doi:10.1038/tp.2016.151)
Supplement: Supplementary Table 1 [file tp2016151x2.doc]

**Supplementary Table 1** MiR-34b and/or miR-34c target genes considered in the case-control association study.

| **Gene** | **Refseq** | **miRNA mature ID** | **Bibliography supporting the interaction (PMID)** | **Interaction supported in miRecords** | **Interaction supported in Tarbase** | **Interaction supported in miRTarBase** | **Interaction supported in TargetScan** | **Psychiatric disorders associated with the target gene** |
| --- | --- | --- | --- | --- | --- | --- | --- | --- |
| VEGFA | NM_003376 | hsa-miR-34b | 18320040 | Yes | Yes | Yes | Yes | Schizophrenia^1, 2^  Bipolar Disorder^3^  Major depression^3, 4^ |
| NOTCH3 | NM_000435 | hsa-miR-34c | 18803879 | Yes | No | No | No | - |
| NOTCH2 | NM_024408 | hsa-miR-34b | 18803879 | Yes | No | Yes | Yes | Autism^5^ |
|  |  | hsa-miR-34c | 18803879, 22498974 | Yes | No | No | Yes |  |
| NOTCH4 | NM_004557 | hsa-miR-34b | 18803879 | Yes | No | Yes | No | Schizophrenia^6, 7^  Bipolar Disorder^8^ |
|  |  | hsa-miR-34c | 18803879, 22074923 | Yes | No | Yes | Yes |  |
| HMGA2 | NM_003484 | hsa-miR-34b | 18803879, 22564666 | Yes | No | Yes | Yes | - |
|  |  | hsa-miR-34c | 18803879 | Yes | No | No | No |  |
| NOTCH1 | NM_017617 | hsa-miR-34b | 18803879, 22498974 | Yes | No | Yes | Yes | Schizophrenia^9^  Major Depression^10^ |
|  |  | hsa-miR-34c | 18803879, 22498974 | Yes | No | Yes | Yes |  |
| BCL2 | NM_000657 | hsa-miR-34b | 18803879 | Yes | Yes | Yes | Yes | Schizophrenia^11^  Bipolar Disorder^12, 13^  Autism^14^  Anxiety^15^ |
|  |  | hsa-miR-34c | 18803879 | Yes | Yes | Yes | Yes |  |
| MYC | NM_002467 | hsa-miR-34b | 18802929, 20212154 | Yes | Yes | Yes | Yes | - |
|  |  | hsa-miR-34c | 20212154, 26250586 | Yes | Yes | Yes | No |  |
| CREB1 | NM_134442 | hsa-miR-34b | 19258499 | Yes | Yes | Yes | Yes | Schizophrenia^16, 17^  Bipolar Disorder^17^  Major Depression^18^ |
|  |  | hsa-miR-34c | No | No | No | No | Yes |  |
| MET | NM_001127500 | hsa-miR-34b | 22419847 | No | Yes | Yes | Yes | Schizophrenia^19^  Autism^20, 21^ |
|  |  | hsa-miR-34c | 22419847, 25611392, 23922103 | No | Yes | Yes | Yes |  |
| JAG1 | NM_000214 | hsa-miR-34b | 22113133 | No | No | Yes | Yes | Schizophrenia^22^ |
|  |  | hsa-miR-34c | 22498974 | No | No | No | Yes |  |
| CRHR1 | NM_001145146 | hsa-miR-34c | 25123309, 21976504 | No | No | No | Yes | Bipolar Disorder^23^  Major Depression^6, 24^  Anxiety^25^ |

**Supplementary Bibliography**

1. Pillai A, Howell KR, Ahmed AO, Weinberg D, Allen KM, Bruggemann J*, et al*. Association of serum VEGF levels with prefrontal cortex volume in schizophrenia. *Mol Psychiatry* 2015.

2. Lee BH, Hong JP, Hwang JA, Ham BJ, Na KS, Kim WJ*, et al*. Alterations in plasma vascular endothelial growth factor levels in patients with schizophrenia before and after treatment. *Psychiatry Res* 2015; **228**(1)**:** 95-99.

3. Lee BH, Kim YK. Increased plasma VEGF levels in major depressive or manic episodes in patients with mood disorders. *J Affect Disord* 2012; **136**(1-2)**:** 181-184.

4. Takebayashi M, Hashimoto R, Hisaoka K, Tsuchioka M, Kunugi H. Plasma levels of vascular endothelial growth factor and fibroblast growth factor 2 in patients with major depressive disorders. *J Neural Transm (Vienna)* 2010; **117**(9)**:** 1119-1122.

5. Garbett K, Ebert PJ, Mitchell A, Lintas C, Manzi B, Mirnics K*, et al*. Immune transcriptome alterations in the temporal cortex of subjects with autism. *Neurobiol Dis* 2008; **30**(3)**:** 303-311.

6. Liu Z, Liu W, Yao L, Yang C, Xiao L, Wan Q*, et al*. Negative life events and corticotropin-releasing-hormone receptor1 gene in recurrent major depressive disorder. *Sci Rep* 2013; **3:** 1548.

7. Zhang B, Fan QR, Li WH, Lu N, Fu DK, Kang YJ*, et al*. Association of the NOTCH4 Gene Polymorphism rs204993 with Schizophrenia in the Chinese Han Population. *Biomed Res Int* **2015:** 408096.

8. Dieset I, Djurovic S, Tesli M, Hope S, Mattingsdal M, Michelsen A*, et al*. Up-regulation of NOTCH4 gene expression in bipolar disorder. *Am J Psychiatry* 2012; **169**(12)**:** 1292-1300.

9. Pietersen CY, Mauney SA, Kim SS, Passeri E, Lim MP, Rooney RJ*, et al*. Molecular profiles of parvalbumin-immunoreactive neurons in the superior temporal cortex in schizophrenia. *J Neurogenet* 2014; **28**(1-2)**:** 70-85.

10. Sun N, Lei L, Wang Y, Yang C, Liu Z, Li X*, et al*. Preliminary comparison of plasma notch-associated microRNA-34b and -34c levels in drug naive, first episode depressed patients and healthy controls. *J Affect Disord* 2016; **194:** 109-114.

11. Tsai MC, Liou CW, Lin TK, Lin IM, Huang TL. Bcl-2 associated with positive symptoms of schizophrenic patients in an acute phase. *Psychiatry Res* 2013; **210**(3)**:** 735-738.

12. Soeiro-de-Souza MG, Salvadore G, Moreno RA, Otaduy MC, Chaim KT, Gattaz WF*, et al*. Bcl-2 rs956572 polymorphism is associated with increased anterior cingulate cortical glutamate in euthymic bipolar I disorder. *Neuropsychopharmacology* 2013; **38**(3)**:** 468-475.

13. Chen WT, Huang TL, Tsai MC. Bcl-2 associated with severity of manic symptoms in bipolar patients in a manic phase. *Psychiatry Res* 2015; **225**(3)**:** 305-308.

14. Malik M, Sheikh AM, Wen G, Spivack W, Brown WT, Li X. Expression of inflammatory cytokines, Bcl2 and cathepsin D are altered in lymphoblasts of autistic subjects. *Immunobiology* 2011; **216**(1-2)**:** 80-85.

15. Einat H, Yuan P, Manji HK. Increased anxiety-like behaviors and mitochondrial dysfunction in mice with targeted mutation of the Bcl-2 gene: further support for the involvement of mitochondrial function in anxiety disorders. *Behav Brain Res* 2005; **165**(2)**:** 172-180.

16. Ma L, Wu DD, Ma SL, Tan L, Chen X, Tang NL*, et al*. Molecular evolution in the CREB1 signal pathway and a rare haplotype in CREB1 with genetic predisposition to schizophrenia. *J Psychiatr Res* 2014; **57:** 84-89.

17. Ren X, Rizavi HS, Khan MA, Bhaumik R, Dwivedi Y, Pandey GN. Alteration of cyclic-AMP response element binding protein in the postmortem brain of subjects with bipolar disorder and schizophrenia. *J Affect Disord* 2014; **152-154:** 326-333.

18. Zubenko GS, Hughes HB, 3rd, Stiffler JS, Brechbiel A, Zubenko WN, Maher BS*, et al*. Sequence variations in CREB1 cosegregate with depressive disorders in women. *Mol Psychiatry* 2003; **8**(6)**:** 611-618.

19. Burdick KE, DeRosse P, Kane JM, Lencz T, Malhotra AK. Association of genetic variation in the MET proto-oncogene with schizophrenia and general cognitive ability. *Am J Psychiatry* 2010; **167**(4)**:** 436-443.

20. Peng Y, Huentelman M, Smith C, Qiu S. MET receptor tyrosine kinase as an autism genetic risk factor. *Int Rev Neurobiol* 2013; **113:** 135-165.

21. Volk HE, Kerin T, Lurmann F, Hertz-Picciotto I, McConnell R, Campbell DB. Autism spectrum disorder: interaction of air pollution with the MET receptor tyrosine kinase gene. *Epidemiology* 2014; **25**(1)**:** 44-47.

22. Kerns D, Vong GS, Barley K, Dracheva S, Katsel P, Casaccia P*, et al*. Gene expression abnormalities and oligodendrocyte deficits in the internal capsule in schizophrenia. *Schizophr Res* 2010; **120**(1-3)**:** 150-158.

23. Leszczynska-Rodziewicz A, Maciukiewicz M, Szczepankiewicz A, Poglodzinski A, Hauser J. Association between OPCRIT dimensions and polymorphisms of HPA axis genes in bipolar disorder. *J Affect Disord* 2013; **151**(2)**:** 744-747.

24. Schatzberg AF, Keller J, Tennakoon L, Lembke A, Williams G, Kraemer FB*, et al*. HPA axis genetic variation, cortisol and psychosis in major depression. *Mol Psychiatry* 2014; **19**(2)**:** 220-227.

25. Gray JM, Vecchiarelli HA, Morena M, Lee TT, Hermanson DJ, Kim AB*, et al*. Corticotropin-releasing hormone drives anandamide hydrolysis in the amygdala to promote anxiety. *J Neurosci* 2015; **35**(9)**:** 3879-3892.
