# Supplementary material for: Preliminary evidence for association of genetic variants in pri-miR-34b/c and abnormal miR-34c expression with attention deficit and hyperactivity disorder
Source: Transl Psychiatry. 2016 Aug 30;6(8):e879–. doi: 10.1038/tp.2016.151 (PMC5022091; doi:10.1038/tp.2016.151)
Supplement: Supplementary Table 2 [file tp2016151x3.doc]

**Supplementary Table 2** Description of SNPs in 134 miRNA gene regions that are candidate for ADHD

| **MicroRNA** | **Host Gene ID** | **Region (NCBI36/hg18)** | **SNPs** | **Tag SNPs** | **SNP ID** | **Position** | **Alleles** | **Exclusion criteria** | **Other SNPs within the bin** |
| --- | --- | --- | --- | --- | --- | --- | --- | --- | --- |
| **hsa-let-7a-1** | ***MIRLET7DHG*** | 9:95973060-95984023 | 9 | 3 | rs7865876 | 95973072 | A/G |  | rs10739971, rs10821311, rs2039012, rs10993095, rs12004367 |
| **hsa-let-7f-1** |  | 9:96006669-96011669 |  |  | rs8115 | [95981398](https://genome-euro.ucsc.edu/cgi-bin/hgTracks?hgsid=198763903_OHmuiB75Zh5KETNmeUxviwz6eLPX&db=hg18&position=chr9%3A95981398-95981398) | A/G |  | rs4744326 |
| **hsa-let-7d** |  |  |  |  | rs7872931 | [96010018](https://genome-euro.ucsc.edu/cgi-bin/hgTracks?hgsid=198763903_OHmuiB75Zh5KETNmeUxviwz6eLPX&db=hg18&position=chr9%3A96010018-96010018) | A/G |  |  |
| **hsa-miR-98** | ***HUWE1*** | X:53596909-53605960 | 1 | 1 | rs12853099 | 53735378 | A/C |  |  |
| **hsa-let-7f-2** |  | X:53730422-53735422 |  |  |  |  |  |  |  |
| **hsa-let-7a-3** | ***MIRLET7BHG*** | 22:44882293-44891312 | 7 | 5 | rs3859891 | 44882432 | G/T |  | rs7288847, rs6007862 |
| **hsa-mir-4763** |  | 22:44855541-44860541 |  |  | rs6520050 | [44856859](https://genome-euro.ucsc.edu/cgi-bin/hgTracks?hgsid=198763903_OHmuiB75Zh5KETNmeUxviwz6eLPX&db=hg18&position=chr22%3A44856859-44856859) | A/G |  |  |
| **hsa-let-7b** |  |  |  |  | rs9616084 | [44859266](https://genome-euro.ucsc.edu/cgi-bin/hgTracks?hgsid=198763903_OHmuiB75Zh5KETNmeUxviwz6eLPX&db=hg18&position=chr22%3A44859266-44859266) | C/T |  |  |
|  |  |  |  |  | rs11090910 | [44886334](https://genome-euro.ucsc.edu/cgi-bin/hgTracks?hgsid=198763903_OHmuiB75Zh5KETNmeUxviwz6eLPX&db=hg18&position=chr22%3A44886334-44886334) | C/T |  |  |
|  |  |  |  |  | rs4823529 | [44889405](https://genome-euro.ucsc.edu/cgi-bin/hgTracks?hgsid=198763903_OHmuiB75Zh5KETNmeUxviwz6eLPX&db=hg18&position=chr22%3A44889405-44889405) | A/G | Monomorphic |  |
| **hsa-let-7a-2** | ***MIR100HG*** | 11:121519440-121533226 | 22 | 14 | rs564037 | [121519609](https://genome-euro.ucsc.edu/cgi-bin/hgTracks?hgsid=198763903_OHmuiB75Zh5KETNmeUxviwz6eLPX&db=hg18&position=chr11%3A121519609-121519609) | C/T |  | rs1143770, rs1834306 |
| **hsa-miR-100** |  | 11:121472675-21480762 |  |  | rs629367 | [121522224](https://genome-euro.ucsc.edu/cgi-bin/hgTracks?hgsid=198763903_OHmuiB75Zh5KETNmeUxviwz6eLPX&db=hg18&position=chr11%3A121522224-121522224) | A/C |  | rs537989 |
| **hsa-miR-125b-1** |  | 11:121578980-121583980 |  |  | rs562052 | [121523760](https://genome-euro.ucsc.edu/cgi-bin/hgTracks?hgsid=198763903_OHmuiB75Zh5KETNmeUxviwz6eLPX&db=hg18&position=chr11%3A121523760-121523760) | A/G |  | rs564900, rs689025, rs543412 |
|  |  |  |  |  | rs12421791 | [121524471](https://genome-euro.ucsc.edu/cgi-bin/hgTracks?hgsid=198763903_OHmuiB75Zh5KETNmeUxviwz6eLPX&db=hg18&position=chr11%3A121524471-121524471) | C/T |  | rs4936674, rs1364757 |
|  |  |  |  |  | rs2081443 | [121475956](https://genome-euro.ucsc.edu/cgi-bin/hgTracks?hgsid=198763903_OHmuiB75Zh5KETNmeUxviwz6eLPX&db=hg18&position=chr11%3A121475956-121475956) | G/T |  |  |
|  |  |  |  |  | rs2241490 | [121475990](https://genome-euro.ucsc.edu/cgi-bin/hgTracks?hgsid=198763903_OHmuiB75Zh5KETNmeUxviwz6eLPX&db=hg18&position=chr11%3A121475990-121475990) | C/T |  |  |
|  |  |  |  |  | rs512932 | [121478751](https://genome-euro.ucsc.edu/cgi-bin/hgTracks?hgsid=198763903_OHmuiB75Zh5KETNmeUxviwz6eLPX&db=hg18&position=chr11%3A121478751-121478751) | A/G |  |  |
|  |  |  |  |  | rs547008 | [121523071](https://genome-euro.ucsc.edu/cgi-bin/hgTracks?hgsid=198763903_OHmuiB75Zh5KETNmeUxviwz6eLPX&db=hg18&position=chr11%3A121523071-121523071) | C/T |  |  |
|  |  |  |  |  | rs560279 | [121528721](https://genome-euro.ucsc.edu/cgi-bin/hgTracks?hgsid=198763903_OHmuiB75Zh5KETNmeUxviwz6eLPX&db=hg18&position=chr11%3A121528721-121528721) | C/T | Failed genotyping assay |  |
|  |  |  |  |  | rs17126230 | [121530955](https://genome-euro.ucsc.edu/cgi-bin/hgTracks?hgsid=198763903_OHmuiB75Zh5KETNmeUxviwz6eLPX&db=hg18&position=chr11%3A121530955-121530955) | A/G | Monomorphic |  |
|  |  |  |  |  | rs638742 | [121531148](https://genome-euro.ucsc.edu/cgi-bin/hgTracks?hgsid=198763903_OHmuiB75Zh5KETNmeUxviwz6eLPX&db=hg18&position=chr11%3A121531148-121531148) | C/G |  |  |
|  |  |  |  |  | rs1816158 | [121531670](https://genome-euro.ucsc.edu/cgi-bin/hgTracks?hgsid=198763903_OHmuiB75Zh5KETNmeUxviwz6eLPX&db=hg18&position=chr11%3A121531670-121531670) | C/T |  |  |
|  |  |  |  |  | rs7928250 | 121531699 | A/C | Failed genotyping assay |  |
|  |  |  |  |  | rs10892819 | 121579254 | G/T |  |  |
| **hsa-miR-99a** | ***LINC00478*** | 21:16828280-16837102 | 9 | 4 | rs2823592 | 16360212 | C/T |  | rs2823594, rs12152048 |
| **hsa-let-7c** |  | 21:16879428-16887516 |  |  | rs2823593 | 16362617 | A/T |  | rs2823595 |
| **hsa-miR-125b-2** |  | 21:16359713-16364713 |  |  | rs2051347 | 16485140 | A/C |  | rs2823634, rs2823635 |
|  |  | 21:16483570-16488570 |  |  | rs7279730 | 16883415 | A/G |  |  |
| **hsa-miR-122** |  | 18:54264286-54272370 | 13 | 6 | rs4245271 | 54265043 | A/G |  | rs4245272, rs4940703, rs9319929, rs1135519, rs17669, rs6566969, rs2277722 |
|  |  |  |  |  | rs12968521 | 54266244 | C/T |  |  |
|  |  |  |  |  | rs4940704 | 54266487 | A/G |  |  |
|  |  |  |  |  | rs10514884 | 54270132 | A/G |  |  |
|  |  |  |  |  | rs11663046 | 54270609 | C/G |  |  |
|  |  |  |  |  | rs10514883 | 54271634 | A/G |  |  |
| **hsa-miR-124-1** |  | 8:9795308-9803392 | 4 | 3 | rs491364 | 9795640 | A/G |  | rs592420 |
|  |  |  |  |  | rs531564 | 9798109 | C/G |  |  |
| **hsa-miR-124-2** |  | 8:65449260-65457368 | 1 | 1 | rs298212 | 65453204 | C/T |  |  |
| **hsa-miR-124-3** |  | 20:61275297-61283383 | 3 | 2 | rs6122390 | 61275709 | C/T |  | rs2064611 |
|  |  |  |  |  | rs2064612 | 61283377 | C/T |  |  |
| **hsa-miR-128-1** | ***R3HDM1*** | 2:136134437-136142518 | 6 | 4 | rs1446584 | 136136431 | C/T |  | rs4954280, rs2034276 |
|  |  | 2:136000553-136005553 |  |  | rs3806502 | 136004743 | A/G |  |  |
|  |  |  |  |  | rs1374330 | 136137378 | G/T |  |  |
|  |  |  |  |  | rs2289959 | 136140374 | A/G |  |  |
| **hsa-miR-128-2** | ***ARPP21*** | 3:35755972-35764055 | 23 | 11 | rs1542211 | 35653498 | A/G | Failed genotyping assay | rs17280201, rs962038 |
|  |  | 3:35652514-35657514 |  |  | rs9878558 | 35693118 | C/T |  | rs4678793, rs467879 |
|  |  | 3:35653853-35658853 |  |  | rs7621692 | 35756766 | G/T |  | rs7621392, rs7643820, rs13100210, rs10490873, rs6779091, rs4678794, rs2305234 |
|  |  | 3:35691283-35696283 |  |  | rs9311107 | 35759327 | C/T |  | rs6550367 |
|  |  |  |  |  | rs7612999 | 35653341 | A/G |  |  |
|  |  |  |  |  | rs9860326 | 35658108 | C/G |  |  |
|  |  |  |  |  | rs4678788 | 35658440 | A/G |  |  |
|  |  |  |  |  | rs1513474 | 35695571 | C/T |  |  |
|  |  |  |  |  | rs13094224 | 35759585 | A/G |  |  |
|  |  |  |  |  | rs11712442 | 35760210 | C/G | Failed genotyping assay |  |
|  |  |  |  |  | rs6801590 | 35762465 | A/C |  |  |
| **hsa-miR-133a-1** |  | 18:17656657-17668047 | 5 | 3 | rs2155975 | 17661399 | A/G |  | rs3810042 |
| **hsa-mir-1-2** |  |  |  |  | rs4591246 | 17662527 | A/G |  | rs4467173 |
|  |  |  |  |  | rs12968949 | 17658855 | C/T | Failed genotyping assay |  |
| **hsa-mir-1-1** | ***C20orf166*** | 20:60556958-60565028 | 21 | 11 | rs3934574 | 60553932 | C/T |  | rs6062277, rs6062278, rs8122282, rs6062267,rs6089741 |
| **hsa-miR-133a-2** |  | 20:60567564-60575665 |  |  | rs6062238 | 60560140 | A/G |  | rs6062266, rs6062264 |
|  |  | 20:60553105-60558105 |  |  | rs6062261 | 60565961 | C/T |  | rs6089746 |
|  |  |  |  |  | rs4969531 | 60571691 | C/T |  | rs4072471 |
|  |  |  |  |  | rs4072474 | 60574467 | A/G |  | rs11698727 |
|  |  |  |  |  | rs12479469 | 60555641 | C/T |  |  |
|  |  |  |  |  | rs6062275 | 60557354 | A/G |  |  |
|  |  |  |  |  | rs6122011 | 60567391 | A/C |  |  |
|  |  |  |  |  | rs4246446 | 60569315 | A/G |  |  |
|  |  |  |  |  | rs11906462 | 60569397 | A/G |  |  |
|  |  |  |  |  | rs6062235 | 60570696 | A/G |  |  |
| **hsa-miR-137** | ***MIR137HG*** | 1:98281214-98289315 | 2 | 2 | rs2660304 | 98284715 | A/C |  |  |
|  |  | 1:98287837-98292837 |  |  | rs2660303 | 98285732 | A/T |  |  |
| **hsa-miR-200c** |  | 12:6938123-6946615 | 2 | 2 | rs759052 | 6939881 | C/T | Monomorphic |  |
| **hsa-miR-141** |  |  |  |  | rs2159887 | 6946497 | C/G |  |  |
| **hsa-miR-146a** |  | 5:159839937-159848035 | 7 | 3 | rs2910164 | 159844996 | C/G |  | rs2910163, rs2961920, rs4921290,rs17057868 |
|  |  |  |  |  | rs3096021 | 159846181 | C/T |  |  |
|  |  |  |  |  | rs6878034 | 159847699 | C/T |  |  |
| **hsa-miR-148a** |  | 7:43509540-43517619 | 12 | 4 | rs17208701 | 43514667 | A/G |  | rs9639877, rs17172220, rs10253986, rs17208834, rs17208856, rs3823863 |
|  |  |  |  |  | rs11767004 | 43515566 | A/G |  | rs11760314 |
|  |  |  |  |  | rs11763891 | 43515643 | C/T |  | rs3801397 |
|  |  |  |  |  | rs17151028 | 43511032 | A/G |  |  |
| **hsa-miR-150** |  | 19:54692854-54700937 | 4 | 1 | rs739347 | 54693197 | C/T |  | rs2073614, rs739349, rs2077300 |
| **hsa-miR-155** | ***MIR155HG*** | 21:25863163-25871227 | 3 | 3 | rs1740085 | 25851706 | A/G |  |  |
|  |  | 21:25851328-25856328 |  |  | rs928883 | 25865896 | A/G |  |  |
|  |  |  |  |  | rs2829803 | 25870181 | A/G |  |  |
| **hsa-miR-16-1** | ***DLEU2*** | 13:49518110-49526338 | 2 | 2 | rs9535416 | 49522141 | A/G |  |  |
| **hsa-miR-15a** |  | 13:49597678-49602678 |  |  | rs7998223 | 49526200 | C/T |  |  |
| **hsa-miR-15b** | ***SMC4*** | 3:161600070-161608307 | 5 | 3 | rs6793560 | 161596163 | A/T |  | rs4680580, rs7634108 |
| **hsa-miR-16-2** |  | 3:161595124-161600124 |  |  | rs10936201 | 161604533 | A/C |  |  |
|  |  | 3:161596041-161601041 |  |  | rs4679883 | 161608297 | A/G |  |  |
| **hsa-miR-17** | ***MIR17HG*** | 13:90795860-90804646 | 6 | 3 | rs9523310 | 90793183 | A/T |  | rs932910, rs4284505 |
| **hsa-miR-18a** |  | 13:90793075-90798075 |  |  | rs17556509 | 90793571 | C/T | Monomorphic | rs17642969 |
| **hsa-miR-19a** |  |  |  |  | rs7318578 | 90803470 | A/C |  |  |
| **hsa-miR-20a** |  |  |  |  |  |  |  |  |  |
| **hsa-miR-19b-1** |  |  |  |  |  |  |  |  |  |
| **hsa-miR-92a-1** |  |  |  |  |  |  |  |  |  |
| **hsa-miR-181b-1** | ***LOC100131234*** | 1:197091625-197099905 | 3 | 1 | rs10919595 | 197093847 | A/G |  | rs1322923, rs1275158 |
| **hsa-miR-181a-1** |  | 1:197173181-197178181 |  |  |  |  |  |  |  |
| **hsa-miR-181a-2** | ***MIR181A2HG*** | 9:126489542-126498898 | 4 | 1 | rs4838200 | 126490878 | C/T |  | rs10760371, rs3739760, rs6478680 |
| **hsa-miR-181b-2** |  | 9:126455536-126460536 |  |  |  |  |  |  |  |
| **hsa-miR-199a-1** |  | 19:10786102-10794172 | 2 | 2 | rs3786719 | 10788100 | C/G |  |  |
|  |  |  |  |  | rs11085748 | 10788540 | C/T |  |  |
| **hsa-miR-214** |  | 1:170371561-170385407 | 8 | 3 | rs12047492 | 170377388 | A/G |  | rs10911101, rs12120556, rs10911109, rs10911110, rs10911111, rs10911112 |
| **hsa-miR-199a-2** |  |  |  |  | rs17277008 | 170371785 | C/T |  |  |
|  |  |  |  |  | rs2819530 | 170379577 | A/G |  |  |
| **hsa-miR-200b** |  | 1:1087347-1097330 | 2 | 2 | rs4970420 | 1096336 | A/G |  |  |
| **hsa-miR-200a** |  |  |  |  | rs4442317 | 1096647 | C/T |  |  |
| **hsa-miR-429** |  |  |  |  |  |  |  |  |  |
| **hsa-miR-21** |  | 17:55268409-55276480 | 4 | 3 | rs1292037 | 55273690 | A/G |  | rs13137 |
|  |  |  |  |  | rs1292060 | 55268631 | C/T |  |  |
|  |  |  |  |  | rs2645479 | 55275314 | C/T | No HWE in controls |  |
| **hsa-miR-210** | ***MIR210HG*** | 11:555089-563198 | 7 | 5 | rs3740651 | 556300 | C/T |  | rs7941198 |
|  |  | 11:558457-563457 |  |  | rs7927267 | 557391 | C/T |  | rs7935908 |
|  |  |  |  |  | rs1599725 | 555129 | A/G |  |  |
|  |  |  |  |  | rs1056812 | 555840 | A/G | Monomorphic |  |
|  |  |  |  |  | rs12802200 | 556936 | A/C |  |  |
| **hsa-miR-217** |  | 2:56060606-56074698 | 10 | 2 | rs11690813 | 56061663 | A/G |  | rs1835897, rs10204798, rs6756818,rs6545535, rs6545537, rs6545538, rs1017371, rs1432553 |
| **hsa-miR-216a** |  |  |  |  | rs2009613 | 56072032 | A/G |  |  |
| **hsa-miR-218-1** | ***SLIT2*** | 4:20133996-20142105 | 9 | 8 | rs614158 | 19860215 | A/G |  | rs564041 |
|  |  | 4:19859333-19864333 |  |  | rs7663557 | 19860616 | A/G |  |  |
|  |  |  |  |  | rs4552453 | 19863396 | C/T |  |  |
|  |  |  |  |  | rs7690660 | 20136962 | G/T |  |  |
|  |  |  |  |  | rs3775815 | 20138768 | G/T |  |  |
|  |  |  |  |  | rs9992559 | 20139472 | C/T |  |  |
|  |  |  |  |  | rs2168801 | 20140079 | A/G |  |  |
|  |  |  |  |  | rs12504587 | 20141487 | A/T |  |  |
| **hsa-miR-218-2** | ***SLIT3*** | 5:168124729-168132838 | 23 | 15 | rs17070433 | 168125060 | A/G | Monomorphic | rs17070438, rs6877545, rs17070443, rs2288794 |
|  |  | 5:168660711-168665711 |  |  | rs6860336 | 168126456 | C/T |  | rs11134527, rs9784690, rs1368355 |
|  |  |  |  |  | rs6860854 | 168126754 | C/T |  | rs11746483 |
|  |  |  |  |  | rs1014194 | 168125522 | G/T |  |  |
|  |  |  |  |  | rs17634871 | 168126668 | C/T |  |  |
|  |  |  |  |  | rs17553555 | 168128446 | C/T | Monomorphic |  |
|  |  |  |  |  | rs6555826 | 168130042 | A/C |  |  |
|  |  |  |  |  | rs2337556 | 168130237 | C/T | Failed primer design |  |
|  |  |  |  |  | rs1875976 | 168130379 | C/T |  |  |
|  |  |  |  |  | rs2082400 | 168130404 | C/T |  |  |
|  |  |  |  |  | rs868103 | 168130883 | A/G |  |  |
|  |  |  |  |  | rs2053046 | 168131545 | A/G |  |  |
|  |  |  |  |  | rs10516050 | 168131879 | C/T |  |  |
|  |  |  |  |  | rs4867902 | 168132202 | C/T |  |  |
|  |  |  |  |  | rs1422028 | 168663218 | C/G |  |  |
| **hsa-miR-221** |  | X:45487529-45496474 | 4 | 3 | rs2858059 | 45492134 | C/T |  | rs2745708 |
| **hsa-miR-222** |  |  |  |  | rs2858060 | 45491952 | C/G |  |  |
|  |  |  |  |  | rs4824557 | 45494058 | A/G |  |  |
| **hsa-miR-24-2** |  | 19:13805101-13813473 | 5 | 2 | rs895819 | 13808292 | C/T |  | rs12979166, rs12978798, rs2594716 |
| **hsa-miR-27a** |  |  |  |  | rs1531212 | 13812830 | C/T |  |  |
| **hsa-miR-23a** |  |  |  |  |  |  |  |  |  |
| **hsa-miR-26b** | ***CTDSP1*** | 2:218970613-218978689 | 10 | 4 | rs2279015 | 218967514 | C/T |  | rs1059823 |
|  |  | 2:218966305-218971305 |  |  | rs13062 | 218968895 | A/C |  | rs2279014 |
|  |  | 2:218967722-218972722 |  |  | rs2739049 | 218972351 | A/G |  | rs2252235, rs2227255, rs2227258, rs2579959 |
|  |  |  |  |  | rs2227251 | 218974667 | A/C | Failed genotyping assay |  |
| **hsa-miR-29a** |  | 7:130209046-130217838 | 9 | 7 | rs11978062 | 130210805 | A/G |  | rs157906 |
| **hsa-miR-29b-1** |  |  |  |  | rs157908 | 130215902 | C/T |  | rs2293746 |
|  |  |  |  |  | rs207217 | 130210863 | C/T |  |  |
|  |  |  |  |  | rs207218 | 130211319 | C/G | Failed genotyping assay |  |
|  |  |  |  |  | rs24168 | 130211825 | C/T |  |  |
|  |  |  |  |  | rs7781163 | 130213364 | C/T |  |  |
|  |  |  |  |  | rs157907 | 130213977 | A/G |  |  |
| **hsa-miR-29c** |  | 1:206038820-206047491 | 5 | 2 | rs2724377 | 206041441 | C/T |  | rs2745972 |
| **hsa-miR-29b-2** |  |  |  |  | rs1474742 | 206043289 | A/C |  | rs7523273, rs2796283 |
| **hsa-miR-367** |  | 4:113785479-113794162 | 2 | 2 | rs13136737 | 113788584 | G/T |  |  |
| **hsa-miR-302d** |  |  |  |  | rs4383675 | 113792068 | A/G |  |  |
| **hsa-miR-302a** |  |  |  |  |  |  |  |  |  |
| **hsa-miR-302c** |  |  |  |  |  |  |  |  |  |
| **hsa-miR-302b** |  |  |  |  |  |  |  |  |  |
| **hsa-miR-30a** |  | 6:72166975-72175045 | 4 | 3 | rs2222722 | 72170433 | C/T |  | rs9283841 |
|  |  |  |  |  | rs6921100 | 72167425 | C/G |  |  |
|  |  |  |  |  | rs13202946 | 72169312 | C/T |  |  |
| **hsa-miR-335** | ***MEST*** | 7:129918188-129926281 | 7 | 2 | rs17391475 | 129908597 | A/T |  | rs13245645, rs1421140 |
|  |  | 7:129908252-129913252 |  |  | rs12706940 | 129916029 | C/T |  | rs2301335, rs2072573, rs2072574 |
|  |  | 7:129913406-129918406 |  |  |  |  |  |  |  |
|  |  | 7:129908424-129913424 |  |  |  |  |  |  |  |
|  |  | 7:129914135-129919135 |  |  |  |  |  |  |  |
| **hsa-miR-34a** |  | 1:9131314-9139423 | 4 | 3 | rs4281343 | 9132545 | A/G |  | rs17393144 |
|  |  |  |  |  | rs12128240 | 9133890 | C/T |  |  |
|  |  |  |  |  | rs7529468 | 9139048 | G/T | No HWE in controls |  |
| **hsa-miR-34b** |  | 11:110883873-110892450 | 4 | 3 | rs4938723 | 110887775 | C/T |  | rs2187388 |
| **hsa-miR-34c** |  |  |  |  | rs2187473 | 110889056 | A/G | Failed genotyping assay |  |
|  |  |  |  |  | rs28690953 | 110889636 | G/T |  |  |
| **hsa-miR-371** |  | 19:58977741-58986839 | 10 | 10 | rs10427077 | 58978708 | C/G |  |  |
| **hsa-miR-372** |  |  |  |  | rs3848577 | 58980313 | C/T |  |  |
| **hsa-miR-373** |  |  |  |  | rs3848580 | 58980658 | C/G |  |  |
|  |  |  |  |  | rs12460972 | 58982309 | A/G |  |  |
|  |  |  |  |  | rs12983508 | 58982532 | C/G |  |  |
|  |  |  |  |  | rs3859501 | 58983223 | A/C |  |  |
|  |  |  |  |  | rs12983273 | 58983644 | C/T |  |  |
|  |  |  |  |  | rs12978005 | 58985130 | C/T | Failed genotyping assay |  |
|  |  |  |  |  | rs8103186 | 58985453 | A/C |  |  |
|  |  |  |  |  | rs3859503 | 58986212 | G/T |  |  |
| **hsa-mir-381** |  | 14: 100577010-100605891 | 25 | 11 | rs10083406 | 100582333 | A/C |  | rs12100867, rs10144193, rs1951032, rs4143957, rs7342570, rs6575812, rs11160619 |
| **hsa-mir-487b** |  |  |  |  | rs7161441 | 100582998 | G/T |  | rs10132916, rs12886869 |
| **hsa-mir-539** |  |  |  |  | rs4906032 | 100588663 | A/G |  | rs4525426, rs8003403, rs4906033, rs4906034, rs12893725 |
| **hsa-mir-889** |  |  |  |  | rs8023048 | 100598895 | A/C |  |  |
| **hsa-mir-544a** |  |  |  |  | rs7161194 | 100598758 | A/G |  |  |
| **hsa-mir-655** |  |  |  |  | rs8015875 | 100603041 | A/G |  |  |
| **hsa-mir-487a** |  |  |  |  | rs8016185 | 100603137 | C/T |  |  |
| **hsa-mir-382** |  |  |  |  | rs10151229 | 100604060 | A/G |  |  |
| **hsa-mir-134** |  |  |  |  | rs4900482 | 100604512 | C/T | No HWE in controls |  |
| **hsa-mir-668** |  |  |  |  | rs2007291 | 100605709 | A/T |  |  |
| **hsa-miR-485** |  |  |  |  | rs941714 | 100605882 | C/T |  |  |
| **hsa-mir-323b** |  |  |  |  |  |  |  |  |  |
| **hsa-mir-154** |  |  |  |  |  |  |  |  |  |
| **hsa-mir-496** |  |  |  |  |  |  |  |  |  |
| **hsa-miR-377** |  |  |  |  |  |  |  |  |  |
| **hsa-mir-541** |  |  |  |  |  |  |  |  |  |
| **hsa-mir-409** |  |  |  |  |  |  |  |  |  |
| **hsa-mir-412** |  |  |  |  |  |  |  |  |  |
| **hsa-mir-369** |  |  |  |  |  |  |  |  |  |
| **hsa-mir-410** |  |  |  |  |  |  |  |  |  |
| **hsa-mir-656** |  |  |  |  |  |  |  |  |  |
| **hsa-mir-450b** | ***MGC16121*** | X:133498881-133513407 | 4 | 2 | rs2503985 | 133501182 | A/G |  | rs757308, rs17396 |
| **hsa-mir-450a-1** |  | X:133508326-133513326 |  |  | rs757309 | 133508213 | A/G |  |  |
| **hsa-mir-450a-2** |  |  |  |  |  |  |  |  |  |
| **hsa-mir-542** |  |  |  |  |  |  |  |  |  |
| **hsa-miR-503** |  |  |  |  |  |  |  |  |  |
| **hsa-miR-424** |  |  |  |  |  |  |  |  |  |
| **hsa-mir-514b** |  | X:146136361-146178938 | 23 | 8 | rs5951956 | 146136523 | A/G |  | rs5951957, rs5905019, rs5905021, rs5905022, rs5951787 |
| **hsa-mir-509-2** |  |  |  |  | rs5905023 | 146144653 | G/T |  | rs5904727, rs5905024 |
| **hsa-mir-509-3** |  |  |  |  | rs2504174 | 146148226 | A/G |  | rs2495248 |
| **hsa-mir-509-1** |  |  |  |  | rs1597029 | 146158289 | A/G |  | rs2392720 |
| **hsa-miR-510** |  |  |  |  | rs5905027 | 146159943 | G/T |  | rs5905029, rs5905031, rs12008050, rs11094416, rs12559800, rs5905033 |
| **hsa-mir-514a-1** |  |  |  |  | rs2442040 | 146159067 | A/C |  |  |
| **hsa-mir-514a-2** |  |  |  |  | rs2504169 | 146165697 | G/T |  |  |
| **hsa-mir-514a-3** |  |  |  |  | rs5905034 | 146176797 | A/G |  |  |
| **hsa-miR-625** | ***FUT8*** | 14:65002573-65010657 | 4 | 3 | rs2411822 | 64948148 | C/T |  | rs1953416 |
|  |  | 14:64944201-64949201 |  |  | rs7147536 | 64944451 | C/T |  |  |
|  |  | 14:64942063-64947063 |  |  | rs7141367 | 65008394 | C/T |  |  |
| **hsa-miR-765** | ***ARHGEF11-1*** | 1:155169547-155177660 | 9 | 6 | rs3187878 | 155171268 | C/T |  | rs6676, rs3765788, rs3818807, rs2275199, rs12402294, rs12406029 |
|  |  | 1:155281786-155286786 |  |  | rs945508 | 155173705 | A/G |  |  |
|  |  |  |  |  | rs703111 | 155285899 | A/T |  |  |
| **hsa-miR-802** |  | 21:36009883-36017976 | 3 | 2 | rs1014803 | 36015910 | A/G |  | rs1022417 |
|  |  |  |  |  | rs2835104 | 36013145 | A/G |  |  |
| **hsa-miR-9-1** | ***C1orf61*** | 1:154653757-154661845 | 4 | 4 | rs16837375 | 154653791 | C/G |  |  |
|  |  | 1:154665808-154670808 |  |  | rs7548323 | 154660624 | A/G |  |  |
|  |  |  |  |  | rs10494305 | 154666684 | A/G | Monomorphic |  |
|  |  |  |  |  | rs12138127 | 154670305 | C/G |  |  |
| **hsa-miR-9-2** | ***LINC00461*** | 5:87995427-88003513 | 6 | 4 | rs1501673 | 87999356 | C/T | Failed genotyping assay | rs1501672, rs16903285 |
|  |  | 5:88010018-88015018 |  |  | rs6893807 | 88000777 | A/G |  |  |
|  |  | 5:88004902-88009902 |  |  | rs153685 | 88016786 | C/T |  |  |
|  |  | 5:88016376-88021376 |  |  | rs26579 | 88021051 | C/G |  |  |
| **hsa-miR-9-3** |  | 15:87707252-87715341 | 1 | 1 | rs176644 | 87714636 | G/T |  |  |
| **hsa-mir-182** |  | 7:129194459-129207090 | 5 | 3 | rs4626538 | 129200424 | G/T |  | rs4335057, rs12538588 |
| **hsa-miR-96** |  |  |  |  | rs6467264 | 129194497 | C/T |  |  |
| **hsa-mir-183** |  |  |  |  | rs6965643 | 129194800 | A/G |  |  |

*****When the miRNAs are intragenic and share transcriptional sense with the host gene, ***Host*** ***Gene ID*** is specified and additional promoter region was considered in the study.

****** All positions are based on in the **NCBI36/hg18** human genome build (Mar. 2006).
