# Supplementary material for: Preliminary evidence for association of genetic variants in pri-miR-34b/c and abnormal miR-34c expression with attention deficit and hyperactivity disorder
Source: Transl Psychiatry. 2016 Aug 30;6(8):e879–. doi: 10.1038/tp.2016.151 (PMC5022091; doi:10.1038/tp.2016.151)
Supplement: Supplementary Table 3 [file tp2016151x4.doc]

**Supplementary Table 3** Nominal associations identified in the gene-based association analysis using VEGAS software and considering regions encoding (**a**) miRNA or (**b**) miRNA target genes containing more than one genotyped SNP per region.

| **miRNA or miRNA cluster** | **miRNA Host Gene** | **SNPs (risk allele)** | **P-value in single-marker analysis** | **VEGAS2 P-value in the gene-based analysis** | **Number of SNPs included in the gene-based analysis** |
| --- | --- | --- | --- | --- | --- |
| ***hsa-let-7a-1/hsa-let-7f-1/hsa-let-7d*** | ***MIRLET7DHG*** | rs7865876 (G)  rs8115 (G)  rs7872931 | 4.7e-03  0.032  0.058 | 2.9e-03* | 3 |
| ***hsa-miR-34b/hsa-miR-34c*** |  | rs28690953 (G)  rs4938723  rs2187473 | 8.9e-04  0.252  Failed genotyping assay | 2.8e-04* | 2 |

**(a)**

| **Target gene** | **miRNA Host Gene** | **SNPs (risk allele)** | **P-value in single-marker analysis** | **VEGAS2 P-value in the gene-based analysis** | **Number of SNPs included in the gene-based analysis** |
| --- | --- | --- | --- | --- | --- |
| ***MET*** |  | rs1621 (G)  rs6566 (G) | 8.3e-03  0.017 | 5.6e-03* | 2 |
| ***HMGA2*** |  | rs11175982 (T)  rs8756 | 7.5e-03  0.055 | 7.1e-03* | 2 |

**(b)**
