# Supplementary material for: Preliminary evidence for association of genetic variants in pri-miR-34b/c and abnormal miR-34c expression with attention deficit and hyperactivity disorder
Source: Transl Psychiatry. 2016 Aug 30;6(8):e879–. doi: 10.1038/tp.2016.151 (PMC5022091; doi:10.1038/tp.2016.151)
Supplement: Supplementary Table 4 [file tp2016151x5.doc]

**Supplementary Table 4**  Description of SNPs in 3’UTRs of target genes for miR-34b/c.

| **Target Gene** | **Contig Reference** | **Position (NCBI36/hg18)** | **Length (bp)** | **SNPs** | **Tag SNPs** | **SNP ID** | **Position** | **Alleles** | **Exclusion criteria** | **Other SNPs within the bin** |
| --- | --- | --- | --- | --- | --- | --- | --- | --- | --- | --- |
| ***BCL2*** | NM_000633 | 18:58941559-58946837 | 5279 | 6 | 4 | rs4987856 | 58944474 | A/G | Monomorphic | rs4987855, rs4987845 |
|  |  |  |  |  |  | rs4987853 | 58944635 | A/G |  |  |
|  |  |  |  |  |  | rs1564483 | 58945634 | A/G |  |  |
|  |  |  |  |  |  | rs4987843 | 58946521 | A/G |  | rs1016860 |
| ***CREB1*** | NM_134442 | 2:208170028-208178532 | 8502 | 9 | 2 | rs13029936 | 208173789 | C/T |  | rs2551928, rs1045780, rs6785, rs2551929, rs2256941, rs2551930 |
|  |  |  |  |  |  | rs56325368 | 208171964 | A/G | Failed primer design | rs1806585 |
| ***CRHR1*** | NM_001145146 | 17:41267912-41268975 | 1064 | 7 | 3 | rs2316765 | 41268235 | C/T |  | rs28364021 |
|  |  |  |  |  |  | rs878886 | 41268271 | C/G |  | rs878887, rs878888, rs4640231 |
|  |  |  |  |  |  | rs4525537 | 41268504 | C/T |  |  |
| ***HMGA2*** | NM_003483 | 12:64643340-64646342 | 2999 | 3 | 2 | rs8756 | 64646019 | A/C |  | rs1042725 |
|  |  |  |  |  |  | rs11175982 | 64646335 | C/T |  |  |
| ***JAG1*** | NM_000214 | 20:10566332-10568145 | 1814 | 2 | 2 | rs8708 | 10566574 | A/G | Failed primer design |  |
|  |  |  |  |  |  | rs7828 | 10567014 | G/T |  |  |
| ***MET*** | NM_001127500 | 7:116223415-116225678 | 2262 | 4 | 2 | rs6566 | 116225654 | A/G |  | rs41738, rs41739 |
|  |  |  |  |  |  | rs1621 | 116224842 | A/G |  |  |
| ***NOTCH1*** | NM_017617 | 9:138508717-138510343 | 1627 | 1 | 1 | rs6563 | 138509005 | A/G |  |  |
| ***NOTCH2*** | NM_024408 | 1:120255697-120259451 | 3753 | 4 | 1 | rs699779 | 120256833 | C/T |  | rs835576, rs835575, rs699780 |
| ***NOTCH3*** | NM_000435 | 19:15131444-15132472 | 1029 | 4 | 2 | rs1044116 | 15131665 | A/G |  | rs1044123, rs1044055 |
|  |  |  |  |  |  | rs7247906 | 15132135 | G/T | Failed primer design |  |
| ***VEGFA*** | NM_001025366 | 6:43860278-43862202 | 1925 | 3 | 2 | rs3025040 | 43861029 | C/T |  | rs3025039 |
|  |  |  |  |  |  | rs10434 | 43861190 | A/G | No HWE in controls |  |

***** All positions are based on in the **NCBI36/hg18** human genome build (Mar. 2006).
