# Supplementary material for: Preliminary evidence for association of genetic variants in pri-miR-34b/c and abnormal miR-34c expression with attention deficit and hyperactivity disorder
Source: Transl Psychiatry. 2016 Aug 30;6(8):e879–. doi: 10.1038/tp.2016.151 (PMC5022091; doi:10.1038/tp.2016.151)
Supplement: Supplementary Table 5 [file tp2016151x6.doc]

**Supplementary Table 5** Results from the *trans*-eQTL analyses using the MatrixEQTL R Package (P-value<0.05). A total of 681 genes were differentially expressed as a consequence of the presence of the rs4938723T risk variant in the pri-miR-34b/c promoter (P-value<0.05). A total of 292 genes were up-regulated and 389 were down-regulated.

| **Distant eQTLs:** | |  |  |  |  | **Distant eQTLs:** |  |  |  |  |
| --- | --- | --- | --- | --- | --- | --- | --- | --- | --- | --- |
| **DOWNREGULATED** | |  |  |  |  | **UPREGULATED** |  |  |  |  |
| **Gene** | **t-statistic** | **P-value** | **FDR** | **Beta** |  | **Gene** | **t-statistic** | **P-value** | **FDR** | **Beta** |
| *RHO* | -4.76 | 2.42e-05 | 0.34 | -0.10 |  | *SYT2* | 4.22 | 1.32e-04 | 0.78 | 0.12 |
| *OR6V1* | -4.64 | 3.49e-05 | 0.34 | -0.16 |  | *FAM177A1* | 4.02 | 2.45e-04 | 0.78 | 0.17 |
| *LOC554207* | -4.10 | 1.89e-04 | 0.78 | -0.11 |  | *ZNF552* | 3.82 | 4.45e-04 | 0.78 | 0.13 |
| *MARCH11* | -4.01 | 2.53e-04 | 0.78 | -0.15 |  | *PPFIBP2* | 3.68 | 6.80e-04 | 1 | 0.20 |
| *SKCG-1* | -3.97 | 2.87e-04 | 0.78 | -0.11 |  | *RAB28* | 3.54 | 1.00e-03 | 1 | 0.10 |
| *MeSP2* | -3.84 | 4.12e-04 | 0.78 | -0.12 |  | *MRPS17* | 3.52 | 1.06e-03 | 1 | 0.15 |
| *KSR2* | -3.84 | 4.15e-04 | 0.78 | -0.09 |  | *ZNF587* | 3.48 | 1.19e-03 | 1 | 0.10 |
| *BHMT2* | -3.83 | 4.31e-04 | 0.78 | -0.10 |  | *KRT35* | 3.44 | 1.37e-03 | 1 | 0.09 |
| *PRR23C* | -3.78 | 4.93e-04 | 0.79 | -0.08 |  | *WRNIP1* | 3.43 | 1.40e-03 | 1 | 0.08 |
| *RHPN1* | -3.59 | 8.85e-04 | 1 | -0.13 |  | *KLHDC10* | 3.41 | 1.46e-03 | 1 | 0.06 |
| *OTOA* | -3.54 | 1.00e-03 | 1 | -0.11 |  | *ARHGAP19* | 3.38 | 1.58e-03 | 1 | 0.09 |
| *ADAMTS12* | -3.47 | 1.25e-03 | 1 | -0.10 |  | *MIR30e* | 3.22 | 2.52e-03 | 1 | 0.19 |
| *LRFN2* | -3.46 | 1.26e-03 | 1 | -0.09 |  | *C17orf75* | 3.06 | 3.88e-03 | 1 | 0.13 |
| *DOCK3* | -3.46 | 1.29e-03 | 1 | -0.11 |  | *CROT* | 3.02 | 4.32e-03 | 1 | 0.12 |
| *ISL1* | -3.44 | 1.37e-03 | 1 | -0.08 |  | *GAPVD1* | 2.99 | 4.69e-03 | 1 | 0.05 |
| *PTOV1* | -3.40 | 1.49e-03 | 1 | -0.10 |  | *MFN1* | 2.97 | 5.00e-03 | 1 | 0.11 |
| *GRAMD1A* | -3.35 | 1.74e-03 | 1 | -0.11 |  | *SLC30A5* | 2.95 | 5.27e-03 | 1 | 0.10 |
| *GSTM5* | -3.33 | 1.83e-03 | 1 | -0.32 |  | *MCART2* | 2.93 | 5.47e-03 | 1 | 0.08 |
| *ACTA1* | -3.31 | 1.95e-03 | 1 | -0.11 |  | *CMYA5* | 2.92 | 5.68e-03 | 1 | 0.06 |
| *FLJ45244* | -3.29 | 2.04e-03 | 1 | -0.11 |  | *SeNP2* | 2.90 | 5.99e-03 | 1 | 0.10 |
| *DMRTA1* | -3.27 | 2.17e-03 | 1 | -0.09 |  | *SHFM1* | 2.90 | 6.05e-03 | 1 | 0.08 |
| *SNORA5C* | -3.26 | 2.23e-03 | 1 | -0.23 |  | *TeS* | 2.89 | 6.11e-03 | 1 | 0.10 |
| *ReLN* | -3.26 | 2.24e-03 | 1 | -0.07 |  | *MeD6* | 2.89 | 6.19e-03 | 1 | 0.07 |
| *C20orf46* | -3.25 | 2.28e-03 | 1 | -0.09 |  | *SMCR5* | 2.87 | 6.50e-03 | 1 | 0.09 |
| *FAM150A* | -3.23 | 2.43e-03 | 1 | -0.08 |  | *C6orf145* | 2.87 | 6.52e-03 | 1 | 0.16 |
| *TUBB2A* | -3.23 | 2.47e-03 | 1 | -0.16 |  | *ZNF18* | 2.85 | 6.89e-03 | 1 | 0.08 |
| *MeGF10* | -3.21 | 2.55e-03 | 1 | -0.06 |  | *KCNN2* | 2.77 | 8.39e-03 | 1 | 0.05 |
| *FLJ42418* | -3.18 | 2.79e-03 | 1 | -0.13 |  | *CCDC77* | 2.76 | 8.57e-03 | 1 | 0.10 |
| *OR1S2* | -3.17 | 2.88e-03 | 1 | -0.14 |  | *GPX7* | 2.76 | 8.65e-03 | 1 | 0.12 |
| *ATP13A1* | -3.16 | 2.99e-03 | 1 | -0.08 |  | *ZCCHC4* | 2.75 | 8.79e-03 | 1 | 0.11 |
| *ZHX3* | -3.15 | 3.07e-03 | 1 | -0.08 |  | *MIR29B1* | 2.74 | 8.97e-03 | 1 | 0.05 |
| *DHODH* | -3.10 | 3.50e-03 | 1 | -0.12 |  | *KPNA1* | 2.74 | 9.09e-03 | 1 | 0.08 |
| *HBe1* | -3.08 | 3.66e-03 | 1 | -0.15 |  | *CXCR6* | 2.73 | 9.26e-03 | 1 | 0.15 |
| *MS4A8B* | -3.07 | 3.81e-03 | 1 | -0.08 |  | *MTHFS* | 2.68 | 0.0105 | 1 | 0.12 |
| *PRG3* | -3.06 | 3.87e-03 | 1 | -0.10 |  | *eLP3* | 2.66 | 0.0112 | 1 | 0.07 |
| *MIR194-1* | -3.05 | 4.04e-03 | 1 | -0.08 |  | *PACRGL* | 2.65 | 0.0113 | 1 | 0.09 |
| *HTR2C* | -3.04 | 4.08e-03 | 1 | -0.08 |  | *COPS5* | 2.65 | 0.0113 | 1 | 0.10 |
| *eRVFRDe1* | -3.04 | 4.08e-03 | 1 | -0.08 |  | *C7orf45* | 2.64 | 0.0115 | 1 | 0.08 |
| *LAMP3* | -3.03 | 4.26e-03 | 1 | -0.15 |  | *UBA6* | 2.64 | 0.0117 | 1 | 0.06 |
| *ZNF865* | -3.01 | 4.48e-03 | 1 | -0.18 |  | *eRCC8* | 2.64 | 0.0118 | 1 | 0.13 |
| *RASD1* | -2.98 | 4.80e-03 | 1 | -0.10 |  | *UPF2* | 2.64 | 0.0118 | 1 | 0.05 |
| *C12orf55* | -2.98 | 4.86e-03 | 1 | -0.09 |  | *ALDH3A2* | 2.63 | 0.0120 | 1 | 0.06 |
| *ANO4* | -2.98 | 4.87e-03 | 1 | -0.07 |  | *ATG12* | 2.61 | 0.0125 | 1 | 0.09 |
| *C8orf73* | -2.94 | 5.36e-03 | 1 | -0.27 |  | *GPR183* | 2.61 | 0.0126 | 1 | 0.15 |
| *THY1* | -2.93 | 5.58e-03 | 1 | -0.09 |  | *SeTMAR* | 2.60 | 0.0130 | 1 | 0.10 |
| *PRAMeF10* | -2.92 | 5.67e-03 | 1 | -0.08 |  | *SLC25A2* | 2.59 | 0.0133 | 1 | 0.11 |
| *ROBO2* | -2.92 | 5.72e-03 | 1 | -0.09 |  | *KRT2* | 2.58 | 0.0134 | 1 | 0.07 |
| *C11orf40* | -2.91 | 5.79e-03 | 1 | -0.08 |  | *HS3ST3B1* | 2.58 | 0.0137 | 1 | 0.16 |
| *OR2D2* | -2.88 | 6.26e-03 | 1 | -0.13 |  | *MIR16-1* | 2.57 | 0.0139 | 1 | 0.05 |
| *CCDC11* | -2.88 | 6.29e-03 | 1 | -0.09 |  | *DIS3L* | 2.57 | 0.0139 | 1 | 0.09 |
| *KLKP1* | -2.88 | 6.32e-03 | 1 | -0.12 |  | *MIR95* | 2.57 | 0.0140 | 1 | 0.07 |
| *GSG1* | -2.86 | 6.69e-03 | 1 | -0.08 |  | *SCAMP1* | 2.56 | 0.0142 | 1 | 0.08 |
| *TINAG* | -2.85 | 6.85e-03 | 1 | -0.07 |  | *MAGT1* | 2.55 | 0.0145 | 1 | 0.08 |
| *eNGASe* | -2.83 | 7.25e-03 | 1 | -0.14 |  | *TMX2* | 2.55 | 0.0146 | 1 | 0.09 |
| *DIRC1* | -2.82 | 7.30e-03 | 1 | -0.11 |  | *KCTD9* | 2.55 | 0.0147 | 1 | 0.11 |
| *NAT2* | -2.82 | 7.31e-03 | 1 | -0.07 |  | *CRP* | 2.54 | 0.0149 | 1 | 0.05 |
| *CFB* | -2.82 | 7.35e-03 | 1 | -0.09 |  | *KIAA1279* | 2.54 | 0.0149 | 1 | 0.12 |
| *HSD3BP4* | -2.82 | 7.44e-03 | 1 | -0.15 |  | *UBXN4* | 2.54 | 0.0150 | 1 | 0.05 |
| *DCLK1* | -2.80 | 7.71e-03 | 1 | -0.08 |  | *CYP20A1* | 2.54 | 0.0150 | 1 | 0.09 |
| *ANKRD24* | -2.79 | 7.87e-03 | 1 | -0.12 |  | *C16orf72* | 2.54 | 0.0151 | 1 | 0.07 |
| *LMF2* | -2.79 | 7.94e-03 | 1 | -0.09 |  | *C1QL2* | 2.53 | 0.0153 | 1 | 0.07 |
| *ATXN7L1* | -2.79 | 8.02e-03 | 1 | -0.09 |  | *TXNDC15* | 2.53 | 0.0153 | 1 | 0.08 |
| *CCDC121* | -2.77 | 8.36e-03 | 1 | -0.12 |  | *AFTPH* | 2.53 | 0.0153 | 1 | 0.06 |
| *DGAT2L6* | -2.77 | 8.40e-03 | 1 | -0.06 |  | *WWC2* | 2.53 | 0.0154 | 1 | 0.12 |
| *FAM46D* | -2.77 | 8.43e-03 | 1 | -0.05 |  | *CCDC89* | 2.53 | 0.0155 | 1 | 0.09 |
| *CGA* | -2.77 | 8.47e-03 | 1 | -0.10 |  | *ZNF713* | 2.52 | 0.0156 | 1 | 0.10 |
| *ST5* | -2.76 | 8.50e-03 | 1 | -0.08 |  | *SMAD5* | 2.52 | 0.0156 | 1 | 0.14 |
| *SLC5A7* | -2.76 | 8.51e-03 | 1 | -0.07 |  | *CST9L* | 2.52 | 0.0156 | 1 | 0.07 |
| *eFCAB2* | -2.76 | 8.52e-03 | 1 | -0.12 |  | *C15orf58* | 2.52 | 0.0158 | 1 | 0.11 |
| *HeATR7A* | -2.75 | 8.85e-03 | 1 | -0.10 |  | *TMPRSS2* | 2.52 | 0.0159 | 1 | 0.05 |
| *KCNQ4* | -2.74 | 9.01e-03 | 1 | -0.09 |  | *TARS2* | 2.52 | 0.0159 | 1 | 0.07 |
| *CDH6* | -2.71 | 9.77e-03 | 1 | -0.09 |  | *OXGR1* | 2.51 | 0.0162 | 1 | 0.07 |
| *GLIS2* | -2.70 | 9.96e-03 | 1 | -0.09 |  | *CBR4* | 2.50 | 0.0164 | 1 | 0.10 |
| *C14orf178* | -2.70 | 0.0100 | 1 | -0.09 |  | *KLHL10* | 2.50 | 0.0165 | 1 | 0.04 |
| *MIR134* | -2.70 | 0.0101 | 1 | -0.15 |  | *ReXO4* | 2.49 | 0.0169 | 1 | 0.09 |
| *OR7e24* | -2.69 | 0.0103 | 1 | -0.09 |  | *ADH5* | 2.48 | 0.0173 | 1 | 0.10 |
| *NHLH1* | -2.68 | 0.0105 | 1 | -0.09 |  | *XPO5* | 2.47 | 0.0179 | 1 | 0.06 |
| *BCAS1* | -2.68 | 0.0106 | 1 | -0.09 |  | *ZNF45* | 2.46 | 0.0180 | 1 | 0.14 |
| *PTGeS2* | -2.68 | 0.0106 | 1 | -0.09 |  | *LRRC56* | 2.46 | 0.0180 | 1 | 0.10 |
| *CFB* | -2.68 | 0.0106 | 1 | -0.09 |  | *TXNDC9* | 2.46 | 0.0181 | 1 | 0.13 |
| *SH3GL3* | -2.67 | 0.0107 | 1 | -0.06 |  | *NCRNA00189* | 2.44 | 0.0190 | 1 | 0.25 |
| *SRRM4* | -2.67 | 0.0108 | 1 | -0.07 |  | *IReB2* | 2.44 | 0.0191 | 1 | 0.09 |
| *BSND* | -2.67 | 0.0109 | 1 | -0.07 |  | *GSTT1* | 2.43 | 0.0196 | 1 | 0.37 |
| *OR1D2* | -2.64 | 0.0116 | 1 | -0.08 |  | *ARL15* | 2.42 | 0.0198 | 1 | 0.09 |
| *PAX3* | -2.62 | 0.0122 | 1 | -0.06 |  | *TBCe* | 2.42 | 0.0199 | 1 | 0.06 |
| *GPRC5A* | -2.62 | 0.0122 | 1 | -0.07 |  | *SPIN2A* | 2.42 | 0.0200 | 1 | 0.09 |
| *RBMS1* | -2.61 | 0.0126 | 1 | -0.09 |  | *L3MBTL3* | 2.42 | 0.0201 | 1 | 0.10 |
| *LTA* | -2.61 | 0.0127 | 1 | -0.16 |  | *LONP1* | 2.42 | 0.0203 | 1 | 0.09 |
| *TAS2R40* | -2.60 | 0.0127 | 1 | -0.07 |  | *SNX24* | 2.41 | 0.0205 | 1 | 0.12 |
| *FHDC1* | -2.60 | 0.0128 | 1 | -0.07 |  | *TBCK* | 2.41 | 0.0206 | 1 | 0.07 |
| *PTGR1* | -2.60 | 0.0129 | 1 | -0.06 |  | *C20orf72* | 2.40 | 0.0210 | 1 | 0.14 |
| *LHX8* | -2.60 | 0.0129 | 1 | -0.07 |  | *CABP1* | 2.39 | 0.0214 | 1 | 0.06 |
| *PLA2G1B* | -2.60 | 0.0130 | 1 | -0.09 |  | *ZNF410* | 2.39 | 0.0215 | 1 | 0.09 |
| *ZNF609* | -2.60 | 0.0130 | 1 | -0.08 |  | *SPATS2* | 2.39 | 0.0216 | 1 | 0.12 |
| *FAM181B* | -2.59 | 0.0132 | 1 | -0.10 |  | *AAGAB* | 2.39 | 0.0216 | 1 | 0.09 |
| *OR6S1* | -2.59 | 0.0132 | 1 | -0.07 |  | *XIAP* | 2.39 | 0.0217 | 1 | 0.09 |
| *DLX2* | -2.59 | 0.0132 | 1 | -0.09 |  | *KDM5B* | 2.38 | 0.0218 | 1 | 0.06 |
| *DGKZ* | -2.58 | 0.0136 | 1 | -0.12 |  | *PRKY* | 2.38 | 0.0220 | 1 | 0.10 |
| *TMeM179* | -2.58 | 0.0137 | 1 | -0.09 |  | *MAGeB2* | 2.38 | 0.0222 | 1 | 0.06 |
| *LOC100190938* | -2.57 | 0.0138 | 1 | -0.07 |  | *TMX3* | 2.38 | 0.0223 | 1 | 0.08 |
| *HOXD12* | -2.57 | 0.0139 | 1 | -0.07 |  | *MRPL39* | 2.37 | 0.0223 | 1 | 0.08 |
| *CPNe6* | -2.57 | 0.0140 | 1 | -0.06 |  | *GKN1* | 2.37 | 0.0226 | 1 | 0.05 |
| *HHLA2* | -2.56 | 0.0141 | 1 | -0.08 |  | *TRIP4* | 2.36 | 0.0229 | 1 | 0.09 |
| *ePS8L3* | -2.56 | 0.0142 | 1 | -0.07 |  | *ADAT1* | 2.36 | 0.0231 | 1 | 0.08 |
| *FLJ33996* | -2.56 | 0.0143 | 1 | -0.09 |  | *RPS4X* | 2.36 | 0.0231 | 1 | 0.06 |
| *RIMS2* | -2.56 | 0.0144 | 1 | -0.08 |  | *BDNF* | 2.36 | 0.0233 | 1 | 0.07 |
| *C3orf56* | -2.55 | 0.0145 | 1 | -0.06 |  | *ANKRD10* | 2.36 | 0.0233 | 1 | 0.05 |
| *PIP5KL1* | -2.55 | 0.0146 | 1 | -0.09 |  | *PAIP1* | 2.36 | 0.0234 | 1 | 0.15 |
| *ATHL1* | -2.55 | 0.0147 | 1 | -0.25 |  | *XKR6* | 2.35 | 0.0234 | 1 | 0.08 |
| *PRAMeF10* | -2.54 | 0.0150 | 1 | -0.06 |  | *TMeM187* | 2.35 | 0.0235 | 1 | 0.08 |
| *FAM184A* | -2.53 | 0.0153 | 1 | -0.09 |  | *NBPF7* | 2.35 | 0.0236 | 1 | 0.06 |
| *LIN28A* | -2.53 | 0.0155 | 1 | -0.11 |  | *DHX57* | 2.35 | 0.0237 | 1 | 0.07 |
| *KCNIP3* | -2.53 | 0.0155 | 1 | -0.09 |  | *OLFML3* | 2.35 | 0.0238 | 1 | 0.07 |
| *MLL4* | -2.52 | 0.0156 | 1 | -0.09 |  | *LOC729020* | 2.35 | 0.0239 | 1 | 0.11 |
| *eBF2* | -2.52 | 0.0159 | 1 | -0.07 |  | *ACSL3* | 2.34 | 0.0241 | 1 | 0.08 |
| *SIAH3* | -2.51 | 0.0160 | 1 | -0.09 |  | *C2orf49* | 2.34 | 0.0243 | 1 | 0.08 |
| *CST11* | -2.50 | 0.0164 | 1 | -0.07 |  | *C3orf57* | 2.34 | 0.0244 | 1 | 0.17 |
| *MTNR1B* | -2.50 | 0.0166 | 1 | -0.08 |  | *FBXO11* | 2.34 | 0.0244 | 1 | 0.06 |
| *GSTM1* | -2.50 | 0.0167 | 1 | -0.61 |  | *NAPB* | 2.33 | 0.0245 | 1 | 0.11 |
| *eSRP2* | -2.50 | 0.0167 | 1 | -0.09 |  | *PIH1D2* | 2.33 | 0.0248 | 1 | 0.04 |
| *CHIA* | -2.49 | 0.0168 | 1 | -0.06 |  | *RARS2* | 2.33 | 0.0249 | 1 | 0.08 |
| *GLDN* | -2.49 | 0.0169 | 1 | -0.06 |  | *CIB4* | 2.33 | 0.0250 | 1 | 0.06 |
| *SMC1B* | -2.49 | 0.0169 | 1 | -0.07 |  | *KLKB1* | 2.32 | 0.0251 | 1 | 0.16 |
| *HUNK* | -2.49 | 0.0171 | 1 | -0.08 |  | *CeP192* | 2.32 | 0.0252 | 1 | 0.07 |
| *TAS2R42* | -2.48 | 0.0173 | 1 | -0.11 |  | *KIDINS220* | 2.32 | 0.0252 | 1 | 0.04 |
| *CAMK1G* | -2.48 | 0.0174 | 1 | -0.06 |  | *DHTKD1* | 2.32 | 0.0254 | 1 | 0.08 |
| *NCRNA00277* | -2.47 | 0.0177 | 1 | -0.06 |  | *ZC3H10* | 2.32 | 0.0256 | 1 | 0.07 |
| *FAM75D5* | -2.47 | 0.0177 | 1 | -0.09 |  | *CDK19* | 2.31 | 0.0257 | 1 | 0.07 |
| *HAGHL* | -2.47 | 0.0177 | 1 | -0.10 |  | *ROD1* | 2.31 | 0.0258 | 1 | 0.07 |
| *OIT3* | -2.47 | 0.0177 | 1 | -0.06 |  | *C14orf179* | 2.31 | 0.0262 | 1 | 0.07 |
| *ADAMTS9* | -2.47 | 0.0178 | 1 | -0.06 |  | *C9orf3* | 2.31 | 0.0263 | 1 | 0.08 |
| *MIR219-1* | -2.47 | 0.0179 | 1 | -0.08 |  | *eFHA1* | 2.30 | 0.0264 | 1 | 0.08 |
| *LOC220077* | -2.46 | 0.0180 | 1 | -0.08 |  | *OR7G2* | 2.30 | 0.0265 | 1 | 0.06 |
| *LOC100507404* | -2.44 | 0.0189 | 1 | -0.08 |  | *C4orf45* | 2.30 | 0.0267 | 1 | 0.04 |
| *ZFHX2* | -2.44 | 0.0192 | 1 | -0.09 |  | *SORCS3* | 2.30 | 0.0268 | 1 | 0.16 |
| *LOC154872* | -2.43 | 0.0195 | 1 | -0.06 |  | *C2orf43* | 2.29 | 0.0270 | 1 | 0.09 |
| *ARHGAP4* | -2.43 | 0.0196 | 1 | -0.06 |  | *ZNF433* | 2.29 | 0.0271 | 1 | 0.05 |
| *RGS4* | -2.42 | 0.0200 | 1 | -0.06 |  | *TNPO3* | 2.29 | 0.0272 | 1 | 0.06 |
| *DPYSL4* | -2.42 | 0.0201 | 1 | -0.15 |  | *USP33* | 2.29 | 0.0273 | 1 | 0.06 |
| *GDNF* | -2.42 | 0.0202 | 1 | -0.06 |  | *SeRPINI1* | 2.28 | 0.0278 | 1 | 0.10 |
| *MMP20* | -2.42 | 0.0203 | 1 | -0.05 |  | *KLRC1* | 2.28 | 0.0279 | 1 | 0.29 |
| *FBXL19* | -2.41 | 0.0204 | 1 | -0.08 |  | *ZNF560* | 2.28 | 0.0280 | 1 | 0.07 |
| *RAPGeF3* | -2.41 | 0.0204 | 1 | -0.11 |  | *SLC24A1* | 2.28 | 0.0281 | 1 | 0.08 |
| *SH3RF3* | -2.41 | 0.0205 | 1 | -0.12 |  | *BPNT1* | 2.27 | 0.0286 | 1 | 0.10 |
| *AQPeP* | -2.41 | 0.0205 | 1 | -0.06 |  | *RCSD1* | 2.27 | 0.0288 | 1 | 0.07 |
| *GPRC6A* | -2.41 | 0.0206 | 1 | -0.09 |  | *ANKAR* | 2.26 | 0.0291 | 1 | 0.09 |
| *TFF3* | -2.41 | 0.0206 | 1 | -0.07 |  | *DYNC2H1* | 2.26 | 0.0292 | 1 | 0.06 |
| *CCDC46* | -2.41 | 0.0207 | 1 | -0.09 |  | *OSTF1* | 2.26 | 0.0294 | 1 | 0.07 |
| *OR51e1* | -2.41 | 0.0207 | 1 | -0.07 |  | *NFYB* | 2.26 | 0.0294 | 1 | 0.05 |
| *OSCP1* | -2.40 | 0.0208 | 1 | -0.07 |  | *ITPA* | 2.26 | 0.0294 | 1 | 0.08 |
| *C16orf85* | -2.40 | 0.0210 | 1 | -0.07 |  | *C17orf85* | 2.26 | 0.0295 | 1 | 0.04 |
| *PPIL5* | -2.40 | 0.0210 | 1 | -0.09 |  | *eXOG* | 2.25 | 0.0301 | 1 | 0.09 |
| *HS3ST3A1* | -2.40 | 0.0211 | 1 | -0.10 |  | *LCMT1* | 2.24 | 0.0303 | 1 | 0.05 |
| *LOC401286* | -2.40 | 0.0211 | 1 | -0.06 |  | *BCAP29* | 2.24 | 0.0307 | 1 | 0.07 |
| *SeTD1A* | -2.40 | 0.0211 | 1 | -0.06 |  | *ZNF300* | 2.23 | 0.0311 | 1 | 0.11 |
| *DGKD* | -2.39 | 0.0215 | 1 | -0.07 |  | *MeTTL10* | 2.23 | 0.0313 | 1 | 0.07 |
| *C6orf54* | -2.39 | 0.0215 | 1 | -0.08 |  | *ARL3* | 2.23 | 0.0314 | 1 | 0.10 |
| *KIAA1409* | -2.39 | 0.0217 | 1 | -0.05 |  | *RPL22* | 2.23 | 0.0315 | 1 | 0.12 |
| *DeFB115* | -2.38 | 0.0221 | 1 | -0.07 |  | *CCR5* | 2.23 | 0.0316 | 1 | 0.08 |
| *LHPP* | -2.38 | 0.0221 | 1 | -0.10 |  | *MeTTL5* | 2.23 | 0.0316 | 1 | 0.07 |
| *KRTAP4-2* | -2.38 | 0.0222 | 1 | -0.10 |  | *FAM19A1* | 2.22 | 0.0318 | 1 | 0.15 |
| *FGF14* | -2.38 | 0.0223 | 1 | -0.05 |  | *HIST3H2A* | 2.22 | 0.0322 | 1 | 0.07 |
| *RASAL1* | -2.37 | 0.0223 | 1 | -0.07 |  | *TTLL13* | 2.21 | 0.0326 | 1 | 0.08 |
| *CYP2B6* | -2.37 | 0.0224 | 1 | -0.07 |  | *MYO1D* | 2.21 | 0.0327 | 1 | 0.15 |
| *C19orf6* | -2.37 | 0.0225 | 1 | -0.05 |  | *KCTD10* | 2.21 | 0.0328 | 1 | 0.09 |
| *FAM168A* | -2.37 | 0.0225 | 1 | -0.08 |  | *SLC22A7* | 2.21 | 0.0329 | 1 | 0.05 |
| *AGRN* | -2.36 | 0.0229 | 1 | -0.08 |  | *XPNPeP2* | 2.20 | 0.0333 | 1 | 0.07 |
| *LOC401410* | -2.36 | 0.0230 | 1 | -0.08 |  | *AKR1C2* | 2.20 | 0.0335 | 1 | 0.14 |
| *KLF16* | -2.36 | 0.0230 | 1 | -0.08 |  | *ZBTB8OS* | 2.20 | 0.0336 | 1 | 0.07 |
| *C20orf173* | -2.36 | 0.0232 | 1 | -0.08 |  | *MLH1* | 2.20 | 0.0338 | 1 | 0.07 |
| *LOC257358* | -2.36 | 0.0232 | 1 | -0.08 |  | *GeMIN8P4* | 2.19 | 0.0340 | 1 | 0.07 |
| *GPSM3* | -2.35 | 0.0236 | 1 | -0.09 |  | *LONRF1* | 2.19 | 0.0342 | 1 | 0.10 |
| *FAM184B* | -2.35 | 0.0238 | 1 | -0.08 |  | *POM121L12* | 2.19 | 0.0342 | 1 | 0.07 |
| *MYOG* | -2.34 | 0.0240 | 1 | -0.09 |  | *GNRH1* | 2.19 | 0.0343 | 1 | 0.09 |
| *LLPH* | -2.34 | 0.0240 | 1 | -0.14 |  | *CCT8* | 2.19 | 0.0346 | 1 | 0.07 |
| *SNCAIP* | -2.34 | 0.0242 | 1 | -0.08 |  | *WTAP* | 2.18 | 0.0349 | 1 | 0.05 |
| *TUB* | -2.34 | 0.0243 | 1 | -0.08 |  | *SAAL1* | 2.18 | 0.0349 | 1 | 0.08 |
| *HYAL1* | -2.33 | 0.0246 | 1 | -0.07 |  | *KBTBD6* | 2.18 | 0.0350 | 1 | 0.09 |
| *FGF10* | -2.33 | 0.0247 | 1 | -0.07 |  | *ABHD3* | 2.18 | 0.0351 | 1 | 0.07 |
| *COL22A1* | -2.33 | 0.0248 | 1 | -0.06 |  | *BTBD7* | 2.18 | 0.0353 | 1 | 0.06 |
| *IFI44L* | -2.33 | 0.0248 | 1 | -0.35 |  | *DeFB135* | 2.18 | 0.0354 | 1 | 0.04 |
| *KHSRP* | -2.32 | 0.0252 | 1 | -0.08 |  | *HIST1H4B* | 2.17 | 0.0356 | 1 | 0.16 |
| *ZNF496* | -2.32 | 0.0256 | 1 | -0.10 |  | *TRIM38* | 2.17 | 0.0356 | 1 | 0.07 |
| *RNU11* | -2.32 | 0.0256 | 1 | -0.29 |  | *SNX13* | 2.17 | 0.0357 | 1 | 0.07 |
| *CITeD4* | -2.31 | 0.0258 | 1 | -0.14 |  | *BMPR1A* | 2.17 | 0.0357 | 1 | 0.14 |
| *C19orf18* | -2.31 | 0.0258 | 1 | -0.07 |  | *SeTD8* | 2.17 | 0.0358 | 1 | 0.09 |
| *CCDC103* | -2.31 | 0.0258 | 1 | -0.06 |  | *KLHDC10* | 2.17 | 0.0358 | 1 | 0.07 |
| *LOC100132319* | -2.31 | 0.0259 | 1 | -0.10 |  | *USP39* | 2.17 | 0.0359 | 1 | 0.06 |
| *KLF17* | -2.31 | 0.0259 | 1 | -0.06 |  | *GTF3C3* | 2.16 | 0.0365 | 1 | 0.08 |
| *TNK2* | -2.31 | 0.0260 | 1 | -0.11 |  | *GXYLT1* | 2.16 | 0.0367 | 1 | 0.12 |
| *RAB19* | -2.31 | 0.0263 | 1 | -0.08 |  | *TTC12* | 2.16 | 0.0368 | 1 | 0.12 |
| *STRC* | -2.30 | 0.0264 | 1 | -0.08 |  | *UBe2N* | 2.15 | 0.0372 | 1 | 0.08 |
| *SLC2A12* | -2.30 | 0.0265 | 1 | -0.05 |  | *ZFP42* | 2.15 | 0.0373 | 1 | 0.06 |
| *OR4Q2* | -2.30 | 0.0265 | 1 | -0.10 |  | *OXCT1* | 2.15 | 0.0373 | 1 | 0.09 |
| *BANF2* | -2.30 | 0.0266 | 1 | -0.06 |  | *eNTPD7* | 2.15 | 0.0376 | 1 | 0.09 |
| *PAGe3* | -2.30 | 0.0267 | 1 | -0.06 |  | *CCDC28A* | 2.15 | 0.0377 | 1 | 0.08 |
| *FBRS* | -2.30 | 0.0268 | 1 | -0.10 |  | *ZNF280C* | 2.15 | 0.0378 | 1 | 0.07 |
| *SCGB2A1* | -2.30 | 0.0268 | 1 | -0.08 |  | *GNB2L1* | 2.15 | 0.0378 | 1 | 0.03 |
| *NOTCH3* | -2.30 | 0.0269 | 1 | -0.07 |  | *LRRC23* | 2.15 | 0.0379 | 1 | 0.10 |
| *NCLN* | -2.29 | 0.0269 | 1 | -0.06 |  | *TBC1D30* | 2.14 | 0.0380 | 1 | 0.07 |
| *C8B* | -2.29 | 0.0270 | 1 | -0.05 |  | *TLR3* | 2.14 | 0.0381 | 1 | 0.14 |
| *NBLA00301* | -2.29 | 0.0275 | 1 | -0.07 |  | *BAG5* | 2.14 | 0.0385 | 1 | 0.09 |
| *LOC100128988* | -2.29 | 0.0275 | 1 | -0.06 |  | *ACAD9* | 2.14 | 0.0385 | 1 | 0.09 |
| *ABCC1* | -2.28 | 0.0277 | 1 | -0.07 |  | *SUCLA2* | 2.14 | 0.0385 | 1 | 0.09 |
| *ANAPC2* | -2.28 | 0.0278 | 1 | -0.07 |  | *KLHL9* | 2.14 | 0.0386 | 1 | 0.10 |
| *OR10A3* | -2.28 | 0.0279 | 1 | -0.06 |  | *TUBA1B* | 2.14 | 0.0386 | 1 | 0.06 |
| *TRIM9* | -2.28 | 0.0279 | 1 | -0.05 |  | *INPP1* | 2.13 | 0.0388 | 1 | 0.13 |
| *MMP7* | -2.28 | 0.0280 | 1 | -0.06 |  | *ZKSCAN3* | 2.13 | 0.0389 | 1 | 0.11 |
| *RBFOX1* | -2.28 | 0.0280 | 1 | -0.06 |  | *PCCA* | 2.13 | 0.0390 | 1 | 0.10 |
| *LPPR1* | -2.28 | 0.0282 | 1 | -0.08 |  | *ATL2* | 2.13 | 0.0392 | 1 | 0.07 |
| *PSAPL1* | -2.27 | 0.0283 | 1 | -0.08 |  | *UNC50* | 2.13 | 0.0394 | 1 | 0.08 |
| *CPB1* | -2.27 | 0.0283 | 1 | -0.06 |  | *HIGD2B* | 2.13 | 0.0395 | 1 | 0.05 |
| *GPR110* | -2.27 | 0.0284 | 1 | -0.07 |  | *C20orf7* | 2.13 | 0.0395 | 1 | 0.07 |
| *IYD* | -2.27 | 0.0288 | 1 | -0.06 |  | *FRG1* | 2.13 | 0.0396 | 1 | 0.09 |
| *NDUFA4L2* | -2.26 | 0.0289 | 1 | -0.09 |  | *CCDC82* | 2.12 | 0.0399 | 1 | 0.10 |
| *SPRR1B* | -2.26 | 0.0290 | 1 | -0.06 |  | *VPS36* | 2.12 | 0.0400 | 1 | 0.06 |
| *IFNB1* | -2.26 | 0.0291 | 1 | -0.06 |  | *MORN4* | 2.12 | 0.0401 | 1 | 0.07 |
| *PTPRT* | -2.25 | 0.0296 | 1 | -0.05 |  | *SYTL2* | 2.11 | 0.0409 | 1 | 0.17 |
| *SORBS3* | -2.25 | 0.0296 | 1 | -0.11 |  | *CTAGe3P* | 2.11 | 0.0414 | 1 | 0.06 |
| *FAM120AOS* | -2.25 | 0.0296 | 1 | -0.06 |  | *eSCO1* | 2.10 | 0.0415 | 1 | 0.08 |
| *HCRP1* | -2.25 | 0.0298 | 1 | -0.09 |  | *PTPN11* | 2.10 | 0.0415 | 1 | 0.08 |
| *C21orf2* | -2.25 | 0.0301 | 1 | -0.10 |  | *LPA* | 2.10 | 0.0416 | 1 | 0.08 |
| *AGBL4* | -2.25 | 0.0302 | 1 | -0.07 |  | *TMeM48* | 2.10 | 0.0418 | 1 | 0.07 |
| *KRTAP10-2* | -2.24 | 0.0302 | 1 | -0.11 |  | *eIF4A3* | 2.10 | 0.0419 | 1 | 0.08 |
| *OR5L1* | -2.24 | 0.0303 | 1 | -0.08 |  | *LMAN1* | 2.10 | 0.0421 | 1 | 0.09 |
| *OR8H1* | -2.24 | 0.0304 | 1 | -0.10 |  | *ReTSAT* | 2.10 | 0.0422 | 1 | 0.06 |
| *APOA1* | -2.24 | 0.0306 | 1 | -0.06 |  | *ZDHHC4* | 2.10 | 0.0423 | 1 | 0.06 |
| *G6PC* | -2.23 | 0.0310 | 1 | -0.05 |  | *CD59* | 2.10 | 0.0423 | 1 | 0.12 |
| *MeGF6* | -2.23 | 0.0310 | 1 | -0.12 |  | *eFCAB2* | 2.10 | 0.0423 | 1 | 0.10 |
| *LOC100131756* | -2.23 | 0.0311 | 1 | -0.07 |  | *NMe6* | 2.10 | 0.0423 | 1 | 0.08 |
| *NISCH* | -2.23 | 0.0313 | 1 | -0.06 |  | *ACP2* | 2.10 | 0.0424 | 1 | 0.09 |
| *ARSH* | -2.23 | 0.0313 | 1 | -0.08 |  | *KIAA0368* | 2.09 | 0.0424 | 1 | 0.05 |
| *KCNQ1DN* | -2.23 | 0.0313 | 1 | -0.11 |  | *CCDC42* | 2.09 | 0.0425 | 1 | 0.07 |
| *ROBO4* | -2.23 | 0.0315 | 1 | -0.07 |  | *BeND6* | 2.09 | 0.0428 | 1 | 0.05 |
| *LOC391766* | -2.23 | 0.0315 | 1 | -0.24 |  | *TRMT112* | 2.09 | 0.0430 | 1 | 0.07 |
| *SeTD1B* | -2.23 | 0.0316 | 1 | -0.08 |  | *ZDHHC2* | 2.09 | 0.0431 | 1 | 0.07 |
| *F2* | -2.22 | 0.0317 | 1 | -0.05 |  | *PUS10* | 2.09 | 0.0432 | 1 | 0.12 |
| *FAM13A* | -2.22 | 0.0317 | 1 | -0.14 |  | *PGM3* | 2.09 | 0.0432 | 1 | 0.08 |
| *NLRP9* | -2.22 | 0.0317 | 1 | -0.06 |  | *ANKRD13C* | 2.08 | 0.0434 | 1 | 0.05 |
| *ATXN7L1* | -2.22 | 0.0318 | 1 | -0.07 |  | *INTS7* | 2.08 | 0.0435 | 1 | 0.10 |
| *IFFO1* | -2.22 | 0.0319 | 1 | -0.10 |  | *MIR96* | 2.08 | 0.0438 | 1 | 0.06 |
| *POM121L2* | -2.22 | 0.0319 | 1 | -0.06 |  | *CCDC53* | 2.08 | 0.0438 | 1 | 0.08 |
| *eNTPD3* | -2.22 | 0.0321 | 1 | -0.08 |  | *RCHY1* | 2.08 | 0.0439 | 1 | 0.15 |
| *FAM172B* | -2.22 | 0.0321 | 1 | -0.12 |  | *XPO1* | 2.08 | 0.0441 | 1 | 0.07 |
| *BeT3L* | -2.21 | 0.0324 | 1 | -0.09 |  | *MRPS33* | 2.08 | 0.0441 | 1 | 0.10 |
| *DAZL* | -2.21 | 0.0325 | 1 | -0.09 |  | *SPATA5L1* | 2.08 | 0.0442 | 1 | 0.12 |
| *UNC80* | -2.21 | 0.0325 | 1 | -0.05 |  | *AKR7L* | 2.08 | 0.0442 | 1 | 0.07 |
| *FABP4* | -2.21 | 0.0326 | 1 | -0.07 |  | *LOC400940* | 2.08 | 0.0442 | 1 | 0.05 |
| *ALOX12B* | -2.21 | 0.0326 | 1 | -0.06 |  | *PPP2R1B* | 2.07 | 0.0443 | 1 | 0.07 |
| *RHBDL1* | -2.21 | 0.0327 | 1 | -0.09 |  | *PSMD13* | 2.07 | 0.0446 | 1 | 0.09 |
| *PSORS1C1* | -2.21 | 0.0329 | 1 | -0.06 |  | *TTTY11* | 2.07 | 0.0446 | 1 | 0.06 |
| *C2CD4A* | -2.21 | 0.0330 | 1 | -0.09 |  | *CCDC30* | 2.07 | 0.0447 | 1 | 0.07 |
| *FLJ46363* | -2.21 | 0.0331 | 1 | -0.08 |  | *HOXA3* | 2.07 | 0.0447 | 1 | 0.07 |
| *SATL1* | -2.20 | 0.0333 | 1 | -0.08 |  | *ANXA7* | 2.07 | 0.0449 | 1 | 0.06 |
| *ATRNL1* | -2.20 | 0.0333 | 1 | -0.08 |  | *PSMA3* | 2.07 | 0.0451 | 1 | 0.08 |
| *GYS2* | -2.20 | 0.0334 | 1 | -0.06 |  | *COMMD1* | 2.06 | 0.0453 | 1 | 0.06 |
| *CDH7* | -2.20 | 0.0335 | 1 | -0.06 |  | *C4orf43* | 2.06 | 0.0454 | 1 | 0.11 |
| *CYP3A5* | -2.20 | 0.0335 | 1 | -0.05 |  | *PAPSS1* | 2.06 | 0.0454 | 1 | 0.09 |
| *DDX4* | -2.20 | 0.0335 | 1 | -0.06 |  | *ZNF461* | 2.06 | 0.0455 | 1 | 0.08 |
| *TMeM178* | -2.20 | 0.0336 | 1 | -0.08 |  | *C6orf35* | 2.06 | 0.0455 | 1 | 0.10 |
| *MeD26* | -2.20 | 0.0336 | 1 | -0.06 |  | *LZTFL1* | 2.06 | 0.0456 | 1 | 0.08 |
| *SCN8A* | -2.19 | 0.0339 | 1 | -0.08 |  | *ZNF611* | 2.06 | 0.0456 | 1 | 0.09 |
| *ANKRD53* | -2.19 | 0.0341 | 1 | -0.07 |  | *IQCG* | 2.06 | 0.0456 | 1 | 0.07 |
| *DDAH1* | -2.19 | 0.0342 | 1 | -0.09 |  | *FMR1* | 2.06 | 0.0461 | 1 | 0.06 |
| *KRTAP24-1* | -2.19 | 0.0344 | 1 | -0.07 |  | *C6orf52* | 2.06 | 0.0462 | 1 | 0.05 |
| *FAM47C* | -2.19 | 0.0344 | 1 | -0.07 |  | *TRPC5* | 2.06 | 0.0462 | 1 | 0.05 |
| *CFI* | -2.19 | 0.0346 | 1 | -0.04 |  | *NAIF1* | 2.06 | 0.0462 | 1 | 0.08 |
| *WNT10A* | -2.19 | 0.0346 | 1 | -0.10 |  | *KDeLR2* | 2.06 | 0.0463 | 1 | 0.08 |
| *BLCAP* | -2.18 | 0.0348 | 1 | -0.07 |  | *UBB* | 2.05 | 0.0463 | 1 | 0.05 |
| *KIAA0195* | -2.18 | 0.0348 | 1 | -0.06 |  | *NBeAL1* | 2.05 | 0.0464 | 1 | 0.09 |
| *TRPM8* | -2.18 | 0.0349 | 1 | -0.04 |  | *MeG3* | 2.05 | 0.0465 | 1 | 0.13 |
| *TMIGD2* | -2.18 | 0.0351 | 1 | -0.09 |  | *PLeKHG7* | 2.05 | 0.0465 | 1 | 0.10 |
| *OR52e4* | -2.18 | 0.0352 | 1 | -0.11 |  | *IRAK2* | 2.05 | 0.0467 | 1 | 0.08 |
| *CXorf61* | -2.18 | 0.0352 | 1 | -0.10 |  | *SPOP* | 2.05 | 0.0469 | 1 | 0.06 |
| *CFB* | -2.18 | 0.0352 | 1 | -0.09 |  | *TPH1* | 2.05 | 0.0470 | 1 | 0.12 |
| *ZNF726* | -2.18 | 0.0353 | 1 | -0.06 |  | *HMGCL* | 2.05 | 0.0472 | 1 | 0.09 |
| *ACSS3* | -2.18 | 0.0353 | 1 | -0.08 |  | *PPIL4* | 2.05 | 0.0472 | 1 | 0.06 |
| *ATXN7L1* | -2.18 | 0.0353 | 1 | -0.08 |  | *GDAP2* | 2.05 | 0.0473 | 1 | 0.06 |
| *FAM84A* | -2.17 | 0.0355 | 1 | -0.07 |  | *ITGA3* | 2.04 | 0.0475 | 1 | 0.08 |
| *ARAF2P* | -2.17 | 0.0355 | 1 | -0.12 |  | *FLJ42875* | 2.04 | 0.0478 | 1 | 0.09 |
| *PDYN* | -2.17 | 0.0355 | 1 | -0.06 |  | *NASP* | 2.04 | 0.0479 | 1 | 0.09 |
| *AGXT2* | -2.17 | 0.0361 | 1 | -0.07 |  | *IFT52* | 2.04 | 0.0482 | 1 | 0.06 |
| *MYCBPAP* | -2.17 | 0.0362 | 1 | -0.07 |  | *MARCH5* | 2.03 | 0.0484 | 1 | 0.09 |
| *UNC5C* | -2.16 | 0.0367 | 1 | -0.05 |  | *MAGeC3* | 2.03 | 0.0485 | 1 | 0.05 |
| *APOA1* | -2.16 | 0.0367 | 1 | -0.06 |  | *TMeM65* | 2.03 | 0.0487 | 1 | 0.06 |
| *YOD1* | -2.16 | 0.0368 | 1 | -0.08 |  | *MeD22* | 2.03 | 0.0488 | 1 | 0.09 |
| *PCDHB8* | -2.16 | 0.0368 | 1 | -0.06 |  | *ABI1* | 2.03 | 0.0489 | 1 | 0.05 |
| *MIR208A* | -2.16 | 0.0368 | 1 | -0.05 |  | *TRIM52* | 2.03 | 0.0492 | 1 | 0.09 |
| *GPR87* | -2.15 | 0.0375 | 1 | -0.04 |  | *SNX16* | 2.03 | 0.0492 | 1 | 0.11 |
| *MOGAT2* | -2.15 | 0.0377 | 1 | -0.06 |  | *ZNF224* | 2.02 | 0.0495 | 1 | 0.07 |
| *MID1* | -2.15 | 0.0379 | 1 | -0.04 |  | *PeX5* | 2.02 | 0.0496 | 1 | 0.08 |
| *VAMP2* | -2.14 | 0.0379 | 1 | -0.07 |  | *SULT2A1* | 2.02 | 0.0499 | 1 | 0.04 |
| *SMAP2* | -2.14 | 0.0386 | 1 | -0.08 |  |  |  | | | |
| *NKAIN3* | -2.14 | 0.0387 | 1 | -0.07 |  |  |  | | | |
| *DHX37* | -2.14 | 0.0387 | 1 | -0.06 |  |  |  | | | |
| *eSPNP* | -2.13 | 0.0390 | 1 | -0.13 |  |  |  | | | |
| *PARP6* | -2.13 | 0.0392 | 1 | -0.05 |  |  |  | | | |
| *MeOX1* | -2.13 | 0.0393 | 1 | -0.08 |  |  |  | | | |
| *GALNT13* | -2.13 | 0.0394 | 1 | -0.06 |  |  |  | | | |
| *CRLF1* | -2.13 | 0.0395 | 1 | -0.08 |  |  |  | | | |
| *SPNS2* | -2.13 | 0.0395 | 1 | -0.07 |  |  |  | | | |
| *UAP1L1* | -2.13 | 0.0396 | 1 | -0.14 |  |  |  | | | |
| *GDF9* | -2.13 | 0.0396 | 1 | -0.06 |  |  |  | | | |
| *OR4C13* | -2.12 | 0.0397 | 1 | -0.16 |  |  |  | | | |
| *GPR101* | -2.12 | 0.0400 | 1 | -0.07 |  |  |  | | | |
| *TPH2* | -2.12 | 0.0400 | 1 | -0.05 |  |  |  | | | |
| *NT5C1B* | -2.12 | 0.0401 | 1 | -0.06 |  |  |  | | | |
| *MX1* | -2.12 | 0.0402 | 1 | -0.20 |  |  |  | | | |
| *LTA* | -2.12 | 0.0403 | 1 | -0.12 |  |  |  | | | |
| *SLC9A11* | -2.12 | 0.0403 | 1 | -0.05 |  |  |  | | | |
| *FLJ42220* | -2.12 | 0.0404 | 1 | -0.05 |  |  |  | | | |
| *C17orf68* | -2.12 | 0.0404 | 1 | -0.11 |  |  |  | | | |
| *eML3* | -2.12 | 0.0405 | 1 | -0.07 |  |  |  | | | |
| *FOXC1* | -2.12 | 0.0405 | 1 | -0.07 |  |  |  | | | |
| *DPYSL5* | -2.11 | 0.0406 | 1 | -0.06 |  |  |  | | | |
| *DGKQ* | -2.11 | 0.0408 | 1 | -0.12 |  |  |  | | | |
| *WIZ* | -2.11 | 0.0409 | 1 | -0.09 |  |  |  | | | |
| *PLCH2* | -2.11 | 0.0409 | 1 | -0.10 |  |  |  | | | |
| *DBP* | -2.11 | 0.0409 | 1 | -0.08 |  |  |  | | | |
| *SLIT2* | -2.11 | 0.0410 | 1 | -0.05 |  |  |  | | | |
| *FAM59A* | -2.11 | 0.0410 | 1 | -0.05 |  |  |  | | | |
| *SLC14A2* | -2.11 | 0.0410 | 1 | -0.07 |  |  |  | | | |
| *FLJ34503* | -2.11 | 0.0411 | 1 | -0.06 |  |  |  | | | |
| *KLK6* | -2.11 | 0.0411 | 1 | -0.05 |  |  |  | | | |
| *PCBP3* | -2.10 | 0.0417 | 1 | -0.08 |  |  |  | | | |
| *eLAVL3* | -2.10 | 0.0418 | 1 | -0.06 |  |  |  | | | |
| *TBX10* | -2.10 | 0.0418 | 1 | -0.08 |  |  |  | | | |
| *RSAD2* | -2.10 | 0.0418 | 1 | -0.18 |  |  |  | | | |
| *LRRC16A* | -2.10 | 0.0419 | 1 | -0.15 |  |  |  | | | |
| *CLVS2* | -2.10 | 0.0419 | 1 | -0.06 |  |  |  | | | |
| *WDR62* | -2.10 | 0.0421 | 1 | -0.06 |  |  |  | | | |
| *C12orf53* | -2.10 | 0.0421 | 1 | -0.07 |  |  |  | | | |
| *TMeM74* | -2.10 | 0.0422 | 1 | -0.06 |  |  |  | | | |
| *F9* | -2.09 | 0.0426 | 1 | -0.04 |  |  |  | | | |
| *NR2F2* | -2.09 | 0.0428 | 1 | -0.06 |  |  |  | | | |
| *ASAP3* | -2.09 | 0.0429 | 1 | -0.05 |  |  |  | | | |
| *LRRC52* | -2.09 | 0.0429 | 1 | -0.05 |  |  |  | | | |
| *ReeP6* | -2.09 | 0.0430 | 1 | -0.06 |  |  |  | | | |
| *IRX3* | -2.09 | 0.0431 | 1 | -0.06 |  |  |  | | | |
| *C7orf27* | -2.08 | 0.0438 | 1 | -0.06 |  |  |  | | | |
| *PPIP5K1* | -2.08 | 0.0440 | 1 | -0.10 |  |  |  | | | |
| *FLJ34690* | -2.08 | 0.0440 | 1 | -0.05 |  |  |  | | | |
| *ATP6V0D2* | -2.08 | 0.0441 | 1 | -0.05 |  |  |  | | | |
| *LAMC3* | -2.08 | 0.0442 | 1 | -0.08 |  |  |  | | | |
| *SCTR* | -2.08 | 0.0443 | 1 | -0.06 |  |  |  | | | |
| *OR51B4* | -2.07 | 0.0444 | 1 | -0.08 |  |  |  | | | |
| *DNAJB13* | -2.07 | 0.0445 | 1 | -0.07 |  |  |  | | | |
| *C21orf67* | -2.07 | 0.0445 | 1 | -0.07 |  |  |  | | | |
| *NMUR2* | -2.07 | 0.0446 | 1 | -0.08 |  |  |  | | | |
| *SNTA1* | -2.07 | 0.0448 | 1 | -0.09 |  |  |  | | | |
| *CLDN1* | -2.07 | 0.0448 | 1 | -0.07 |  |  |  | | | |
| *GAP43* | -2.07 | 0.0451 | 1 | -0.08 |  |  |  | | | |
| *OR52B6* | -2.07 | 0.0451 | 1 | -0.06 |  |  |  | | | |
| *ZP2* | -2.07 | 0.0452 | 1 | -0.05 |  |  |  | | | |
| *SFTPD* | -2.06 | 0.0456 | 1 | -0.09 |  |  |  | | | |
| *OAS3* | -2.06 | 0.0459 | 1 | -0.21 |  |  |  | | | |
| *KCP* | -2.06 | 0.0460 | 1 | -0.06 |  |  |  | | | |
| *LOC100132354* | -2.06 | 0.0461 | 1 | -0.06 |  |  |  | | | |
| *OR8K5* | -2.06 | 0.0462 | 1 | -0.11 |  |  |  | | | |
| *ARID1A* | -2.06 | 0.0462 | 1 | -0.04 |  |  |  | | | |
| *DNeR* | -2.05 | 0.0463 | 1 | -0.06 |  |  |  | | | |
| *XKR3* | -2.05 | 0.0464 | 1 | -0.05 |  |  |  | | | |
| *TSNARe1* | -2.05 | 0.0468 | 1 | -0.07 |  |  |  | | | |
| *TDRG1* | -2.05 | 0.0469 | 1 | -0.07 |  |  |  | | | |
| *STGC3* | -2.05 | 0.0470 | 1 | -0.05 |  |  |  | | | |
| *SePN1* | -2.05 | 0.0470 | 1 | -0.08 |  |  |  | | | |
| *COL12A1* | -2.05 | 0.0470 | 1 | -0.05 |  |  |  | | | |
| *ADCY1* | -2.04 | 0.0475 | 1 | -0.06 |  |  |  | | | |
| *LGALS3BP* | -2.04 | 0.0475 | 1 | -0.13 |  |  |  | | | |
| *DLX5* | -2.04 | 0.0477 | 1 | -0.06 |  |  |  | | | |
| *SRRM1* | -2.04 | 0.0477 | 1 | -0.06 |  |  |  | | | |
| *CAPN5* | -2.04 | 0.0479 | 1 | -0.07 |  |  |  | | | |
| *OR7e19P* | -2.03 | 0.0484 | 1 | -0.11 |  |  |  | | | |
| *TAP2* | -2.03 | 0.0485 | 1 | -0.06 |  |  |  | | | |
| *SLC22A24* | -2.03 | 0.0485 | 1 | -0.05 |  |  |  | | | |
| *S100A16* | -2.03 | 0.0488 | 1 | -0.07 |  |  |  | | | |
| *BRD4* | -2.03 | 0.0489 | 1 | -0.06 |  |  |  | | | |
| *FAM159B* | -2.03 | 0.0490 | 1 | -0.05 |  |  |  | | | |
| *LOC728613* | -2.03 | 0.0490 | 1 | -0.07 |  |  |  | | | |
| *MT4* | -2.03 | 0.0491 | 1 | -0.06 |  |  |  | | | |
| *eLMOD1* | -2.03 | 0.0491 | 1 | -0.07 |  |  |  | | | |
| *RNU5F* | -2.03 | 0.0491 | 1 | -0.17 |  |  |  | | | |
| *TUBB8* | -2.03 | 0.0492 | 1 | -0.07 |  |  |  | | | |
| *ZNF556* | -2.03 | 0.0493 | 1 | -0.06 |  |  |  | | | |
| *TNFRSF14* | -2.03 | 0.0493 | 1 | -0.07 |  |  |  | | | |
| *ePHB2* | -2.03 | 0.0494 | 1 | -0.13 |  |  |  | | | |
| *LONRF2* | -2.02 | 0.0494 | 1 | -0.06 |  |  |  | | | |
| *OVCH1* | -2.02 | 0.0497 | 1 | -0.05 |  |  |  | | | |
| *PDZD7* | -2.02 | 0.0498 | 1 | -0.07 |  |  |  | | | |
