# Supplementary material for: Preliminary evidence for association of genetic variants in pri-miR-34b/c and abnormal miR-34c expression with attention deficit and hyperactivity disorder
Source: Transl Psychiatry. 2016 Aug 30;6(8):e879–. doi: 10.1038/tp.2016.151 (PMC5022091; doi:10.1038/tp.2016.151)
Supplement: Supplementary Table 6 [file tp2016151x7.doc]

**Supplementary Table 6** Genes that have previously been associated with ADHD and with other psychiatric disorders by either (**a**) candidate-gene association studies (CGAS) and/or (**b**) genome-wide association studies (GWAS) (coincidence with a GWAS top finding or close vicinity).

**a)**

| **Gene Information** | | | | **Psychiatric disorders Associated with Candidate Gene** | | | | |
| --- | --- | --- | --- | --- | --- | --- | --- | --- |
| **Gene** | **Variant/s (ADHD associated allele)** | **Relative**  **Position** | **ADHD** | **Schizophrenia** | **Bipolar Disorder** | **Autism Specttrum Disorders** | **Major Depression** | **Substance Use Disorders** |
| ***ADRA2A*** | c.-1291C>G (G) | 1.3kb 5’  Promoter | Comings et al., 2003^1^; Roman et al., 2006^2^; Park et al., 2005^3^; Schmitz et al., 2006^4^ | Lochman et al., 2013^5^ | - | - | Fukutake et al., 2008^6^ | Merenäkk et al., 2011^7^; Clarke et al., 2012^8^; Prestes et al., 2007^9^ |
| ***ADRA2C*** | CA(n) | 6kb 5’ | Comings et al., 1999^10^ | - | - | - | - | - |
| ***BAIAP2*** | rs8079781  rs4969385 | Intron | Ribasés et al., 2009^11^ | - | - | Toma et al., 2011^12^ | - | - |
| ***BDNF*** | p.Val66Met (Val) | Exon | Kent et al., 2005^13^ | Krebs et al., 2000^14^; Xu et al., 2008^15^ | Södersten et al., 2014^16^; Dell’Osso et al., 2014^17^ | Yoo et al., 2014^18^, Raznahan et al., 2009^19^; Hashimoto et al., 2006^20^ | Shimizu et al., 2003^21^; Molendijk et al., 2014^22^ | Corominas-Roso et al., 2013^23^; Kim et al., 2005^24^; Ghitza et al., 2010^25^ |
|  | c.720C>T (T) | Intron | Xu et al., 2007^26^ |  |  |  |  |  |
| ***CNTFR*** | rs7036351 | 3kb 5’ | Ribasés et al., 2008^27^ | - | - | - | - | - |
| ***COMT*** | p.Val108Met (Met) | Exon | Reuter et al., 2006^28^; Gothelf et al., 2007^29^ | Al-Asmary et al., 2014^30^; Glatt et al., 2003^31^ | Pandolfo et al., 2015^32^; Massat et al., 2011^33^ | Yoo et al., 2013^34^; Guo et al., 2013^35^ | Pandolfo et al., 2015^32^; Massat et al., 2011^33^ | Munafo et al., 2008^36^; Schellekens et al., 2012^37^ |
| ***DBH*** | Taq I (A1) | Intron | Smith et al., 2003^37^ | Cubells et al., 2011^38^ | Ates et al., 2013^39^ | - | Preuss et al., 2013^40^; Zhou et al., 2015^41^ | Preuss et al., 2013^40^; Freire et al., 2006^42^ |
|  | Taq I (A2) | Intron | Daly et al., 1999^43^; Roman et al., 2002^44^ |  |  |  |  |  |
| ***DDC*** | rs6592961 | Intron | Ribasés et al., 2009^45^ | Børglum et al., 2001^46^ | Børglum et al., 1999^47^; Børglum et al., 2003^48^ | Toma et al., 2013^49^ | - | Ma et al., 2005^50^ |
| ***DRD2*** | Taq I (A1) | 9.5kb 3’  (Exon 8 ANKK1) | Comings et al., 1991^51^ and 1996^52^; Sery et al., 2006^53^ | Liu et al., 2014^54, 55^; Kaalund et al., 2014 | Zou et al., 2010^56^; Massat et al., 2002^57^; Li et al., 1999^58^ | Hettinger et al., 2012^59^; Salem et al., 2013^60^ | Kaalund et al., 2014^55^ | Schellekens et al., 2012^37^; Freire et al., 2006^42^ |
| ***DRD4*** | VNTR-48bp (7R) | Exon | Arcos-Burgos et al., 2004^61^; Faraone et al., 2005^62^; Li et al., 2006^63^; Gizer et al., 2009^64^ | Lung et al., 2009^65^; Lai et al., 2010^66^; Kordi-Tamandani et al., 2013^67^ | Gonçalves et al., 2012^68^ | Reiersen et al., 2011^69^ | López et al., 2005^70^; Lai et al., 2010^66^ | Lai et al., 2010^66^; Chen et al., 2011^71^; van der Zwaluw et al., 2012^72^ |
|  | Dup-120bp (240bp) | Promoter | McCracken et al., 2000^73^; Kustanovich et al., 2004^74^ |  |  |  |  |  |
|  | c.521C>T(A) | Promoter | Bellgrove et al., 2005^75^ |  |  |  |  |  |
|  | c.616C>G (C) | Promoter | Lowe et al., 2004^76^ |  |  |  |  |  |
| ***DRD5*** | Microsatellite (148bp) | 18.5kb 5’ | Tahir et al., 2000^77^; Li et al., 2006^63^; Hawi et al., 2003^78^; Lowe et al., 2004^79^; Gizer et al., 2009^64^ | Zhao et al., 2014^80^; Muir et al., 2001^81^ | - | Taurines et al., 2011^82^ | - | - |
| ***GRIN2A*** | p.Leu325Leu | Exon | Turic et al., 2004^83^ | Tang et al., 2006^84^; Itokawa et al., 2003^85^ | Itokawa et al., 2003^86^ | Barnby et al., 2005^87^; Yoo et al., 2012^88^ | Kaut et al., 2015^89^ | Zhao et al., 2013^90^; Domart et al., 2012^91^ |
| ***HTR1A*** | c.1438A>G (G) | Promoter | Li et al., 2006^63^ | Zhou et al., 2013^92^; Tang et al., 2014^93^ | Kishi et al., 2011^94^; Kishi et al., 2012^95^ | Sener et al., 2016^96^ | Kishi et al., 2012^95^ | Zuo et al., 2013^97^ |
| ***HTR2A*** | rs7984966 | Intron | Ribasés et al., 2009^45^ | Ghadirivasfi et al., 2011^98^; Sujitha et al., 2014^99^; Tan et al., 2014^100^ | Ghadirivasfi et al., 2011^98^; Tan et al., 2014^100^; McAuley et al., 2009^101^; Xiang et al., 2014^102^ | Smith et al., 2014^103^; Nyffeler et al., 2014^104^ | Tan et al., 2014^100^;  Du et al., 2000^105^ | Cao et al., 2014^106^; Wrzosek et al., 2012^107^ |
|  | c.102C>T (T) | Exon | Levitan et al., 2002^108^ |  |  |  |  |  |
|  | p.His452Tyr | Exon | Guimaraes et al., 2007^109^ |  |  |  |  |  |
| ***HTR1B*** | c.861G>C (C) | Exon | Hawi et al., 2002^110^; Quist et al., 2003^111^; Smoller et al., 2006^112^; Gizer et al., 2009^64^ | - | - | - | Huang et al., 2003^113^ | Huang et al., 2003^113^; Rocha et al., 1998^114^ |
| ***HTR2C*** | c.697G>C (G)  c.759C>T (C) | Promoter | Li et a., 2006^115^; Xu et al., 2009^116^; | - | - | - | Kõks et al., 2006^117^ | Yasseen et al., 2010^118^; Anastasio et al., 2014^119^ |
| ***LPHN3*** | rs6551665  rs1947274  rs2345039 | Intron | Arcos-Burgos et al., 2010^120^ | - | - | - | - | - |
|  | rs2122643 | Intron | Ribasés et al., 2010^121^ |  |  |  |  |  |
| ***MAO-A*** | VNTR-30bp (4R-5R) | Promoter | Manor et al., 2002^122^ | Qiu et al., 2009^123^; Jönsson et al., 2003^124^ | Preisig et al., 2000^125^; Eslami et al., 2015^126^ | Cohen et al., 2003^127^; Yoo et al., 2009^128^; Bortolato et al., 2012 ^129^ | Dannlowski et al., 2009^130^; Lung et al., 2011^131^ | Philibert et al., 2008^132^; Nilsson et al., 2011^133^ |
|  | Microsatellite CA(n) (6R) | Intron | Jiang et al., 2001^134^ |  |  |  |  |  |
|  | c.941G>T (G) | Exon | Domschke et al., 2005^135^; Xu et al., 2007^136^; Brookes et al., 2006^137^ |  |  |  |  |  |
| ***MAO-B*** | rs3027415 | 8.3kb 3’ | Ribasés et al., 2009^45^ | Carrera et al., 2009^138^; Sun et al., 2012^139^; | Lin et al., 2000^140^ | Bortolato et al., 2012^129^ | - | Launay et al., 2009^141^; Ho et al., 2009^142^ |
| ***NTF3*** | rs6332 | Exon | Ribasés et al., 2008^27^ | Durany et al., 2001^143^; Virgos et al., 2001^144^ | Fernandes et al., 2010^145^; Loch et al., 2015^146^ | Sajdel-Sulkowska et al., 2009^147^; Tostes et al., 2012^148^ | Hock et al., 2000^149^; Otsuki et al., 2008^150^ | Pierce et al., 1999^151^; Akbarian et al., 2001^152^ |
| ***NTRK2*** | rs1387926 | Intron | Ribasés et al., 2008^27^ | Hashimoto et al., 2005^153^; Ray et al., 2014^154^ | Soontornniyomkij et al., 2011^155^; Ray et al., 2014^154^ | Correia et al., 2010^156^; Chandley et al., 2015^157^ | Murphy et al., 2012^158^; Ray et al., 2014^154^ | Xu et al., 2007^159^; Beuten et al., 2007^160^ |
| ***SLC6A2 (NET)*** | rs3785157 (G/T) | Intron | Xu et al., 2005^161^; Bobb et al., 2005^162^ | Siuta et al., 2010^163^; | - | - | Haenisch et al., 2009^164^; Kim et al., 2014^165^ | Mash et al., 2005^166^; Clarke et al., 2012^8^ |
|  | rs998424 (A) | Intron | Bobb et al., 2005^162^ |  |  |  |  |  |
|  | rs3785143  rs11568324 | Intron | Brookes et al., 2006^137^ |  |  |  |  |  |
|  | c.-3081A>T (T) | 3.1kb 5’ | Kim et al., 2006^167^ |  |  |  |  |  |
| ***SLC6A3 (DAT)*** | VNTR-480bp (10R) | 3’UTR | Cook et al., 1995^168^; Faraone et al., 2005^62^; Purper-Ouakil D et al., 2005^169^; Mill et al., 2005^170^; Yang et al., 2007^171^; Gizer et al., 2009^64^ | Rao et al., 2012^172^; Liu et al., 2013^173^; Markota et al., 2014^174^ | Rao et al., 2012^172^; Huang et al., 2015^175^ | Nakamura et al., 2010^176^; Hamilton et al., 2013^177^ | Haeffel et al., 2008^178^; Amsterdam et al., 2012^179^ | Stolf et al., 2014^180^; Vasconcelos et al., 2015^181^ |
| ***SLC6A4 (HTT)*** | 5’HTTLPR (L) | 5’ UTR | Curran et al., 2005^182^; Manor et al., 2001^183^ | Fan et al., 2005^184^; Li et al., 2013^185^ | Jiang et al., 2013^186^; Benedetti et al., 2015^187^ | Wassink et al., 2007^188^; Wiggins et al., 2012^189^ | Haenisch et al., 2013^190^; Little et al., 2014^191^ | Konishi et al., 2004^192^; Cao et al., 2013^193^ |
|  | 5’HTTLPR (S) | 5’ UTR | Li et al., 2007^194^ |  |  |  |  |  |
|  | VNTR-17bp (12R) | Intron | Banerjee et al., 2006^195^ |  |  |  |  |  |
| ***SLC9A9*** | inv(3)(p14:q21) | - | Da Silva et al., 2003^196^ | - | - | Kondapalli et al., 2013^197^; Schwede et al., 2014^198^ | - | - |
| ***SNAP25*** | c.1069T>C  c.1065T>G | 3’ UTR | Barr et al., 2000^199^; Brophy et al., 2002^200^; Kustanovich et al., 2003^201^ | Fatemi et al., 2001^202^; Lochman et al., 2013^5^; Wang et al., 2015^203^ | Fatemi et al., 2001^202^; Etain et al., 2010^204^ | Braida et al., 2015^205^ | Wang et al., 2015^203^ | Lull et al., 2009^206^ |
|  | rs362549 (A)  rs362987 (A)  rs362998 (C) | Intron  Exon | Feng et al., 2005^207^ |  |  |  |  |  |
|  | rs3746544 | 3’UTR | Choi et al., 2007^208^; Brophy et al., 2002^200^; Gizer et al., 2009^64^ |  |  |  |  |  |
| ***TPH2*** | rs1843809 (T)  rs1386497 (A) | Intron | Sheehan et al., 2005^209^ | De Luca et al., 2005^210^; Xu et al., 2014^211^ | De Luca et al., 2005^210^; Roche et al., 2009^212^; Xiang et al., 2014^102^ | Coon et al., 2005^213^; Yang et al., 2012^214^ | Van Den Bogaert et al., 2006^215^; Gao et al., 2012^216^ | Reuter et al., 2007^217^; Nielsen et al., 2008^218^ |
| ***VAMP2*** | 26 bp Ins/Del (Ins) | - | Kenar et al., 2014^219^; Gao et al., 2015^220^ | - | - | - | Yamada et al., 2002^221^; Malki et al., 2014^222^ | Varodayan et al., 2013^223^ |

b)

| **Gene Information** | | | | **Psychiatric disorders Associated with Candidate Gene/Variant** | | | | |
| --- | --- | --- | --- | --- | --- | --- | --- | --- |
| **Gene** | **Variant/s** | **Relative**  **Position** | **ADHD GWAS** | **Schizophrenia** | **Bipolar Disorder** | **Autism Specttrum Disorders** | **Major Depression** | **Substance Use Disorders** |
| ***AK094352*** | rs7175404 | Intron | Lesch et al., 2008^224^ | - | - | - | Holmans et al., 2007^225^ | - |
| ***AK128216*** | rs12679254 | 10kb | Lasky-Su et al., 2008^226^ | - | - | - | - | - |
| ***ATP2C2*** | rs10514604 | Intron | Lesch et al., 2008^224^; Zhou et al., 2008^227^ | - | - | Eicher et al., 2015^228^ | - | - |
| ***ASTN2*** | rs10983238 | Intron | Lesch et al., 2008^224^ | Vrijenhoek et al., 2008^229^; Sullivan et al., 2008^230^; Wang et al., 2010^231^ | Wang et al., 2010^231^ | Marshall et al., 2008^232^; Glessner et al., 2009^233^; Lionel et al., 2014^234^ | - | Uhl et al., 2008^235^ |
| ***ATXN2*** | rs616668 | Intron | Stergiakouli et al., 2012^236^ | Zhang et al., 2014^237^ | - | - | - | - |
| ***AURKC1*** | rs2014572 | 15kb 3' | Lasky-Su et al., 2008^226^ | - | - | - | - | - |
| ***BAALCOS*** | rs6983777 |  | Ebejer et al., 2013^238^ | - | - | - | - | - |
| ***BC032407*** | rs1918172 | Intron | Lasky-Su et al., 2008^226^ | - | - | - | - | - |
| ***BCL11A*** | rs2556378 | Intron | Hinney et al., 2011^239^ | - | - | - | Alisch et al., 2014^240^ | - |
| ***C9orf98*** | rs11243897 | Intron | Lesch et al., 2008^224^ | - | - | - | - | - |
| ***C10orf79*** | rs515910 | Intron | Lesch et al., 2008^224^ | - | - | - | - | - |
| ***CDH13*** | rs7187223 | 203kb 5' | Neale et al., 2008^241^; Lasky-Su et al., 2008^226^; Lesch et al., 2008^224^; Zhou et al., 2008^227^ | Sullivan et al., 2008^230^ | Xu et al., 2014^242^ | Christian et al., 2008^243^ | Edwards et al., 2012^244^ | Uhl et al., 2008^235^; Edwards et al., 2012^244^; Yang et al., 2015^245^ |
|  | rs6565113 | Intron | Neale et al., 2008^241^; Lasky-Su et al., 2008^226^; Lesch et al., 2008^224^; Zhou et al., 2008^227^ |  |  |  |  |  |
|  | rs11646411 | Intron | Lesch et al., 2008^224^; Zhou et al., 2008^227^; Lasky-Su et al., 2008^226^ |  |  |  |  |  |
| ***CDH23*** | rs11594082 | Intron | Lesch et al., 2008^224^; | Sullivan et al., 2008^230^ | - | - | - | - |
| ***CHMP7*** | rs7463256 | Intron | Neale et al., 2010^246^ | - | - | - | - | - |
| ***CLYBL*** | rs7992643 | 15kb 3' | Lasky-Su et al., 2008^226^ | Blouin et al., 1998^247^ | Detera-Wadkeigh et al., 1999^248^ | - | - | - |
| ***CNR1*** | rs964647 | 80kb 3' | Neale et al., 2008^241^; Lasky-Su et al., 2008^226^; Zhou et al., 2008^227^ | Chavarría-Siles et al., 2008^249^; Ho et al., 2011^250^ | - | - | Monteleone et al., 2010^251^; Mitjans et al., 2013^252^ | Ponce et al., 2003^253^; Zuo et al., 2007^254^; Rotter et al., 2013 ^255^ |
| ***CNTNAP4*** | rs13330107 | 1.7kb 5’ | Lasky-Su et al., 2008^226^ | - | - | - | - | - |
| ***CNTNAP5*** | rs17367118 | 1,1mb 5’ | Lasky-Su et al., 2008^226^ |  | Djurovic et al., 2010^256^ | Pagnamenta et al., 2010^257^ |  |  |
| ***CPLX2*** | rs7448069 | Intron | Ebejer et al., 2013^238^ | Ramos-Miguel et al., 2014^258^; Zakharyan et al., 2014^259^ | - | - | - | - |
| ***CTNNA2*** | rs13395022 | Intron | Lesch et al., 2008^224^; Neale et al., 2008^241^ | Lewis et al., 2003^260^; Sullivan et al., 2008^230^ | Wellcome Trust Case Control Consortium 2007^261^ | - | - | - |
| ***CREB5*** | rs2237349 | Intron | Lesch et al., 2008^224^ | Sullivan et al., 2008^230^ | Wellcome Trust Case Control Consortium 2007^261^ | - | - | - |
| ***CRYGC*** | rs2242073 | Intron | Lesch et al., 2008^224^ | - | - | - | - |  |
| ***CSMD2*** | rs2281597 | Intron | Lesch et al., 2008^224^ | Håvik et al., 2011^262^ | - | - | Edwards et al., 2012^244^ | Uhl et al., 2008^235^; Johnson et al., 2006^263^ |
| ***DCLK1/DCAMKL1*** | rs1539549 | Intron | Neale et al., 2008^241^ | Håvik et al., 2012^264^ | Håvik et al., 2012^264^ | - | - | - |
| ***DMRT2*** | rs17641078 | Exon | Lasky-Su et al., 2008^226^ | - | - | Allen-Brady et al., 2009^265^; Szatmari et al., 2007^266^ | - | Li et al., 2008^267^ |
| ***DNM1*** | rs2502731 | Intron | Lesch et al., 2008^224^ | - | - | Szatmari et al., 2007^266^ | - | Yang et al., 2015^245^ |
| ***DNMT3B*** | rs6057648 | Intron | Ebejer et al., 2013^238^ | Zhang et al., 2009^268^; Saradalekshmi et al., 2014^269^ | - | - | Higuchi et al., 2011^270^ | - |
| ***DPP6*** | rs2110267 | 48kb 5’ | Ebejer et al., 2013^238^ | - | - | Marshall et al., 2008^232^; Egger et al., 2014^271^ | - | - |
| ***DPP10*** | rs272000 | 50kb 3' | Lasky-Su et al., 2008^226^ | Lewis et al., 2003^260^; Sullivan et al., 2008^230^ | Wellcome Trust Case Control Consortium 2007^261^; Djurovic et al., 2010^256^ | Marshall et al., 2008^232^; Girirajan et al., 2013^272^ | - | - |
| ***EREG*** | rs1350666 | 15kb 3' | Lasky-Su et al., 2008^226^ | - | - | - | - | - |
| ***FAM19A3*** | rs11590090 | 60kb 3' | Lasky-Su et al., 2008^226^ | Arinami et al., 2005^273^; Lewis et al., 2003^260^ | - | - | - | - |
| ***FAM189A1*** | rs1471225 | 25kb 5’ | Lasky-Su et al., 2008^226^ | - | - | - | - | - |
| ***FHIT*** | rs6791644 | Intron | Lasky-Su et al., 2008^226^ | Sullivan et al., 2008^230^ | - | Sebat et al., 2007; Marshall et al., 2008^232^; Girirajan et al., 2013^272^ | - | - |
| ***FOXP1*** | rs17651978 | Intron/5'UTR | Lasky-Su et al., 2008^226^ | Sullivan et al., 2008^230^ | Sklar et al., 2008^274^; Nurnberger et al., 2014^275^ | Chien et al., 2013^276^ | - | - |
| ***FRMD1*** | rs9364220 | 10kb 5’ | Ebejer et al., 2013^238^ | - | - | - | - | - |
| ***GFOD1*** | rs552655 | Intron | Lasky-Su et al., 2008^226^ | Lewis et al., 2003^260^ | - | - | - | - |
| ***GPC6*** | rs7995215 | Intron | Lesch et al., 2008^224^ | Sullivan et al., 2008^230^; Wang et al., 2012^277^ | Sklar et al., 2008^274^ | Marshall et al., 2008^232^ | - | - |
| ***GPR139*** | rs12919130 | 41kb 5’ | Ebejer et al., 2013^238^ | Castellani et al., 2014^278^ | - | - | - | - |
| ***GRIK1*** | rs363512 | Intron | Lasky-Su et al., 2008^226^ | Hirata et al., 2012^279^ | Wellcome Trust Case Control Consortium 2007^261^ | - | - | Kranzler et al., 2009^280^ |
| ***GRIK4*** | rs4245040 | Intron | Yang et al., 2013^281^ | Pickard et al., 2006^282^ | Pickard et al., 2008^283^ | Griswold et al., 2012^284^ | - | - |
| ***GRM5*** | rs5016282 | Intron | Hinney et al., 2011^239^ | Ohnuma et al., 1998^285^; Devon et al., 2001^286^ | - | Zantomio et al., 2015^287^ | Deschwanden et al., 2011^288^; Chandley et al., 2014^289^ | Hulka et al., 2014^290^; Milella et al., 2014^291^ |
| ***GUCY1A2*** | rs10895959 | 214kb 3’ | Lasky-Su et al., 2008^226^ | - | - | - | - | - |
| ***HAS3/TMCO7*** | rs8047014 | 5kb 5'/20kb 3' | Lasky-Su et al., 2008^226^; Zhou et al., 2008^227^; | - | - | - | - | - |
| ***HOXB1*** | rs11079828 | 0.8kb 5’ | Stergiakouli et al., 2012^236^ | - | - | - | - | - |
| ***IL16*** | rs7172689 | Intron | Lasky-Su et al., 2008^226^; Neale et al., 2008^241^ | - | - | - | - | - |
| ***IL20RA*** | rs1744062 | Intron | Stergiakouli et al., 2012^236^ | - | - | - | - | - |
| ***ITGA11*** | rs7164335 | Intron | Lesch et al., 2008^224^ | - | - | Szatmari et al., 2007^266^ | - | - |
| ***ITGAE*** | rs220470 | Intron | Lesch et al., 2008^224^; Zhou et al., 2008^227^ | - | - | - | - | - |
| ***ITPR2*** | rs3782309 | Intron | Neale et al., 2008^241^; Lasky-Su et al., 2008^226^ | - | Nurnberger et al., 2014^275^ | - | - | - |
| ***KANSL1*** | rs2532274 | Intron | Hinney et al., 2011^239^ | - | - | - | - | - |
| ***KCNC1*** | rs3893215 | Intron | Lesch et al., 2008^224^ | Yanagi et al., 2014^292^ | - | Trikalinos et al., 2006^293^; Duvall et al., 2007^294^; Szatmari et al., 2007^266^ | - | Gelernter et al., 2014^295^ |
| ***KIAA0574*** | rs1471225 | 30kb 3' | Lasky-Su et al., 2008^226^; Zhou et al., 2008^227^ | - | - | Marshall et al., 2008^232^; Sebat et al., 2007^296^; Christian et al., 2008^243^ | - | - |
| ***KCNIP1*** | rs1541665 | Intron | Neale et al., 2008^241^; Lasky-Su et al., 2008^226^ | Sullivan et al., 2008^230^; Lewis et al., 2003^260^ | - | - | - | - |
| ***KCNIP4*** | rs876477 | Intron | Neale et al., 2008^241^; Lasky-Su et al., 2008^226^ | Sullivan et al., 2008^230^ | Sklar et al., 2008^274^ | - | - | - |
| ***LARGE*** | rs130575 | 543kb 5’ | Lasky-Su et al., 2008^226^ | - | - | - | - | - |
| ***LINC00494*** | rs910191 | 89kb 3’ | Ebejer et al., 2013^238^ | - | - | - | - | - |
| ***LINC01183*** | rs1515641 | Intron | Ebejer et al., 2013^238^ | - | - | - | - | - |
| ***LPL*** | rs7816032 | 15kb 5' | Lasky-Su et al., 2008^226^ | Le-Niculescu et al., 2007^297^; Xie et al., 2011^298^ | - | - | - | - |
| ***LRRC7*** | rs4650135 | 8.7kb 3’ | Lasky-Su et al., 2008^226^ | - | - | - | - | - |
| ***MAGI2*** | rs3779312 | Intron | Stergiakouli et al., 2012^236^ | Koide et al., 2012^299^; Karlsson et al., 2012^299^ | - | - | - | - |
| ***MAN2A2*** | rs2677744 | Intron | Lesch et al., 2008^224^ | - | - | Szatmari et al., 2007^266^ | Holmans et al., 2007^225^ | - |
| ***MAP1B*** | rs2199161 | Intron | Lesch et al., 2008^224^; Zhou et al., 2008^227^ | - | Bouras et al., 2011^300^ | - | - | - |
| ***MAPRE1*** | rs6057651 | Intron | Ebejer et al., 2013^238^ | - | - | - | - | - |
| ***MBOAT1*** | rs1202199 | Intron | Lasky-Su et al., 2008^226^ | Lewis et al., 2003^260^ | - | - | - | - |
| ***MEIS2*** | rs8041675 | Intron | Lasky-Su et al., 2008^226^; Bakker et al., 2003; Zhou et al., 2008^227^ | - | - | - | - | - |
| ***MYTIL*** | rs2241685 | Intron | Lesch et al., 2008^224^ | - | - | - | - | - |
| ***MGC48628*** | rs12505502 | Intron | Neale et al., 2008^241^; Lasky-Su et al., 2008^226^ | - | Wellcome Trust Case Control Consortium 2007^261^ | - | - | - |
| ***MMP24*** | rs1555322 | Intron | Lesch et al., 2008^224^ | - | - | Allen-Brady et al., 2008^265^ | - | - |
| ***MOBP*** | rs864643 | Intron/3' UTR | Lesch et al., 2008^224^ | Lewis et al., 2003^260^; Mitkus et al., 2008^301^ | - | - | Aston et al., 2005^302^ | Albertson et al., 2004^303^; Mitkus et al., 2008^301^ |
| ***MTA3*** | rs930421 | Exon | Lasky-Su et al., 2008^226^ | - | - | - | - | - |
| ***MYT1L*** | rs6719977 | 2kb 3' | Lasky-Su et al., 2008^226^ | - | - | - | - | - |
|  | rs2241685 | Intron | Lesch et al., 2008^224^ | Vrijenhoek et al., 2008^229^; Lee et al., 2012^304^ | - | Meyer et al., 2012^305^ | Wang et al., 2010^306^ | - |
| ***NAP5*** | rs7577925 | Intron | Lasky-Su et al., 2008^226^ | Lewis et al., 2003^260^; Wang et al., 2010^306^ | Smith et al., 2009^307^; Wang et al., 2010^306^ | - | - | - |
| ***NAPRT1*** | rs2290416 | Exon | Lasky-Su et al., 2008^226^ | - | - | - | - | - |
| ***NCKAP5*** | rs7577925 | Intron | Lesch et al., 2008^224^ | - | - | - | Luciano et al., 2012^308^ | - |
| ***NDN*** | rs7164923 | 130kb 5’ | Ebejer et al., 2013^238^ | - | - | - | - | - |
| ***NR4A2*** | rs1918172 | Intron | Lasky-Su et al., 2008^226^ | Xing et al., 2006^309^;  Guillozet-Bongaarts et al., 2014^310^ | Xing et al., 2006^309^ | Chuang et al., 2015^311^ | Xing et al., 2006^309^; Kerman et al., 2012^312^ | Bannon et al., 2002^313^; Wei et al., 2012^314^ |
| ***NT5DC3*** | rs4964805 | Intron | Lesch et al., 2008^224^; Rommelse, 2008 | - | - | - | - | - |
| ***NOS1*** | rs478597 | Intron | Lasky-Su et al., 2008^226^ | Reif et al., 2006^315^; Candemir et al., 2016^316^ | Reif et al., 2006^315^; Kittel-Schneider et al., 2014^317^ | Kim et al., 2009^318^ | Abkevich et al., 2003^319^; Galecki et al., 2011^320^ | - |
| ***NUCB1*** | rs9676447 | Intron | Neale et al., 2008^241^ | - | - | - | - | - |
| ***OTOL1*** | rs6808138 | 170kb 3’ | Lasky-Su et al., 2008^226^ | - | - | - | - | - |
| ***PARD3B*** | rs11681930 | Intron | Ebejer et al., 2013^238^ | - | - | - | - | - |
| ***PDCP1=MGC33657*** | rs2587695 | Intron | Lesch et al., 2008^224^ | Lewis et al., 2003^260^ | - | - | - | - |
| ***PEX7/MAP3K5*** | rs6919857 | 5kb 5'/27kb 3' | Neale et al., 2008^241^; Lasky-Su et al., 2008^226^ | - | - | Ro et al., 2012^321^; Yu et al., 2013^322^ | - | - |
| ***PPM1F*** | rs412050 | 300bp 3' | Lesch et al., 2008^224^ | Lewis et al., 2003^260^ | - | - | - | - |
| ***PSMC3*** | rs7105122 | 1.5kb 5’ | Hinney et al., 2011^239^ | - | - | - | - | - |
| ***PTPN14*** | rs6657749 | Intron | Neale et al., 2008^241^ | - | Sklar et al., 2008^274^ | - | - | Kane et al., 2004^323^ |
| ***PTPRN2*** | rs10227331 | 40kb | Lasky-Su et al., 2008^226^ | Sullivan et al., 2008^230^ | Sklar et al., 2008^274^ | Trikalinos et al., 2006^293^ | Yang et al., 2011^324^ | Yang et al., 2011^324^ |
| ***PTHLH*** | rs522958 | 103kb 5’ | Lasky-Su et al., 2008^226^ | - | - | - | - | - |
| ***RAB27B*** | rs2311120 | 20kb 5' | Neale et al., 2008^241^ | Maziade et al., 2005^325^ | - | - | - | - |
| ***RDH10*** | rs12679254 | Intron | Lasky-Su et al., 2008^226^ | - | - | - | - | - |
| ***REEP5*** | rs469727 | Intron | Lesch et al., 2008^224^ | - | - | - | Yang et al., 2012^326^ | - |
| ***RHOC*** | rs11590090 | Intron | Lasky-Su et al., 2008^226^ | - | - | - | - | - |
| ***RNF144B*** | rs41441749 | 332kb 5’ | Lasky-Su et al., 2008^226^ | - | - | - | - | - |
| ***RORA*** | rs922781 | Intron | Neale et al., 2008^241^ | - | Etain et al., 2014^327^; Lai et al., 2015^328^ | Hu et al., 2009^329^; Nguyen et al., 2010^330^ | Terracciano et al., 2010^331^; Ming et al., 2015^332^ | - |
| ***SDK2*** | rs12453316 | Intron | Lesch et al., 2008^224^ | - | - | - | - | - |
| ***SHFM1*** | rs1464807 |  | Neale et al., 2010^246^ | - | - | - | - | - |
| ***SLCO3A1*** | rs7495052 | Intron | Lasky-Su et al., 2008^226^ | - | - | Szatmari et al., 2007^266^ | Holmans et al., 2007^225^; Verma et al., 2008^333^ | Wang et al., 2012^334^ |
| ***SPATA13*** | rs17079773 | Intron | Lasky-Su et al., 2008^226^ | - | - | - | - | - |
| ***SPOCK3*** | rs7657608 | Intron | Neale et al., 2008^241^ | - | - | - | - | - |
| ***SUPT3H*** | rs3799977 | Intron | Lesch et al., 2008^224^; Zhou et al., 2008^227^ | Lewis et al., 2003^260^ | - | - | - | - |
| ***SYT16*** | rs1514928 | 110kb 3' | Lasky-Su et al., 2008^226^ | - | - | - | - | - |
| ***TEX41*** | rs1822881 |  | Ebejer et al., 2013^238^ | - | - | - | - | - |
| ***TFEB*** | rs2842643 | 1kb 3' | Lesch et al., 2008^224^; Zhou et al., 2008^227^ | Lewis et al., 2003^260^ | - | - | - | - |
| ***TGFB2*** | rs1018040 | 87kb 3’ | Lasky-Su et al., 2008^226^ | - | - | El-Ansary et al., 2012^335^ | - | - |
| ***TLE4*** | rs2769967 | 297kb 5’ | Lasky-Su et al., 2008^226^ | - | - | - | - | - |
| ***TLL2*** | rs10786284 | Intron | Lesch et al., 2008^224^ | - | de Mooij-van Malsen et al., 2013^336^ | - | - | - |
| ***TMX3*** | rs17232800 | 48kb 3' | Yang et al., 2013^281^ | - | - | - | - | - |
| ***TRIO*** | rs42259 | Intron | Stergiakouli et al., 2012^236^ | - | - | - | - | - |
| ***TRIQK*** | rs1027730 | 107kb 5’ | Neale et al., 2010^246^ | - | - | - | - | - |
| ***TRUB1*** | rs12772737 | 6kb 3' | Neale et al., 2008^241^ | - | - | - | - | - |
| ***TSHZ2*** | rs10485813 | 5kb 5’ | Neale et al., 2010^246^ | - | - | - | - | - |
| ***UNC5B*** | rs16928529 | Intron | Lesch et al., 2008^224^ | - | - | - | - | - |
| ***ZMAT4*** | rs11786458 | 135kb 3’ | Lasky-Su et al., 2008^226^ | - | - | - | - | - |
| ***ZNF385D*** | rs11719664 | Intron | Lasky-Su et al., 2008^226^ | Lewis et al., 2003^260^; Schwab et al., 2008^337^; Xu et al., 2013^338^ | Wellcome Trust Case Control Consortium 2007^261^ | - | - | - |
| ***ZNF423*** | rs17281813 | Intron | Lasky-Su et al., 2008^226^; Zhou et al., 2008^227^ | - | - | - | - | - |
| ***ZNF544*** | rs260461 | Intron/3'UTR | Lasky-Su et al., 2008^226^ | - | - | - | - | - |
| ***ZNF805*** | rs2014572 | Exon | Lasky-Su et al., 2008^226^ | - | - | - | - | - |

**Supplementary Bibliography**

1. Comings DE, Gonzalez NS, Cheng Li SC, MacMurray J. A "line item" approach to the identification of genes involved in polygenic behavioral disorders: the adrenergic alpha2A (ADRA2A) gene. *Am J Med Genet B Neuropsychiatr Genet* 2003; **118B**(1)**:** 110-114.

2. Roman T, Polanczyk GV, Zeni C, Genro JP, Rohde LA, Hutz MH. Further evidence of the involvement of alpha-2A-adrenergic receptor gene (ADRA2A) in inattentive dimensional scores of attention-deficit/hyperactivity disorder. *Mol Psychiatry* 2006; **11**(1)**:** 8-10.

3. Park L, Nigg JT, Waldman ID, Nummy KA, Huang-Pollock C, Rappley M*, et al*. Association and linkage of alpha-2A adrenergic receptor gene polymorphisms with childhood ADHD. *Mol Psychiatry* 2005; **10**(6)**:** 572-580.

4. Schmitz M, Denardin D, Silva TL, Pianca T, Roman T, Hutz MH*, et al*. Association between alpha-2a-adrenergic receptor gene and ADHD inattentive type. *Biol Psychiatry* 2006; **60**(10)**:** 1028-1033.

5. Lochman J, Balcar VJ, Stastny F, Sery O. Preliminary evidence for association between schizophrenia and polymorphisms in the regulatory Regions of the ADRA2A, DRD3 and SNAP-25 Genes. *Psychiatry Res* 2013; **205**(1-2)**:** 7-12.

6. Fukutake M, Hishimoto A, Nishiguchi N, Nushida H, Ueno Y, Shirakawa O*, et al*. Association of alpha2A-adrenergic receptor gene polymorphism with susceptibility to suicide in Japanese females. *Prog Neuropsychopharmacol Biol Psychiatry* 2008; **32**(6)**:** 1428-1433.

7. Merenakk L, Maestu J, Nordquist N, Parik J, Oreland L, Loit HM*, et al*. Effects of the serotonin transporter (5-HTTLPR) and alpha2A-adrenoceptor (C-1291G) genotypes on substance use in children and adolescents: a longitudinal study. *Psychopharmacology (Berl)* 2011; **215**(1)**:** 13-22.

8. Clarke TK, Dempster E, Docherty SJ, Desrivieres S, Lourdsamy A, Wodarz N*, et al*. Multiple polymorphisms in genes of the adrenergic stress system confer vulnerability to alcohol abuse. *Addict Biol* 2012; **17**(1)**:** 202-208.

9. Prestes AP, Marques FZ, Hutz MH, Roman T, Bau CH. Tobacco smoking and the ADRA2A C-1291G polymorphism. *J Neural Transm (Vienna)* 2007; **114**(11)**:** 1503-1506.

10. Comings DE, Gade-Andavolu R, Gonzalez N, Blake H, Wu S, MacMurray JP. Additive effect of three noradrenergic genes (ADRA2a, ADRA2C, DBH) on attention-deficit hyperactivity disorder and learning disabilities in Tourette syndrome subjects. *Clin Genet* 1999; **55**(3)**:** 160-172.

11. Ribases M, Bosch R, Hervas A, Ramos-Quiroga JA, Sanchez-Mora C, Bielsa A*, et al*. Case-control study of six genes asymmetrically expressed in the two cerebral hemispheres: association of BAIAP2 with attention-deficit/hyperactivity disorder. *Biol Psychiatry* 2009; **66**(10)**:** 926-934.

12. Toma C, Hervas A, Balmana N, Vilella E, Aguilera F, Cusco I*, et al*. Association study of six candidate genes asymmetrically expressed in the two cerebral hemispheres suggests the involvement of BAIAP2 in autism. *J Psychiatr Res* 2011; **45**(2)**:** 280-282.

13. Kent L, Green E, Hawi Z, Kirley A, Dudbridge F, Lowe N*, et al*. Association of the paternally transmitted copy of common Valine allele of the Val66Met polymorphism of the brain-derived neurotrophic factor (BDNF) gene with susceptibility to ADHD. *Mol Psychiatry* 2005; **10**(10)**:** 939-943.

14. Krebs MO, Guillin O, Bourdell MC, Schwartz JC, Olie JP, Poirier MF*, et al*. Brain derived neurotrophic factor (BDNF) gene variants association with age at onset and therapeutic response in schizophrenia. *Mol Psychiatry* 2000; **5**(5)**:** 558-562.

15. Xu MQ, St Clair D, Feng GY, Lin ZG, He G, Li X*, et al*. BDNF gene is a genetic risk factor for schizophrenia and is related to the chlorpromazine-induced extrapyramidal syndrome in the Chinese population. *Pharmacogenet Genomics* 2008; **18**(6)**:** 449-457.

16. Sodersten K, Palsson E, Ishima T, Funa K, Landen M, Hashimoto K*, et al*. Abnormality in serum levels of mature brain-derived neurotrophic factor (BDNF) and its precursor proBDNF in mood-stabilized patients with bipolar disorder: a study of two independent cohorts. *J Affect Disord* 2014; **160:** 1-9.

17. Dell'Osso B, D'Addario C, Carlotta Palazzo M, Benatti B, Camuri G, Galimberti D*, et al*. Epigenetic modulation of BDNF gene: differences in DNA methylation between unipolar and bipolar patients. *J Affect Disord* 2014; **166:** 330-333.

18. Yoo HJ, Yang SY, Cho IH, Park M, Kim SA. Polymorphisms of BDNF gene and autism spectrum disorders: family based association study with korean trios. *Psychiatry Investig* 2014; **11**(3)**:** 319-324.

19. Raznahan A, Toro R, Proitsi P, Powell J, Paus T, P FB*, et al*. A functional polymorphism of the brain derived neurotrophic factor gene and cortical anatomy in autism spectrum disorder. *J Neurodev Disord* 2009; **1**(3)**:** 215-223.

20. Hashimoto K, Iwata Y, Nakamura K, Tsujii M, Tsuchiya KJ, Sekine Y*, et al*. Reduced serum levels of brain-derived neurotrophic factor in adult male patients with autism. *Prog Neuropsychopharmacol Biol Psychiatry* 2006; **30**(8)**:** 1529-1531.

21. Shimizu E, Hashimoto K, Okamura N, Koike K, Komatsu N, Kumakiri C*, et al*. Alterations of serum levels of brain-derived neurotrophic factor (BDNF) in depressed patients with or without antidepressants. *Biol Psychiatry* 2003; **54**(1)**:** 70-75.

22. Molendijk ML, Spinhoven P, Polak M, Bus BA, Penninx BW, Elzinga BM. Serum BDNF concentrations as peripheral manifestations of depression: evidence from a systematic review and meta-analyses on 179 associations (N=9484). *Mol Psychiatry* 2014; **19**(7)**:** 791-800.

23. Corominas-Roso M, Roncero C, Eiroa-Orosa FJ, Gonzalvo B, Grau-Lopez L, Ribases M*, et al*. Brain-derived neurotrophic factor serum levels in cocaine-dependent patients during early abstinence. *Eur Neuropsychopharmacol* 2013; **23**(9)**:** 1078-1084.

24. Kim DJ, Roh S, Kim Y, Yoon SJ, Lee HK, Han CS*, et al*. High concentrations of plasma brain-derived neurotrophic factor in methamphetamine users. *Neurosci Lett* 2005; **388**(2)**:** 112-115.

25. Ghitza UE, Zhai H, Wu P, Airavaara M, Shaham Y, Lu L. Role of BDNF and GDNF in drug reward and relapse: a review. *Neurosci Biobehav Rev* 2010; **35**(2)**:** 157-171.

26. Xu X, Mill J, Zhou K, Brookes K, Chen CK, Asherson P. Family-based association study between brain-derived neurotrophic factor gene polymorphisms and attention deficit hyperactivity disorder in UK and Taiwanese samples. *Am J Med Genet B Neuropsychiatr Genet* 2007; **144B**(1)**:** 83-86.

27. Ribases M, Hervas A, Ramos-Quiroga JA, Bosch R, Bielsa A, Gastaminza X*, et al*. Association study of 10 genes encoding neurotrophic factors and their receptors in adult and child attention-deficit/hyperactivity disorder. *Biol Psychiatry* 2008; **63**(10)**:** 935-945.

28. Reuter M, Kirsch P, Hennig J. Inferring candidate genes for attention deficit hyperactivity disorder (ADHD) assessed by the World Health Organization Adult ADHD Self-Report Scale (ASRS). *J Neural Transm (Vienna)* 2006; **113**(7)**:** 929-938.

29. Gothelf D, Michaelovsky E, Frisch A, Zohar AH, Presburger G, Burg M*, et al*. Association of the low-activity COMT 158Met allele with ADHD and OCD in subjects with velocardiofacial syndrome. *Int J Neuropsychopharmacol* 2007; **10**(3)**:** 301-308.

30. Al-Asmary S, Kadasah S, Arfin M, Tariq M, Al-Asmari A. Genetic association of catechol-O-methyltransferase val(158)met polymorphism in Saudi schizophrenia patients. *Genet Mol Res* 2014; **13**(2)**:** 3079-3088.

31. Glatt SJ, Faraone SV, Tsuang MT. Association between a functional catechol O-methyltransferase gene polymorphism and schizophrenia: meta-analysis of case-control and family-based studies. *Am J Psychiatry* 2003; **160**(3)**:** 469-476.

32. Pandolfo G, Gugliandolo A, Gangemi C, Arrigo R, Curro M, La Ciura G*, et al*. Association of the COMT synonymous polymorphism Leu136Leu and missense variant Val158Met with mood disorders. *J Affect Disord* 2015; **177:** 108-113.

33. Massat I, Kocabas NA, Crisafulli C, Chiesa A, Calati R, Linotte S*, et al*. COMT and age at onset in mood disorders: a replication and extension study. *Neurosci Lett* 2011; **498**(3)**:** 218-221.

34. Yoo HJ, Cho IH, Park M, Yang SY, Kim SA. Association of the catechol-o-methyltransferase gene polymorphisms with Korean autism spectrum disorders. *J Korean Med Sci* 2013; **28**(9)**:** 1403-1406.

35. Guo T, Wang W, Liu B, Chen H, Yang C. Catechol-O-methyltransferase Val158Met polymorphism and risk of autism spectrum disorders. *J Int Med Res* 2013; **41**(3)**:** 725-734.

36. Munafo MR, Johnstone EC, Guo B, Murphy MF, Aveyard P. Association of COMT Val108/158Met genotype with smoking cessation. *Pharmacogenet Genomics* 2008; **18**(2)**:** 121-128.

37. Schellekens AF, Franke B, Ellenbroek B, Cools A, de Jong CA, Buitelaar JK*, et al*. Reduced dopamine receptor sensitivity as an intermediate phenotype in alcohol dependence and the role of the COMT Val158Met and DRD2 Taq1A genotypes. *Arch Gen Psychiatry* 2003; **69**(4)**:** 339-348.

38. Cubells JF, Sun X, Li W, Bonsall RW, McGrath JA, Avramopoulos D*, et al*. Linkage analysis of plasma dopamine beta-hydroxylase activity in families of patients with schizophrenia. *Hum Genet* 2011; **130**(5)**:** 635-643.

39. Ates O, Celikel FC, Taycan SE, Sezer S, Karakus N. Association between 1603C>T polymorphism of DBH gene and bipolar disorder in a Turkish population. *Gene* 2013; **519**(2)**:** 356-359.

40. Preuss UW, Wurst FM, Ridinger M, Rujescu D, Fehr C, Koller G*, et al*. Association of functional DBH genetic variants with alcohol dependence risk and related depression and suicide attempt phenotypes: results from a large multicenter association study. *Drug Alcohol Depend* 2013; **133**(2)**:** 459-467.

41. Zhou Y, Wang J, He Y, Zhou J, Xi Q, Song X*, et al*. Association between dopamine beta-hydroxylase 19-bp insertion/deletion polymorphism and major depressive disorder. *J Mol Neurosci* 2015; **55**(2)**:** 367-371.

42. Freire MT, Marques FZ, Hutz MH, Bau CH. Polymorphisms in the DBH and DRD2 gene regions and smoking behavior. *Eur Arch Psychiatry Clin Neurosci* 2006; **256**(2)**:** 93-97.

43. Daly G, Hawi Z, Fitzgerald M, Gill M. Mapping susceptibility loci in attention deficit hyperactivity disorder: preferential transmission of parental alleles at DAT1, DBH and DRD5 to affected children. *Mol Psychiatry* 1999; **4**(2)**:** 192-196.

44. Roman T, Schmitz M, Polanczyk GV, Eizirik M, Rohde LA, Hutz MH. Further evidence for the association between attention-deficit/hyperactivity disorder and the dopamine-beta-hydroxylase gene. *Am J Med Genet* 2002; **114**(2)**:** 154-158.

45. Ribases M, Ramos-Quiroga JA, Hervas A, Bosch R, Bielsa A, Gastaminza X*, et al*. Exploration of 19 serotoninergic candidate genes in adults and children with attention-deficit/hyperactivity disorder identifies association for 5HT2A, DDC and MAOB. *Mol Psychiatry* 2009; **14**(1)**:** 71-85.

46. Borglum AD, Hampson M, Kjeldsen TE, Muir W, Murray V, Ewald H*, et al*. Dopa decarboxylase genotypes may influence age at onset of schizophrenia. *Mol Psychiatry* 2001; **6**(6)**:** 712-717.

47. Borglum AD, Bruun TG, Kjeldsen TE, Ewald H, Mors O, Kirov G*, et al*. Two novel variants in the DOPA decarboxylase gene: association with bipolar affective disorder. *Mol Psychiatry* 1999; **4**(6)**:** 545-551.

48. Borglum AD, Kirov G, Craddock N, Mors O, Muir W, Murray V*, et al*. Possible parent-of-origin effect of Dopa decarboxylase in susceptibility to bipolar affective disorder. *Am J Med Genet B Neuropsychiatr Genet* 2003; **117B**(1)**:** 18-22.

49. Toma C, Hervas A, Balmana N, Salgado M, Maristany M, Vilella E*, et al*. Neurotransmitter systems and neurotrophic factors in autism: association study of 37 genes suggests involvement of DDC. *World J Biol Psychiatry* 2013; **14**(7)**:** 516-527.

50. Ma JZ, Beuten J, Payne TJ, Dupont RT, Elston RC, Li MD. Haplotype analysis indicates an association between the DOPA decarboxylase (DDC) gene and nicotine dependence. *Hum Mol Genet* 2005; **14**(12)**:** 1691-1698.

51. Comings DE, Comings BG, Muhleman D, Dietz G, Shahbahrami B, Tast D*, et al*. The dopamine D2 receptor locus as a modifying gene in neuropsychiatric disorders. *Jama* 1991; **266**(13)**:** 1793-1800.

52. Comings DE, Wu S, Chiu C, Ring RH, Gade R, Ahn C*, et al*. Polygenic inheritance of Tourette syndrome, stuttering, attention deficit hyperactivity, conduct, and oppositional defiant disorder: the additive and subtractive effect of the three dopaminergic genes--DRD2, D beta H, and DAT1. *Am J Med Genet* 1996; **67**(3)**:** 264-288.

53. Sery O, Drtilkova I, Theiner P, Pitelova R, Staif R, Znojil V*, et al*. Polymorphism of DRD2 gene and ADHD. *Neuro Endocrinol Lett* 2006; **27**(1-2)**:** 236-240.

54. Liu L, Fan D, Ding N, Hu Y, Cai G, Wang L*, et al*. The relationship between DRD2 gene polymorphisms (C957T and C939T) and schizophrenia: a meta-analysis. *Neurosci Lett* 2014; **583:** 43-48.

55. Kaalund SS, Newburn EN, Ye T, Tao R, Li C, Deep-Soboslay A*, et al*. Contrasting changes in DRD1 and DRD2 splice variant expression in schizophrenia and affective disorders, and associations with SNPs in postmortem brain. *Mol Psychiatry* 2014; **19**(12)**:** 1258-1266.

56. Zou YF, Wang F, Feng XL, Li WF, Tian YH, Tao JH*, et al*. Association of DRD2 gene polymorphisms with mood disorders: a meta-analysis. *J Affect Disord* 2010; **136**(3)**:** 229-237.

57. Massat I, Souery D, Del-Favero J, Van Gestel S, Serretti A, Macciardi F*, et al*. Positive association of dopamine D2 receptor polymorphism with bipolar affective disorder in a European Multicenter Association Study of affective disorders. *Am J Med Genet* 2002; **114**(2)**:** 177-185.

58. Li T, Liu X, Sham PC, Aitchison KJ, Cai G, Arranz MJ*, et al*. Association analysis between dopamine receptor genes and bipolar affective disorder. *Psychiatry Res* 1999; **86**(3)**:** 193-201.

59. Hettinger JA, Liu X, Hudson ML, Lee A, Cohen IL, Michaelis RC*, et al*. DRD2 and PPP1R1B (DARPP-32) polymorphisms independently confer increased risk for autism spectrum disorders and additively predict affected status in male-only affected sib-pair families. *Behav Brain Funct* 2012; **8:** 19.

60. Salem AM, Ismail S, Zarouk WA, Abdul Baky O, Sayed AA, Abd El-Hamid S*, et al*. Genetic variants of neurotransmitter-related genes and miRNAs in Egyptian autistic patients. *ScientificWorldJournal* 2013; **2013:** 670621.

61. Arcos-Burgos M, Castellanos FX, Konecki D, Lopera F, Pineda D, Palacio JD*, et al*. Pedigree disequilibrium test (PDT) replicates association and linkage between DRD4 and ADHD in multigenerational and extended pedigrees from a genetic isolate. *Mol Psychiatry* 2004; **9**(3)**:** 252-259.

62. Faraone SV, Perlis RH, Doyle AE, Smoller JW, Goralnick JJ, Holmgren MA*, et al*. Molecular genetics of attention-deficit/hyperactivity disorder. *Biol Psychiatry* 2005; **57**(11)**:** 1313-1323.

63. Li D, Sham PC, Owen MJ, He L. Meta-analysis shows significant association between dopamine system genes and attention deficit hyperactivity disorder (ADHD). *Hum Mol Genet* 2006; **15**(14)**:** 2276-2284.

64. Gizer IR, Ficks C, Waldman ID. Candidate gene studies of ADHD: a meta-analytic review. *Hum Genet* 2009; **126**(1)**:** 51-90.

65. Lung FW, Shu BC, Kao WT, Chen CN, Ku YC, Tzeng DS. Association of DRD4 uVNTR and TP53 codon 72 polymorphisms with schizophrenia: a case-control study. *BMC Med Genet* 2009; **10:** 147.

66. Lai JH, Zhu YS, Huo ZH, Sun RF, Yu B, Wang YP*, et al*. Association study of polymorphisms in the promoter region of DRD4 with schizophrenia, depression, and heroin addiction. *Brain Res* 2010; **1359:** 227-232.

67. Kordi-Tamandani DM, Sahranavard R, Torkamanzehi A. Analysis of association between dopamine receptor genes' methylation and their expression profile with the risk of schizophrenia. *Psychiatr Genet* 2013; **23**(5)**:** 183-187.

68. Goncalves VF, Tiwari AK, de Luca V, Kong SL, Zai C, Tampakeras M*, et al*. DRD4 VNTR polymorphism and age at onset of severe mental illnesses. *Neurosci Lett* 2012; **519**(1)**:** 9-13.

69. Reiersen AM, Todorov AA. Association between DRD4 genotype and Autistic Symptoms in DSM-IV ADHD. *J Can Acad Child Adolesc Psychiatry* 2011; **20**(1)**:** 15-21.

70. Lopez Leon S, Croes EA, Sayed-Tabatabaei FA, Claes S, Van Broeckhoven C, van Duijn CM. The dopamine D4 receptor gene 48-base-pair-repeat polymorphism and mood disorders: a meta-analysis. *Biol Psychiatry* 2005; **57**(9)**:** 999-1003.

71. Chen D, Liu F, Shang Q, Song X, Miao X, Wang Z. Association between polymorphisms of DRD2 and DRD4 and opioid dependence: evidence from the current studies. *Am J Med Genet B Neuropsychiatr Genet* 2011; **156B**(6)**:** 661-670.

72. van der Zwaluw CS, Larsen H, Engels RC. Best friends and alcohol use in adolescence: the role of the dopamine D4 receptor gene. *Addict Biol* 2012; **17**(6)**:** 1036-1045.

73. McCracken JT, Smalley SL, McGough JJ, Crawford L, Del'Homme M, Cantor RM*, et al*. Evidence for linkage of a tandem duplication polymorphism upstream of the dopamine D4 receptor gene (DRD4) with attention deficit hyperactivity disorder (ADHD). *Mol Psychiatry* 2000; **5**(5)**:** 531-536.

74. Kustanovich V, Ishii J, Crawford L, Yang M, McGough JJ, McCracken JT*, et al*. Transmission disequilibrium testing of dopamine-related candidate gene polymorphisms in ADHD: confirmation of association of ADHD with DRD4 and DRD5. *Mol Psychiatry* 2004; **9**(7)**:** 711-717.

75. Bellgrove MA, Hawi Z, Lowe N, Kirley A, Robertson IH, Gill M. DRD4 gene variants and sustained attention in attention deficit hyperactivity disorder (ADHD): effects of associated alleles at the VNTR and -521 SNP. *Am J Med Genet B Neuropsychiatr Genet* 2005; **136B**(1)**:** 81-86.

76. Lowe N, Kirley A, Mullins C, Fitzgerald M, Gill M, Hawi Z. Multiple marker analysis at the promoter region of the DRD4 gene and ADHD: evidence of linkage and association with the SNP -616. *Am J Med Genet B Neuropsychiatr Genet* 2004; **131B**(1)**:** 33-37.

77. Tahir E, Yazgan Y, Cirakoglu B, Ozbay F, Waldman I, Asherson PJ. Association and linkage of DRD4 and DRD5 with attention deficit hyperactivity disorder (ADHD) in a sample of Turkish children. *Mol Psychiatry* 2000; **5**(4)**:** 396-404.

78. Hawi Z, Lowe N, Kirley A, Gruenhage F, Nothen M, Greenwood T*, et al*. Linkage disequilibrium mapping at DAT1, DRD5 and DBH narrows the search for ADHD susceptibility alleles at these loci. *Mol Psychiatry* 2003; **8**(3)**:** 299-308.

79. Lowe N, Kirley A, Hawi Z, Sham P, Wickham H, Kratochvil CJ*, et al*. Joint analysis of the DRD5 marker concludes association with attention-deficit/hyperactivity disorder confined to the predominantly inattentive and combined subtypes. *Am J Hum Genet* 2004; **74**(2)**:** 348-356.

80. Zhao Y, Ding M, Pang H, Xu XM, Wang BJ. Relationship between genetic polymorphisms in the DRD5 gene and paranoid schizophrenia in northern Han Chinese. *Genet Mol Res* 2014; **13**(1)**:** 1609-1618.

81. Muir WJ, Thomson ML, McKeon P, Mynett-Johnson L, Whitton C, Evans KL*, et al*. Markers close to the dopamine D5 receptor gene (DRD5) show significant association with schizophrenia but not bipolar disorder. *Am J Med Genet* 2001; **105**(2)**:** 152-158.

82. Taurines R, Grunblatt E, Schecklmann M, Schwenck C, Albantakis L, Reefschlager L*, et al*. Altered mRNA expression of monoaminergic candidate genes in the blood of children with attention deficit hyperactivity disorder and autism spectrum disorder. *World J Biol Psychiatry* 2011; **12 Suppl 1:** 104-108.

83. Turic D, Langley K, Mills S, Stephens M, Lawson D, Govan C*, et al*. Follow-up of genetic linkage findings on chromosome 16p13: evidence of association of N-methyl-D aspartate glutamate receptor 2A gene polymorphism with ADHD. *Mol Psychiatry* 2004; **9**(2)**:** 169-173.

84. Tang J, Chen X, Xu X, Wu R, Zhao J, Hu Z*, et al*. Significant linkage and association between a functional (GT)n polymorphism in promoter of the N-methyl-D-aspartate receptor subunit gene (GRIN2A) and schizophrenia. *Neurosci Lett* 2006; **409**(1)**:** 80-82.

85. Itokawa M, Yamada K, Yoshitsugu K, Toyota T, Suga T, Ohba H*, et al*. A microsatellite repeat in the promoter of the N-methyl-D-aspartate receptor 2A subunit (GRIN2A) gene suppresses transcriptional activity and correlates with chronic outcome in schizophrenia. *Pharmacogenetics* 2003; **13**(5)**:** 271-278.

86. Itokawa M, Yamada K, Iwayama-Shigeno Y, Ishitsuka Y, Detera-Wadleigh S, Yoshikawa T. Genetic analysis of a functional GRIN2A promoter (GT)n repeat in bipolar disorder pedigrees in humans. *Neurosci Lett* 2003; **345**(1)**:** 53-56.

87. Barnby G, Abbott A, Sykes N, Morris A, Weeks DE, Mott R*, et al*. Candidate-gene screening and association analysis at the autism-susceptibility locus on chromosome 16p: evidence of association at GRIN2A and ABAT. *Am J Hum Genet* 2005; **76**(6)**:** 950-966.

88. Yoo HJ, Cho IH, Park M, Yang SY, Kim SA. Family based association of GRIN2A and GRIN2B with Korean autism spectrum disorders. *Neurosci Lett* 2012; **512**(2)**:** 89-93.

89. Kaut O, Schmitt I, Hofmann A, Hoffmann P, Schlaepfer TE, Wullner U*, et al*. Aberrant NMDA receptor DNA methylation detected by epigenome-wide analysis of hippocampus and prefrontal cortex in major depression. *Eur Arch Psychiatry Clin Neurosci* 2015; **265**(4)**:** 331-341.

90. Zhao B, Zhu Y, Wang W, Cui HM, Wang YP, Lai JH. Analysis of variations in the glutamate receptor, N-methyl D-aspartate 2A (GRIN2A) gene reveals their relative importance as genetic susceptibility factors for heroin addiction. *PLoS One* 2013; **8**(8)**:** e70817.

91. Domart MC, Benyamina A, Lemoine A, Bourgain C, Blecha L, Debuire B*, et al*. Association between a polymorphism in the promoter of a glutamate receptor subunit gene (GRIN2A) and alcoholism. *Addict Biol* 2012; **17**(4)**:** 783-785.

92. Zhou X, Ding M, Ding C, Yao J, Pang H, Xing J*, et al*. Relationship between genetic polymorphisms in the HTR1A gene and paranoid schizophrenia in a northern Han Chinese population. *J Mol Neurosci* 2013; **49**(3)**:** 625-631.

93. Tang H, Dalton CF, Srisawat U, Zhang ZJ, Reynolds GP. Methylation at a transcription factor-binding site on the 5-HT1A receptor gene correlates with negative symptom treatment response in first episode schizophrenia. *Int J Neuropsychopharmacol* 2014; **17**(4)**:** 645-649.

94. Kishi T, Okochi T, Tsunoka T, Okumura T, Kitajima T, Kawashima K*, et al*. Serotonin 1A receptor gene, schizophrenia and bipolar disorder: an association study and meta-analysis. *Psychiatry Res* 2011; **185**(1-2)**:** 20-26.

95. Kishi T, Yoshimura R, Fukuo Y, Okochi T, Matsunaga S, Umene-Nakano W*, et al*. The serotonin 1A receptor gene confer susceptibility to mood disorders: results from an extended meta-analysis of patients with major depression and bipolar disorder. *Eur Arch Psychiatry Clin Neurosci* 2012; **263**(2)**:** 105-118.

96. Sener EF, Cikili Uytun M, Korkmaz Bayramov K, Zararsiz G, Oztop DB, Canatan H*, et al*. The roles of CC2D1A and HTR1A gene expressions in autism spectrum disorders. *Metab Brain Dis* 2016.

97. Zuo L, Zhang XY, Wang F, Li CS, Lu L, Ye L*, et al*. Genome-wide significant association signals in IPO11-HTR1A region specific for alcohol and nicotine codependence. *Alcohol Clin Exp Res* 2013; **37**(5)**:** 730-739.

98. Ghadirivasfi M, Nohesara S, Ahmadkhaniha HR, Eskandari MR, Mostafavi S, Thiagalingam S*, et al*. Hypomethylation of the serotonin receptor type-2A Gene (HTR2A) at T102C polymorphic site in DNA derived from the saliva of patients with schizophrenia and bipolar disorder. *Am J Med Genet B Neuropsychiatr Genet* 2011; **156B**(5)**:** 536-545.

99. Sujitha SP, Nair A, Banerjee M, Lakshmanan S, Harshavaradhan S, Gunasekaran S*, et al*. 5-Hydroxytryptamine (serotonin) 2A receptor gene polymorphism is associated with schizophrenia. *Indian J Med Res* 2014; **140**(6)**:** 736-743.

100. Tan J, Chen S, Su L, Long J, Xie J, Shen T*, et al*. Association of the T102C polymorphism in the HTR2A gene with major depressive disorder, bipolar disorder, and schizophrenia. *Am J Med Genet B Neuropsychiatr Genet* 2014; **165B**(5)**:** 438-455.

101. McAuley EZ, Fullerton JM, Blair IP, Donald JA, Mitchell PB, Schofield PR. Association between the serotonin 2A receptor gene and bipolar affective disorder in an Australian cohort. *Psychiatr Genet* 2009; **19**(5)**:** 244-252.

102. Xiang B, Yang Z, Lin Y, Guan L, Li X, Deng W*, et al*. Genes in the serotonin pathway are associated with bipolar affective disorder in a Han Chinese population. *Neurosci Bull* 2014; **30**(1)**:** 33-42.

103. Smith RM, Banks W, Hansen E, Sadee W, Herman GE. Family-based clinical associations and functional characterization of the serotonin 2A receptor gene (HTR2A) in autism spectrum disorder. *Autism Res* 2014; **7**(4)**:** 459-467.

104. Nyffeler J, Walitza S, Bobrowski E, Gundelfinger R, Grunblatt E. Association study in siblings and case-controls of serotonin- and oxytocin-related genes with high functioning autism. *J Mol Psychiatry* 2014; **2**(1)**:** 1.

105. Du L, Bakish D, Lapierre YD, Ravindran AV, Hrdina PD. Association of polymorphism of serotonin 2A receptor gene with suicidal ideation in major depressive disorder. *Am J Med Genet* 2000; **96**(1)**:** 56-60.

106. Cao J, Liu X, Han S, Zhang CK, Liu Z, Li D. Association of the HTR2A gene with alcohol and heroin abuse. *Hum Genet* 2014; **133**(3)**:** 357-365.

107. Wrzosek M, Jakubczyk A, Wrzosek M, Matsumoto H, Lukaszkiewicz J, Brower KJ*, et al*. Serotonin 2A receptor gene (HTR2A) polymorphism in alcohol-dependent patients. *Pharmacol Rep* 2012; **64**(2)**:** 449-453.

108. Levitan RD, Masellis M, Basile VS, Lam RW, Jain U, Kaplan AS*, et al*. Polymorphism of the serotonin-2A receptor gene (HTR2A) associated with childhood attention deficit hyperactivity disorder (ADHD) in adult women with seasonal affective disorder. *J Affect Disord* 2002; **71**(1-3)**:** 229-233.

109. Guimaraes AP, Zeni C, Polanczyk GV, Genro JP, Roman T, Rohde LA*, et al*. Serotonin genes and attention deficit/hyperactivity disorder in a Brazilian sample: preferential transmission of the HTR2A 452His allele to affected boys. *Am J Med Genet B Neuropsychiatr Genet* 2007; **144B**(1)**:** 69-73.

110. Hawi Z, Dring M, Kirley A, Foley D, Kent L, Craddock N*, et al*. Serotonergic system and attention deficit hyperactivity disorder (ADHD): a potential susceptibility locus at the 5-HT(1B) receptor gene in 273 nuclear families from a multi-centre sample. *Mol Psychiatry* 2002; **7**(7)**:** 718-725.

111. Quist JF, Barr CL, Schachar R, Roberts W, Malone M, Tannock R*, et al*. The serotonin 5-HT1B receptor gene and attention deficit hyperactivity disorder. *Mol Psychiatry* 2003; **8**(1)**:** 98-102.

112. Smoller JW, Biederman J, Arbeitman L, Doyle AE, Fagerness J, Perlis RH*, et al*. Association between the 5HT1B receptor gene (HTR1B) and the inattentive subtype of ADHD. *Biol Psychiatry* 2006; **59**(5)**:** 460-467.

113. Huang YY, Oquendo MA, Friedman JM, Greenhill LL, Brodsky B, Malone KM*, et al*. Substance abuse disorder and major depression are associated with the human 5-HT1B receptor gene (HTR1B) G861C polymorphism. *Neuropsychopharmacology* 2003; **28**(1)**:** 163-169.

114. Rocha BA, Scearce-Levie K, Lucas JJ, Hiroi N, Castanon N, Crabbe JC*, et al*. Increased vulnerability to cocaine in mice lacking the serotonin-1B receptor. *Nature* 1998; **393**(6681)**:** 175-178.

115. Li J, Wang Y, Zhou R, Zhang H, Yang L, Wang B*, et al*. Association between polymorphisms in serotonin 2C receptor gene and attention-deficit/hyperactivity disorder in Han Chinese subjects. *Neurosci Lett* 2006; **407**(2)**:** 107-111.

116. Xu X, Brookes K, Sun B, Ilott N, Asherson P. Investigation of the serotonin 2C receptor gene in attention deficit hyperactivity disorder in UK samples. *BMC Res Notes* 2009; **2:** 71.

117. Koks S, Nikopensius T, Koido K, Maron E, Altmae S, Heinaste E*, et al*. Analysis of SNP profiles in patients with major depressive disorder. *Int J Neuropsychopharmacol* 2006; **9**(2)**:** 167-174.

118. Yasseen B, Kennedy JL, Zawertailo LA, Busto UE. Comorbidity between bipolar disorder and alcohol use disorder: association of dopamine and serotonin gene polymorphisms. *Psychiatry Res* 2010; **176**(1)**:** 30-33.

119. Anastasio NC, Liu S, Maili L, Swinford SE, Lane SD, Fox RG*, et al*. Variation within the serotonin (5-HT) 5-HT(2)C receptor system aligns with vulnerability to cocaine cue reactivity. *Transl Psychiatry* 2014; **4:** e369.

120. Arcos-Burgos M, Jain M, Acosta MT, Shively S, Stanescu H, Wallis D*, et al*. A common variant of the latrophilin 3 gene, LPHN3, confers susceptibility to ADHD and predicts effectiveness of stimulant medication. *Mol Psychiatry* 2010; **15**(11)**:** 1053-1066.

121. Ribases M, Ramos-Quiroga JA, Sanchez-Mora C, Bosch R, Richarte V, Palomar G*, et al*. Contribution of LPHN3 to the genetic susceptibility to ADHD in adulthood: a replication study. *Genes Brain Behav* 2010; **10**(2)**:** 149-157.

122. Manor I, Tyano S, Mel E, Eisenberg J, Bachner-Melman R, Kotler M*, et al*. Family-based and association studies of monoamine oxidase A and attention deficit hyperactivity disorder (ADHD): preferential transmission of the long promoter-region repeat and its association with impaired performance on a continuous performance test (TOVA). *Mol Psychiatry* 2002; **7**(6)**:** 626-632.

123. Qiu HT, Meng HQ, Song C, Xiu MH, Chen da C, Zhu FY*, et al*. Association between monoamine oxidase (MAO)-A gene variants and schizophrenia in a Chinese population. *Brain Res* 2009; **1287:** 67-73.

124. Jonsson EG, Norton N, Forslund K, Mattila-Evenden M, Rylander G, Asberg M*, et al*. Association between a promoter variant in the monoamine oxidase A gene and schizophrenia. *Schizophr Res* 2003; **61**(1)**:** 31-37.

125. Preisig M, Bellivier F, Fenton BT, Baud P, Berney A, Courtet P*, et al*. Association between bipolar disorder and monoamine oxidase A gene polymorphisms: results of a multicenter study. *Am J Psychiatry* 2000; **157**(6)**:** 948-955.

126. Eslami Amirabadi MR, Rajezi Esfahani S, Davari-Ashtiani R, Khademi M, Emamalizadeh B, Movafagh A*, et al*. Monoamine oxidase a gene polymorphisms and bipolar disorder in Iranian population. *Iran Red Crescent Med J* 2015; **17**(2)**:** e23095.

127. Cohen IL, Liu X, Schutz C, White BN, Jenkins EC, Brown WT*, et al*. Association of autism severity with a monoamine oxidase A functional polymorphism. *Clin Genet* 2003; **64**(3)**:** 190-197.

128. Yoo HJ, Lee SK, Park M, Cho IH, Hyun SH, Lee JC*, et al*. Family- and population-based association studies of monoamine oxidase A and autism spectrum disorders in Korean. *Neurosci Res* 2009; **63**(3)**:** 172-176.

129. Bortolato M, Godar SC, Alzghoul L, Zhang J, Darling RD, Simpson KL*, et al*. Monoamine oxidase A and A/B knockout mice display autistic-like features. *Int J Neuropsychopharmacol* 2012; **16**(4)**:** 869-888.

130. Dannlowski U, Ohrmann P, Konrad C, Domschke K, Bauer J, Kugel H*, et al*. Reduced amygdala-prefrontal coupling in major depression: association with MAOA genotype and illness severity. *Int J Neuropsychopharmacol* 2009; **12**(1)**:** 11-22.

131. Lung FW, Tzeng DS, Huang MF, Lee MB. Association of the MAOA promoter uVNTR polymorphism with suicide attempts in patients with major depressive disorder. *BMC Med Genet* 2011; **12:** 74.

132. Philibert RA, Gunter TD, Beach SR, Brody GH, Madan A. MAOA methylation is associated with nicotine and alcohol dependence in women. *Am J Med Genet B Neuropsychiatr Genet* 2008; **147B**(5)**:** 565-570.

133. Nilsson KW, Comasco E, Aslund C, Nordquist N, Leppert J, Oreland L. MAOA genotype, family relations and sexual abuse in relation to adolescent alcohol consumption. *Addict Biol* 2011; **16**(2)**:** 347-355.

134. Jiang S, Xin R, Lin S, Qian Y, Tang G, Wang D*, et al*. Linkage studies between attention-deficit hyperactivity disorder and the monoamine oxidase genes. *Am J Med Genet* 2001; **105**(8)**:** 783-788.

135. Domschke K, Sheehan K, Lowe N, Kirley A, Mullins C, O'Sullivan R*, et al*. Association analysis of the monoamine oxidase A and B genes with attention deficit hyperactivity disorder (ADHD) in an Irish sample: preferential transmission of the MAO-A 941G allele to affected children. *Am J Med Genet B Neuropsychiatr Genet* 2005; **134B**(1)**:** 110-114.

136. Xu X, Brookes K, Chen CK, Huang YS, Wu YY, Asherson P. Association study between the monoamine oxidase A gene and attention deficit hyperactivity disorder in Taiwanese samples. *BMC Psychiatry* 2007; **7:** 10.

137. Brookes K, Xu X, Chen W, Zhou K, Neale B, Lowe N*, et al*. The analysis of 51 genes in DSM-IV combined type attention deficit hyperactivity disorder: association signals in DRD4, DAT1 and 16 other genes. *Mol Psychiatry* 2006; **11**(10)**:** 934-953.

138. Carrera N, Sanjuan J, Molto MD, Carracedo A, Costas J. Recent adaptive selection at MAOB and ancestral susceptibility to schizophrenia. *Am J Med Genet B Neuropsychiatr Genet* 2009; **150B**(3)**:** 369-374.

139. Sun J, Jayathilake K, Zhao Z, Meltzer HY. Investigating association of four gene regions (GABRB3, MAOB, PAH, and SLC6A4) with five symptoms in schizophrenia. *Psychiatry Res* 2012; **198**(2)**:** 202-206.

140. Lin S, Jiang S, Wu X, Qian Y, Wang D, Tang G*, et al*. Association analysis between mood disorder and monoamine oxidase gene. *Am J Med Genet* 2000; **96**(1)**:** 12-14.

141. Launay JM, Del Pino M, Chironi G, Callebert J, Peoc'h K, Megnien JL*, et al*. Smoking induces long-lasting effects through a monoamine-oxidase epigenetic regulation. *PLoS One* 2009; **4**(11)**:** e7959.

142. Ho MC, Cherng CG, Tsai YP, Chiang CY, Chuang JY, Kao SF*, et al*. Chronic treatment with monoamine oxidase-B inhibitors decreases cocaine reward in mice. *Psychopharmacology (Berl)* 2009; **205**(1)**:** 141-149.

143. Durany N, Michel T, Zochling R, Boissl KW, Cruz-Sanchez FF, Riederer P*, et al*. Brain-derived neurotrophic factor and neurotrophin 3 in schizophrenic psychoses. *Schizophr Res* 2001; **52**(1-2)**:** 79-86.

144. Virgos C, Martorell L, Valero J, Figuera L, Civeira F, Joven J*, et al*. Association study of schizophrenia with polymorphisms at six candidate genes. *Schizophr Res* 2001; **49**(1-2)**:** 65-71.

145. Fernandes BS, Gama CS, Walz JC, Cereser KM, Fries GR, Colpo G*, et al*. Increased neurotrophin-3 in drug-free subjects with bipolar disorder during manic and depressive episodes. *J Psychiatr Res* 2010; **44**(9)**:** 561-565.

146. Loch AA, Zanetti MV, de Sousa RT, Chaim TM, Serpa MH, Gattaz WF*, et al*. Elevated neurotrophin-3 and neurotrophin 4/5 levels in unmedicated bipolar depression and the effects of lithium. *Prog Neuropsychopharmacol Biol Psychiatry* 2015; **56:** 243-246.

147. Sajdel-Sulkowska EM, Xu M, Koibuchi N. Increase in cerebellar neurotrophin-3 and oxidative stress markers in autism. *Cerebellum* 2009; **8**(3)**:** 366-372.

148. Tostes MH, Teixeira HC, Gattaz WF, Brandao MA, Raposo NR. Altered neurotrophin, neuropeptide, cytokines and nitric oxide levels in autism. *Pharmacopsychiatry* 2012; **45**(6)**:** 241-243.

149. Hock C, Heese K, Muller-Spahn F, Huber P, Riesen W, Nitsch RM*, et al*. Increased cerebrospinal fluid levels of neurotrophin 3 (NT-3) in elderly patients with major depression. *Mol Psychiatry* 2000; **5**(5)**:** 510-513.

150. Otsuki K, Uchida S, Watanuki T, Wakabayashi Y, Fujimoto M, Matsubara T*, et al*. Altered expression of neurotrophic factors in patients with major depression. *J Psychiatr Res* 2008; **42**(14)**:** 1145-1153.

151. Pierce RC, Pierce-Bancroft AF, Prasad BM. Neurotrophin-3 contributes to the initiation of behavioral sensitization to cocaine by activating the Ras/Mitogen-activated protein kinase signal transduction cascade. *J Neurosci* 1999; **19**(19)**:** 8685-8695.

152. Akbarian S, Bates B, Liu RJ, Skirboll SL, Pejchal T, Coppola V*, et al*. Neurotrophin-3 modulates noradrenergic neuron function and opiate withdrawal. *Mol Psychiatry* 2001; **6**(5)**:** 593-604.

153. Hashimoto T, Bergen SE, Nguyen QL, Xu B, Monteggia LM, Pierri JN*, et al*. Relationship of brain-derived neurotrophic factor and its receptor TrkB to altered inhibitory prefrontal circuitry in schizophrenia. *J Neurosci* 2005; **25**(2)**:** 372-383.

154. Ray MT, Shannon Weickert C, Webster MJ. Decreased BDNF and TrkB mRNA expression in multiple cortical areas of patients with schizophrenia and mood disorders. *Transl Psychiatry* 2014; **4:** e389.

155. Soontornniyomkij B, Everall IP, Chana G, Tsuang MT, Achim CL, Soontornniyomkij V. Tyrosine kinase B protein expression is reduced in the cerebellum of patients with bipolar disorder. *J Affect Disord* 2011; **133**(3)**:** 646-654.

156. Correia CT, Coutinho AM, Sequeira AF, Sousa IG, Lourenco Venda L, Almeida JP*, et al*. Increased BDNF levels and NTRK2 gene association suggest a disruption of BDNF/TrkB signaling in autism. *Genes Brain Behav* 2010; **9**(7)**:** 841-848.

157. Chandley MJ, Crawford JD, Szebeni A, Szebeni K, Ordway GA. NTRK2 expression levels are reduced in laser captured pyramidal neurons from the anterior cingulate cortex in males with autism spectrum disorder. *Mol Autism* 2015; **6:** 28.

158. Murphy ML, Carballedo A, Fagan AJ, Morris D, Fahey C, Meaney J*, et al*. Neurotrophic tyrosine kinase polymorphism impacts white matter connections in patients with major depressive disorder. *Biol Psychiatry* 2012; **72**(8)**:** 663-670.

159. Xu K, Anderson TR, Neyer KM, Lamparella N, Jenkins G, Zhou Z*, et al*. Nucleotide sequence variation within the human tyrosine kinase B neurotrophin receptor gene: association with antisocial alcohol dependence. *Pharmacogenomics J* 2007; **7**(6)**:** 368-379.

160. Beuten J, Ma JZ, Payne TJ, Dupont RT, Lou XY, Crews KM*, et al*. Association of specific haplotypes of neurotrophic tyrosine kinase receptor 2 gene (NTRK2) with vulnerability to nicotine dependence in African-Americans and European-Americans. *Biol Psychiatry* 2007; **61**(1)**:** 48-55.

161. Xu X, Knight J, Brookes K, Mill J, Sham P, Craig I*, et al*. DNA pooling analysis of 21 norepinephrine transporter gene SNPs with attention deficit hyperactivity disorder: no evidence for association. *Am J Med Genet B Neuropsychiatr Genet* 2005; **134B**(1)**:** 115-118.

162. Bobb AJ, Addington AM, Sidransky E, Gornick MC, Lerch JP, Greenstein DK*, et al*. Support for association between ADHD and two candidate genes: NET1 and DRD1. *Am J Med Genet B Neuropsychiatr Genet* 2005; **134B**(1)**:** 67-72.

163. Siuta MA, Robertson SD, Kocalis H, Saunders C, Gresch PJ, Khatri V*, et al*. Dysregulation of the norepinephrine transporter sustains cortical hypodopaminergia and schizophrenia-like behaviors in neuronal rictor null mice. *PLoS Biol* 2010; **8**(6)**:** e1000393.

164. Haenisch B, Linsel K, Bruss M, Gilsbach R, Propping P, Nothen MM*, et al*. Association of major depression with rare functional variants in norepinephrine transporter and serotonin1A receptor genes. *Am J Med Genet B Neuropsychiatr Genet* 2009; **150B**(7)**:** 1013-1016.

165. Kim YK, Hwang JA, Lee HJ, Yoon HK, Ko YH, Lee BH*, et al*. Association between norepinephrine transporter gene (SLC6A2) polymorphisms and suicide in patients with major depressive disorder. *J Affect Disord* 2014; **158:** 127-132.

166. Mash DC, Ouyang Q, Qin Y, Pablo J. Norepinephrine transporter immunoblotting and radioligand binding in cocaine abusers. *J Neurosci Methods* 2005; **143**(1)**:** 79-85.

167. Kim CH, Hahn MK, Joung Y, Anderson SL, Steele AH, Mazei-Robinson MS*, et al*. A polymorphism in the norepinephrine transporter gene alters promoter activity and is associated with attention-deficit hyperactivity disorder. *Proc Natl Acad Sci U S A* 2006; **103**(50)**:** 19164-19169.

168. Cook EH, Jr., Stein MA, Krasowski MD, Cox NJ, Olkon DM, Kieffer JE*, et al*. Association of attention-deficit disorder and the dopamine transporter gene. *Am J Hum Genet* 1995; **56**(4)**:** 993-998.

169. Purper-Ouakil D, Wohl M, Mouren MC, Verpillat P, Ades J, Gorwood P. Meta-analysis of family-based association studies between the dopamine transporter gene and attention deficit hyperactivity disorder. *Psychiatr Genet* 2005; **15**(1)**:** 53-59.

170. Mill J, Xu X, Ronald A, Curran S, Price T, Knight J*, et al*. Quantitative trait locus analysis of candidate gene alleles associated with attention deficit hyperactivity disorder (ADHD) in five genes: DRD4, DAT1, DRD5, SNAP-25, and 5HT1B. *Am J Med Genet B Neuropsychiatr Genet* 2005; **133B**(1)**:** 68-73.

171. Yang B, Chan RC, Jing J, Li T, Sham P, Chen RY. A meta-analysis of association studies between the 10-repeat allele of a VNTR polymorphism in the 3'-UTR of dopamine transporter gene and attention deficit hyperactivity disorder. *Am J Med Genet B Neuropsychiatr Genet* 2007; **144B**(4)**:** 541-550.

172. Rao JS, Kellom M, Reese EA, Rapoport SI, Kim HW. Dysregulated glutamate and dopamine transporters in postmortem frontal cortex from bipolar and schizophrenic patients. *J Affect Disord* 2012; **136**(1-2)**:** 63-71.

173. Liu L, Yuan G, Cheng Z, Zhang G, Liu X, Zhang H. Identification of the mRNA expression status of the dopamine D2 receptor and dopamine transporter in peripheral blood lymphocytes of schizophrenia patients. *PLoS One* 2013; **8**(9)**:** e75259.

174. Markota M, Sin J, Pantazopoulos H, Jonilionis R, Berretta S. Reduced dopamine transporter expression in the amygdala of subjects diagnosed with schizophrenia. *Schizophr Bull* 2014; **40**(5)**:** 984-991.

175. Huang CC, Lu RB, Yen CH, Yeh YW, Chou HW, Kuo SC*, et al*. Dopamine transporter gene may be associated with bipolar disorder and its personality traits. *Eur Arch Psychiatry Clin Neurosci* 2015; **265**(4)**:** 281-290.

176. Nakamura K, Sekine Y, Ouchi Y, Tsujii M, Yoshikawa E, Futatsubashi M*, et al*. Brain serotonin and dopamine transporter bindings in adults with high-functioning autism. *Arch Gen Psychiatry* 2010; **67**(1)**:** 59-68.

177. Hamilton PJ, Campbell NG, Sharma S, Erreger K, Herborg Hansen F, Saunders C*, et al*. De novo mutation in the dopamine transporter gene associates dopamine dysfunction with autism spectrum disorder. *Mol Psychiatry* 2013; **18**(12)**:** 1315-1323.

178. Haeffel GJ, Getchell M, Koposov RA, Yrigollen CM, Deyoung CG, Klinteberg BA*, et al*. Association between polymorphisms in the dopamine transporter gene and depression: evidence for a gene-environment interaction in a sample of juvenile detainees. *Psychol Sci* 2008; **19**(1)**:** 62-69.

179. Amsterdam JD, Newberg AB, Soeller I, Shults J. Greater striatal dopamine transporter density may be associated with major depressive episode. *J Affect Disord* 2012; **141**(2-3)**:** 425-431.

180. Stolf AR, Szobot CM, Halpern R, Akutagava-Martins GC, Muller D, Guimaraes LS*, et al*. Crack cocaine users show differences in genotype frequencies of the 3' UTR variable number of tandem repeats of the dopamine transporter gene (DAT1/SLC6A3). *Neuropsychobiology* 2014; **70**(1)**:** 44-51.

181. Vasconcelos AC, Neto Ede S, Pinto GR, Yoshioka FK, Motta FJ, Vasconcelos DF*, et al*. Association study of the SLC6A3 VNTR (DAT) and DRD2/ANKK1 Taq1A polymorphisms with alcohol dependence in a population from northeastern Brazil. *Alcohol Clin Exp Res* 2015; **39**(2)**:** 205-211.

182. Curran S, Purcell S, Craig I, Asherson P, Sham P. The serotonin transporter gene as a QTL for ADHD. *Am J Med Genet B Neuropsychiatr Genet* 2005; **134B**(1)**:** 42-47.

183. Manor I, Eisenberg J, Tyano S, Sever Y, Cohen H, Ebstein RP*, et al*. Family-based association study of the serotonin transporter promoter region polymorphism (5-HTTLPR) in attention deficit hyperactivity disorder. *Am J Med Genet* 2001; **105**(1)**:** 91-95.

184. Fan JB, Sklar P. Meta-analysis reveals association between serotonin transporter gene STin2 VNTR polymorphism and schizophrenia. *Mol Psychiatry* 2005; **10**(10)**:** 928-938, 891.

185. Li W, Yang Y, Lin J, Wang S, Zhao J, Yang G*, et al*. Association of serotonin transporter gene (SLC6A4) polymorphisms with schizophrenia susceptibility and symptoms in a Chinese-Han population. *Prog Neuropsychopharmacol Biol Psychiatry* 2013; **44:** 290-295.

186. Jiang HY, Qiao F, Xu XF, Yang Y, Bai Y, Jiang LL. Meta-analysis confirms a functional polymorphism (5-HTTLPR) in the serotonin transporter gene conferring risk of bipolar disorder in European populations. *Neurosci Lett* 2013; **549:** 191-196.

187. Benedetti F, Bollettini I, Poletti S, Locatelli C, Lorenzi C, Pirovano A*, et al*. White matter microstructure in bipolar disorder is influenced by the serotonin transporter gene polymorphism 5-HTTLPR. *Genes Brain Behav* 2015; **14**(3)**:** 238-250.

188. Wassink TH, Hazlett HC, Epping EA, Arndt S, Dager SR, Schellenberg GD*, et al*. Cerebral cortical gray matter overgrowth and functional variation of the serotonin transporter gene in autism. *Arch Gen Psychiatry* 2007; **64**(6)**:** 709-717.

189. Wiggins JL, Peltier SJ, Bedoyan JK, Carrasco M, Welsh RC, Martin DM*, et al*. The impact of serotonin transporter genotype on default network connectivity in children and adolescents with autism spectrum disorders. *Neuroimage Clin* 2012; **2:** 17-24.

190. Haenisch B, Herms S, Mattheisen M, Steffens M, Breuer R, Strohmaier J*, et al*. Genome-wide association data provide further support for an association between 5-HTTLPR and major depressive disorder. *J Affect Disord* 2013; **146**(3)**:** 438-440.

191. Little K, Olsson CA, Whittle S, Youssef GJ, Byrne ML, Simmons JG*, et al*. Association between serotonin transporter genotype, brain structure and adolescent-onset major depressive disorder: a longitudinal prospective study. *Transl Psychiatry* 2014; **4:** e445.

192. Konishi T, Luo HR, Calvillo M, Mayo MS, Lin KM, Wan YJ. ADH1B*1, ADH1C*2, DRD2 (-141C Ins), and 5-HTTLPR are associated with alcoholism in Mexican American men living in Los Angeles. *Alcohol Clin Exp Res* 2004; **28**(8)**:** 1145-1152.

193. Cao J, Hudziak JJ, Li D. Multi-cultural association of the serotonin transporter gene (SLC6A4) with substance use disorder. *Neuropsychopharmacology* 2013; **38**(9)**:** 1737-1747.

194. Li J, Wang Y, Zhou R, Zhang H, Yang L, Wang B*, et al*. Association between polymorphisms in serotonin transporter gene and attention deficit hyperactivity disorder in Chinese Han subjects. *Am J Med Genet B Neuropsychiatr Genet* 2007; **144B**(1)**:** 14-19.

195. Banerjee E, Sinha S, Chatterjee A, Gangopadhyay PK, Singh M, Nandagopal K. A family-based study of Indian subjects from Kolkata reveals allelic association of the serotonin transporter intron-2 (STin2) polymorphism and attention-deficit-hyperactivity disorder (ADHD). *Am J Med Genet B Neuropsychiatr Genet* 2006; **141B**(4)**:** 361-366.

196. de Silva MG, Elliott K, Dahl HH, Fitzpatrick E, Wilcox S, Delatycki M*, et al*. Disruption of a novel member of a sodium/hydrogen exchanger family and DOCK3 is associated with an attention deficit hyperactivity disorder-like phenotype. *J Med Genet* 2003; **40**(10)**:** 733-740.

197. Kondapalli KC, Hack A, Schushan M, Landau M, Ben-Tal N, Rao R. Functional evaluation of autism-associated mutations in NHE9. *Nat Commun* 2013; **4:** 2510.

198. Schwede M, Garbett K, Mirnics K, Geschwind DH, Morrow EM. Genes for endosomal NHE6 and NHE9 are misregulated in autism brains. *Mol Psychiatry* 2014; **19**(3)**:** 277-279.

199. Barr CL, Feng Y, Wigg K, Bloom S, Roberts W, Malone M*, et al*. Identification of DNA variants in the SNAP-25 gene and linkage study of these polymorphisms and attention-deficit hyperactivity disorder. *Mol Psychiatry* 2000; **5**(4)**:** 405-409.

200. Brophy K, Hawi Z, Kirley A, Fitzgerald M, Gill M. Synaptosomal-associated protein 25 (SNAP-25) and attention deficit hyperactivity disorder (ADHD): evidence of linkage and association in the Irish population. *Mol Psychiatry* 2002; **7**(8)**:** 913-917.

201. Kustanovich V, Merriman B, McGough J, McCracken JT, Smalley SL, Nelson SF. Biased paternal transmission of SNAP-25 risk alleles in attention-deficit hyperactivity disorder. *Mol Psychiatry* 2003; **8**(3)**:** 309-315.

202. Fatemi SH, Earle JA, Stary JM, Lee S, Sedgewick J. Altered levels of the synaptosomal associated protein SNAP-25 in hippocampus of subjects with mood disorders and schizophrenia. *Neuroreport* 2001; **12**(15)**:** 3257-3262.

203. Wang Q, Wang Y, Ji W, Zhou G, He K, Li Z*, et al*. SNAP25 is associated with schizophrenia and major depressive disorder in the Han Chinese population. *J Clin Psychiatry* 2015; **76**(1)**:** e76-82.

204. Etain B, Dumaine A, Mathieu F, Chevalier F, Henry C, Kahn JP*, et al*. A SNAP25 promoter variant is associated with early-onset bipolar disorder and a high expression level in brain. *Mol Psychiatry* 2010; **15**(7)**:** 748-755.

205. Braida D, Guerini FR, Ponzoni L, Corradini I, De Astis S, Pattini L*, et al*. Association between SNAP-25 gene polymorphisms and cognition in autism: functional consequences and potential therapeutic strategies. *Transl Psychiatry* 2015; **5:** e500.

206. Lull ME, Erwin MS, Morgan D, Roberts DC, Vrana KE, Freeman WM. Persistent proteomic alterations in the medial prefrontal cortex with abstinence from cocaine self-administration. *Proteomics Clin Appl* 2009; **3**(4)**:** 462-472.

207. Feng Y, Crosbie J, Wigg K, Pathare T, Ickowicz A, Schachar R*, et al*. The SNAP25 gene as a susceptibility gene contributing to attention-deficit hyperactivity disorder. *Mol Psychiatry* 2005; **10**(11)**:** 998-1005, 1973.

208. Choi TK, Lee HS, Kim JW, Park TW, Song DH, Yook KW*, et al*. Support for the MnlI polymorphism of SNAP25; a Korean ADHD case-control study. *Mol Psychiatry* 2007; **12**(3)**:** 224-226.

209. Sheehan K, Lowe N, Kirley A, Mullins C, Fitzgerald M, Gill M*, et al*. Tryptophan hydroxylase 2 (TPH2) gene variants associated with ADHD. *Mol Psychiatry* 2005; **10**(10)**:** 944-949.

210. De Luca V, Likhodi O, Van Tol HH, Kennedy JL, Wong AH. Tryptophan hydroxylase 2 gene expression and promoter polymorphisms in bipolar disorder and schizophrenia. *Psychopharmacology (Berl)* 2005; **183**(3)**:** 378-382.

211. Xu XM, Ding M, Pang H, Wang BJ. TPH2 gene polymorphisms in the regulatory region are associated with paranoid schizophrenia in Northern Han Chinese. *Genet Mol Res* 2014; **13**(1)**:** 1497-1507.

212. Roche S, McKeon P. Support for tryptophan hydroxylase-2 as a susceptibility gene for bipolar affective disorder. *Psychiatr Genet* 2009; **19**(3)**:** 142-146.

213. Coon H, Dunn D, Lainhart J, Miller J, Hamil C, Battaglia A*, et al*. Possible association between autism and variants in the brain-expressed tryptophan hydroxylase gene (TPH2). *Am J Med Genet B Neuropsychiatr Genet* 2005; **135B**(1)**:** 42-46.

214. Yang SY, Yoo HJ, Cho IH, Park M, Kim SA. Association with tryptophan hydroxylase 2 gene polymorphisms and autism spectrum disorders in Korean families. *Neurosci Res* 2012; **73**(4)**:** 333-336.

215. Van Den Bogaert A, Sleegers K, De Zutter S, Heyrman L, Norrback KF, Adolfsson R*, et al*. Association of brain-specific tryptophan hydroxylase, TPH2, with unipolar and bipolar disorder in a Northern Swedish, isolated population. *Arch Gen Psychiatry* 2006; **63**(10)**:** 1103-1110.

216. Gao J, Pan Z, Jiao Z, Li F, Zhao G, Wei Q*, et al*. TPH2 gene polymorphisms and major depression--a meta-analysis. *PLoS One* 2012; **7**(5)**:** e36721.

217. Reuter M, Hennig J, Amelang M, Montag C, Korkut T, Hueweler A*, et al*. The role of the TPH1 and TPH2 genes for nicotine dependence: a genetic association study in two different age cohorts. *Neuropsychobiology* 2007; **56**(1)**:** 47-54.

218. Nielsen DA, Barral S, Proudnikov D, Kellogg S, Ho A, Ott J*, et al*. TPH2 and TPH1: association of variants and interactions with heroin addiction. *Behav Genet* 2008; **38**(2)**:** 133-150.

219. Kenar AN, Ay OI, Herken H, Erdal ME. Association of VAMP-2 and Syntaxin 1A Genes with Adult Attention Deficit Hyperactivity Disorder. *Psychiatry Investig* 2014; **11**(1)**:** 76-83.

220. Gao Q, Liu L, Chen Y, Li H, Yang L, Wang Y*, et al*. Synaptosome-related (SNARE) genes and their interactions contribute to the susceptibility and working memory of attention-deficit/hyperactivity disorder in males. *Prog Neuropsychopharmacol Biol Psychiatry* 2015; **57:** 132-139.

221. Yamada M, Takahashi K, Tsunoda M, Nishioka G, Kudo K, Ohata H*, et al*. Differential expression of VAMP2/synaptobrevin-2 after antidepressant and electroconvulsive treatment in rat frontal cortex. *Pharmacogenomics J* 2002; **2**(6)**:** 377-382.

222. Malki K, Keers R, Tosto MG, Lourdusamy A, Carboni L, Domenici E*, et al*. The endogenous and reactive depression subtypes revisited: integrative animal and human studies implicate multiple distinct molecular mechanisms underlying major depressive disorder. *BMC Med* 2014; **12:** 73.

223. Varodayan FP, Harrison NL. HSF1 transcriptional activity mediates alcohol induction of Vamp2 expression and GABA release. *Front Integr Neurosci* 2013; **7:** 89.

224. Lesch KP, Timmesfeld N, Renner TJ, Halperin R, Roser C, Nguyen TT*, et al*. Molecular genetics of adult ADHD: converging evidence from genome-wide association and extended pedigree linkage studies. *J Neural Transm (Vienna)* 2008; **115**(11)**:** 1573-1585.

225. Holmans P, Weissman MM, Zubenko GS, Scheftner WA, Crowe RR, Depaulo JR, Jr.*, et al*. Genetics of recurrent early-onset major depression (GenRED): final genome scan report. *Am J Psychiatry* 2007; **164**(2)**:** 248-258.

226. Lasky-Su J, Neale BM, Franke B, Anney RJ, Zhou K, Maller JB*, et al*. Genome-wide association scan of quantitative traits for attention deficit hyperactivity disorder identifies novel associations and confirms candidate gene associations. *Am J Med Genet B Neuropsychiatr Genet* 2008; **147B**(8)**:** 1345-1354.

227. Zhou K, Dempfle A, Arcos-Burgos M, Bakker SC, Banaschewski T, Biederman J*, et al*. Meta-analysis of genome-wide linkage scans of attention deficit hyperactivity disorder. *Am J Med Genet B Neuropsychiatr Genet* 2008; **147B**(8)**:** 1392-1398.

228. Eicher JD, Gruen JR. Language impairment and dyslexia genes influence language skills in children with autism spectrum disorders. *Autism Res* 2015; **8**(2)**:** 229-234.

229. Vrijenhoek T, Buizer-Voskamp JE, van der Stelt I, Strengman E, Sabatti C, Geurts van Kessel A*, et al*. Recurrent CNVs disrupt three candidate genes in schizophrenia patients. *Am J Hum Genet* 2008; **83**(4)**:** 504-510.

230. Sullivan PF, Lin D, Tzeng JY, van den Oord E, Perkins D, Stroup TS*, et al*. Genomewide association for schizophrenia in the CATIE study: results of stage 1. *Mol Psychiatry* 2008; **13**(6)**:** 570-584.

231. Wang KS, Liu XF, Aragam N. A genome-wide meta-analysis identifies novel loci associated with schizophrenia and bipolar disorder. *Schizophr Res* 2010; **124**(1-3)**:** 192-199.

232. Marshall CR, Noor A, Vincent JB, Lionel AC, Feuk L, Skaug J*, et al*. Structural variation of chromosomes in autism spectrum disorder. *Am J Hum Genet* 2008; **82**(2)**:** 477-488.

233. Glessner JT, Wang K, Cai G, Korvatska O, Kim CE, Wood S*, et al*. Autism genome-wide copy number variation reveals ubiquitin and neuronal genes. *Nature* 2009; **459**(7246)**:** 569-573.

234. Lionel AC, Tammimies K, Vaags AK, Rosenfeld JA, Ahn JW, Merico D*, et al*. Disruption of the ASTN2/TRIM32 locus at 9q33.1 is a risk factor in males for autism spectrum disorders, ADHD and other neurodevelopmental phenotypes. *Hum Mol Genet* **23**(10)**:** 2752-2768.

235. Uhl GR, Drgon T, Liu QR, Johnson C, Walther D, Komiyama T*, et al*. Genome-wide association for methamphetamine dependence: convergent results from 2 samples. *Arch Gen Psychiatry* 2008; **65**(3)**:** 345-355.

236. Stergiakouli E, Hamshere M, Holmans P, Langley K, Zaharieva I, Hawi Z*, et al*. Investigating the contribution of common genetic variants to the risk and pathogenesis of ADHD. *Am J Psychiatry* 2012; **169**(2)**:** 186-194.

237. Zhang F, Wang G, Shugart YY, Xu Y, Liu C, Wang L*, et al*. Association analysis of a functional variant in ATXN2 with schizophrenia. *Neurosci Lett* 2014; **562:** 24-27.

238. Ebejer JL, Duffy DL, van der Werf J, Wright MJ, Montgomery G, Gillespie NA*, et al*. Genome-wide association study of inattention and hyperactivity-impulsivity measured as quantitative traits. *Twin Res Hum Genet* 2013; **16**(2)**:** 560-574.

239. Hinney A, Scherag A, Jarick I, Albayrak O, Putter C, Pechlivanis S*, et al*. Genome-wide association study in German patients with attention deficit/hyperactivity disorder. *Am J Med Genet B Neuropsychiatr Genet* 2011; **156B**(8)**:** 888-897.

240. Alisch RS, Chopra P, Fox AS, Chen K, White AT, Roseboom PH*, et al*. Differentially methylated plasticity genes in the amygdala of young primates are linked to anxious temperament, an at risk phenotype for anxiety and depressive disorders. *J Neurosci* 2014; **34**(47)**:** 15548-15556.

241. Neale BM, Lasky-Su J, Anney R, Franke B, Zhou K, Maller JB*, et al*. Genome-wide association scan of attention deficit hyperactivity disorder. *Am J Med Genet B Neuropsychiatr Genet* 2008; **147B**(8)**:** 1337-1344.

242. Xu W, Cohen-Woods S, Chen Q, Noor A, Knight J, Hosang G*, et al*. Genome-wide association study of bipolar disorder in Canadian and UK populations corroborates disease loci including SYNE1 and CSMD1. *BMC Med Genet* 2014; **15:** 2.

243. Christian SL, Brune CW, Sudi J, Kumar RA, Liu S, Karamohamed S*, et al*. Novel submicroscopic chromosomal abnormalities detected in autism spectrum disorder. *Biol Psychiatry* 2008; **63**(12)**:** 1111-1117.

244. Edwards AC, Aliev F, Bierut LJ, Bucholz KK, Edenberg H, Hesselbrock V*, et al*. Genome-wide association study of comorbid depressive syndrome and alcohol dependence. *Psychiatr Genet* 2012; **22**(1)**:** 31-41.

245. Yang J, Wang S, Yang Z, Hodgkinson CA, Iarikova P, Ma JZ*, et al*. The contribution of rare and common variants in 30 genes to risk nicotine dependence. *Mol Psychiatry* 2015; **20**(11)**:** 1467-1478.

246. Neale BM, Medland S, Ripke S, Anney RJ, Asherson P, Buitelaar J*, et al*. Case-control genome-wide association study of attention-deficit/hyperactivity disorder. *J Am Acad Child Adolesc Psychiatry* 2010; **49**(9)**:** 906-920.

247. Blouin JL, Dombroski BA, Nath SK, Lasseter VK, Wolyniec PS, Nestadt G*, et al*. Schizophrenia susceptibility loci on chromosomes 13q32 and 8p21. *Nat Genet* 1998; **20**(1)**:** 70-73.

248. Detera-Wadleigh SD, Badner JA, Berrettini WH, Yoshikawa T, Goldin LR, Turner G*, et al*. A high-density genome scan detects evidence for a bipolar-disorder susceptibility locus on 13q32 and other potential loci on 1q32 and 18p11.2. *Proc Natl Acad Sci U S A* 1999; **96**(10)**:** 5604-5609.

249. Chavarria-Siles I, Contreras-Rojas J, Hare E, Walss-Bass C, Quezada P, Dassori A*, et al*. Cannabinoid receptor 1 gene (CNR1) and susceptibility to a quantitative phenotype for hebephrenic schizophrenia. *Am J Med Genet B Neuropsychiatr Genet* 2008; **147**(3)**:** 279-284.

250. Ho BC, Wassink TH, Ziebell S, Andreasen NC. Cannabinoid receptor 1 gene polymorphisms and marijuana misuse interactions on white matter and cognitive deficits in schizophrenia. *Schizophr Res* 2011; **128**(1-3)**:** 66-75.

251. Monteleone P, Bifulco M, Maina G, Tortorella A, Gazzerro P, Proto MC*, et al*. Investigation of CNR1 and FAAH endocannabinoid gene polymorphisms in bipolar disorder and major depression. *Pharmacol Res* 2010; **61**(5)**:** 400-404.

252. Mitjans M, Serretti A, Fabbri C, Gasto C, Catalan R, Fananas L*, et al*. Screening genetic variability at the CNR1 gene in both major depression etiology and clinical response to citalopram treatment. *Psychopharmacology (Berl)* 2013; **227**(3)**:** 509-519.

253. Ponce G, Hoenicka J, Rubio G, Ampuero I, Jimenez-Arriero MA, Rodriguez-Jimenez R*, et al*. Association between cannabinoid receptor gene (CNR1) and childhood attention deficit/hyperactivity disorder in Spanish male alcoholic patients. *Mol Psychiatry* 2003; **8**(5)**:** 466-467.

254. Zuo L, Kranzler HR, Luo X, Covault J, Gelernter J. CNR1 variation modulates risk for drug and alcohol dependence. *Biol Psychiatry* 2007; **62**(6)**:** 616-626.

255. Rotter A, Bayerlein K, Hansbauer M, Weiland J, Sperling W, Kornhuber J*, et al*. CB1 and CB2 receptor expression and promoter methylation in patients with cannabis dependence. *Eur Addict Res* 2013; **19**(1)**:** 13-20.

256. Djurovic S, Gustafsson O, Mattingsdal M, Athanasiu L, Bjella T, Tesli M*, et al*. A genome-wide association study of bipolar disorder in Norwegian individuals, followed by replication in Icelandic sample. *J Affect Disord* 2010; **126**(1-2)**:** 312-316.

257. Pagnamenta AT, Bacchelli E, de Jonge MV, Mirza G, Scerri TS, Minopoli F*, et al*. Characterization of a family with rare deletions in CNTNAP5 and DOCK4 suggests novel risk loci for autism and dyslexia. *Biol Psychiatry* 2010; **68**(4)**:** 320-328.

258. Ramos-Miguel A, Beasley CL, Dwork AJ, Mann JJ, Rosoklija G, Barr AM*, et al*. Increased SNARE Protein-Protein Interactions in Orbitofrontal and Anterior Cingulate Cortices in Schizophrenia. *Biol Psychiatry* 2014; **78**(6)**:** 361-373.

259. Zakharyan R, Atshemyan S, Boyajyan A. Risk and protective effects of the complexin-2 gene and gene-environment interactions in schizophrenia. *Recent Adv DNA Gene Seq* 2014; **8**(1)**:** 30-34.

260. Lewis CM, Levinson DF, Wise LH, DeLisi LE, Straub RE, Hovatta I*, et al*. Genome scan meta-analysis of schizophrenia and bipolar disorder, part II: Schizophrenia. *Am J Hum Genet* 2003; **73**(1)**:** 34-48.

261. Consortium WTCC. Genome-wide association study of 14,000 cases of seven common diseases and 3,000 shared controls. *Nature* 2007; **447**(7145)**:** 661-678.

262. Havik B, Le Hellard S, Rietschel M, Lybaek H, Djurovic S, Mattheisen M*, et al*. The complement control-related genes CSMD1 and CSMD2 associate to schizophrenia. *Biol Psychiatry* 2011; **70**(1)**:** 35-42.

263. Johnson C, Drgon T, Liu QR, Walther D, Edenberg H, Rice J*, et al*. Pooled association genome scanning for alcohol dependence using 104,268 SNPs: validation and use to identify alcoholism vulnerability loci in unrelated individuals from the collaborative study on the genetics of alcoholism. *Am J Med Genet B Neuropsychiatr Genet* 2006; **141B**(8)**:** 844-853.

264. Havik B, Degenhardt FA, Johansson S, Fernandes CP, Hinney A, Scherag A*, et al*. DCLK1 variants are associated across schizophrenia and attention deficit/hyperactivity disorder. *PLoS One* 2012; **7**(4)**:** e35424.

265. Allen-Brady K, Miller J, Matsunami N, Stevens J, Block H, Farley M*, et al*. A high-density SNP genome-wide linkage scan in a large autism extended pedigree. *Mol Psychiatry* 2009; **14**(6)**:** 590-600.

266. Szatmari P, Paterson AD, Zwaigenbaum L, Roberts W, Brian J, Liu XQ*, et al*. Mapping autism risk loci using genetic linkage and chromosomal rearrangements. *Nat Genet* 2007; **39**(3)**:** 319-328.

267. Li MD, Ma JZ, Payne TJ, Lou XY, Zhang D, Dupont RT*, et al*. Genome-wide linkage scan for nicotine dependence in European Americans and its converging results with African Americans in the Mid-South Tobacco Family sample. *Mol Psychiatry* 2008; **13**(4)**:** 407-416.

268. Zhang C, Fang Y, Xie B, Cheng W, Du Y, Wang D*, et al*. DNA methyltransferase 3B gene increases risk of early onset schizophrenia. *Neurosci Lett* 2009; **462**(3)**:** 308-311.

269. Saradalekshmi KR, Neetha NV, Sathyan S, Nair IV, Nair CM, Banerjee M. DNA methyl transferase (DNMT) gene polymorphisms could be a primary event in epigenetic susceptibility to schizophrenia. *PLoS One* 2014; **9**(5)**:** e98182.

270. Higuchi F, Uchida S, Yamagata H, Otsuki K, Hobara T, Abe N*, et al*. State-dependent changes in the expression of DNA methyltransferases in mood disorder patients. *J Psychiatr Res* 2011; **45**(10)**:** 1295-1300.

271. Egger G, Roetzer KM, Noor A, Lionel AC, Mahmood H, Schwarzbraun T*, et al*. Identification of risk genes for autism spectrum disorder through copy number variation analysis in Austrian families. *Neurogenetics* 2014; **15**(2)**:** 117-127.

272. Girirajan S, Dennis MY, Baker C, Malig M, Coe BP, Campbell CD*, et al*. Refinement and discovery of new hotspots of copy-number variation associated with autism spectrum disorder. *Am J Hum Genet* 2013; **92**(2)**:** 221-237.

273. Arinami T, Ohtsuki T, Ishiguro H, Ujike H, Tanaka Y, Morita Y*, et al*. Genomewide high-density SNP linkage analysis of 236 Japanese families supports the existence of schizophrenia susceptibility loci on chromosomes 1p, 14q, and 20p. *Am J Hum Genet* 2005; **77**(6)**:** 937-944.

274. Sklar P, Smoller JW, Fan J, Ferreira MA, Perlis RH, Chambert K*, et al*. Whole-genome association study of bipolar disorder. *Mol Psychiatry* 2008; **13**(6)**:** 558-569.

275. Nurnberger JI, Jr., Koller DL, Jung J, Edenberg HJ, Foroud T, Guella I*, et al*. Identification of pathways for bipolar disorder: a meta-analysis. *JAMA Psychiatry* 2014; **71**(6)**:** 657-664.

276. Chien WH, Gau SS, Chen CH, Tsai WC, Wu YY, Chen PH*, et al*. Increased gene expression of FOXP1 in patients with autism spectrum disorders. *Mol Autism* 2013; **4**(1)**:** 23.

277. Wang KS, Zhang Q, Liu X, Wu L, Zeng M. PKNOX2 is associated with formal thought disorder in schizophrenia: a meta-analysis of two genome-wide association studies. *J Mol Neurosci* 2012; **48**(1)**:** 265-272.

278. Castellani CA, Awamleh Z, Melka MG, O'Reilly RL, Singh SM. Copy number variation distribution in six monozygotic twin pairs discordant for schizophrenia. *Twin Res Hum Genet* 2014; **17**(2)**:** 108-120.

279. Hirata Y, Zai CC, Souza RP, Lieberman JA, Meltzer HY, Kennedy JL. Association study of GRIK1 gene polymorphisms in schizophrenia: case-control and family-based studies. *Hum Psychopharmacol* 2012; **27**(4)**:** 345-351.

280. Kranzler HR, Gelernter J, Anton RF, Arias AJ, Herman A, Zhao H*, et al*. Association of markers in the 3' region of the GluR5 kainate receptor subunit gene to alcohol dependence. *Alcohol Clin Exp Res* 2009; **33**(5)**:** 925-930.

281. Yang L, Neale BM, Liu L, Lee SH, Wray NR, Ji N*, et al*. Polygenic transmission and complex neuro developmental network for attention deficit hyperactivity disorder: genome-wide association study of both common and rare variants. *Am J Med Genet B Neuropsychiatr Genet* 2013; **162B**(5)**:** 419-430.

282. Pickard BS, Malloy MP, Christoforou A, Thomson PA, Evans KL, Morris SW*, et al*. Cytogenetic and genetic evidence supports a role for the kainate-type glutamate receptor gene, GRIK4, in schizophrenia and bipolar disorder. *Mol Psychiatry* 2006; **11**(9)**:** 847-857.

283. Pickard BS, Knight HM, Hamilton RS, Soares DC, Walker R, Boyd JK*, et al*. A common variant in the 3'UTR of the GRIK4 glutamate receptor gene affects transcript abundance and protects against bipolar disorder. *Proc Natl Acad Sci U S A* 2008; **105**(39)**:** 14940-14945.

284. Griswold AJ, Ma D, Cukier HN, Nations LD, Schmidt MA, Chung RH*, et al*. Evaluation of copy number variations reveals novel candidate genes in autism spectrum disorder-associated pathways. *Hum Mol Genet* 2012; **21**(15)**:** 3513-3523.

285. Ohnuma T, Augood SJ, Arai H, McKenna PJ, Emson PC. Expression of the human excitatory amino acid transporter 2 and metabotropic glutamate receptors 3 and 5 in the prefrontal cortex from normal individuals and patients with schizophrenia. *Brain Res Mol Brain Res* 1998; **56**(1-2)**:** 207-217.

286. Devon RS, Anderson S, Teague PW, Muir WJ, Murray V, Pelosi AJ*, et al*. The genomic organisation of the metabotropic glutamate receptor subtype 5 gene, and its association with schizophrenia. *Mol Psychiatry* 2001; **6**(3)**:** 311-314.

287. Zantomio D, Chana G, Laskaris L, Testa R, Everall I, Pantelis C*, et al*. Convergent evidence for mGluR5 in synaptic and neuroinflammatory pathways implicated in ASD. *Neurosci Biobehav Rev* 2015; **52:** 172-177.

288. Deschwanden A, Karolewicz B, Feyissa AM, Treyer V, Ametamey SM, Johayem A*, et al*. Reduced metabotropic glutamate receptor 5 density in major depression determined by [(11)C]ABP688 PET and postmortem study. *Am J Psychiatry* 2011; **168**(7)**:** 727-734.

289. Chandley MJ, Szebeni A, Szebeni K, Crawford JD, Stockmeier CA, Turecki G*, et al*. Elevated gene expression of glutamate receptors in noradrenergic neurons from the locus coeruleus in major depression. *Int J Neuropsychopharmacol* 2014; **17**(10)**:** 1569-1578.

290. Hulka LM, Treyer V, Scheidegger M, Preller KH, Vonmoos M, Baumgartner MR*, et al*. Smoking but not cocaine use is associated with lower cerebral metabotropic glutamate receptor 5 density in humans. *Mol Psychiatry* 2014; **19**(5)**:** 625-632.

291. Milella MS, Marengo L, Larcher K, Fotros A, Dagher A, Rosa-Neto P*, et al*. Limbic system mGluR5 availability in cocaine dependent subjects: a high-resolution PET [(11)C]ABP688 study. *Neuroimage* 2014; **98:** 195-202.

292. Yanagi M, Joho RH, Southcott SA, Shukla AA, Ghose S, Tamminga CA. Kv3.1-containing K(+) channels are reduced in untreated schizophrenia and normalized with antipsychotic drugs. *Mol Psychiatry* 2014; **19**(5)**:** 573-579.

293. Trikalinos TA, Karvouni A, Zintzaras E, Ylisaukko-oja T, Peltonen L, Jarvela I*, et al*. A heterogeneity-based genome search meta-analysis for autism-spectrum disorders. *Mol Psychiatry* 2006; **11**(1)**:** 29-36.

294. Duvall JA, Lu A, Cantor RM, Todd RD, Constantino JN, Geschwind DH. A quantitative trait locus analysis of social responsiveness in multiplex autism families. *Am J Psychiatry* 2007; **164**(4)**:** 656-662.

295. Gelernter J, Kranzler HR, Sherva R, Koesterer R, Almasy L, Zhao H*, et al*. Genome-wide association study of opioid dependence: multiple associations mapped to calcium and potassium pathways. *Biol Psychiatry* 2014; **76**(1)**:** 66-74.

296. Sebat J, Lakshmi B, Malhotra D, Troge J, Lese-Martin C, Walsh T*, et al*. Strong association of de novo copy number mutations with autism. *Science* 2007; **316**(5823)**:** 445-449.

297. Le-Niculescu H, Balaraman Y, Patel S, Tan J, Sidhu K, Jerome RE*, et al*. Towards understanding the schizophrenia code: an expanded convergent functional genomics approach. *Am J Med Genet B Neuropsychiatr Genet* 2007; **144B**(2)**:** 129-158.

298. Xie C, Wang ZC, Liu XF, Wang L, Yang MS. Association between schizophrenia and single nucleotide polymorphisms in lipoprotein lipase gene in a Han Chinese population. *Psychiatr Genet* 2011; **21**(6)**:** 307-314.

299. Koide T, Banno M, Aleksic B, Yamashita S, Kikuchi T, Kohmura K*, et al*. Common variants in MAGI2 gene are associated with increased risk for cognitive impairment in schizophrenic patients. *PLoS One* 2012; **7**(5)**:** e36836.

300. Bouras C, Kovari E, Hof PR, Riederer BM, Giannakopoulos P. Anterior cingulate cortex pathology in schizophrenia and bipolar disorder. *Acta Neuropathol* 2001; **102**(4)**:** 373-379.

301. Mitkus SN, Hyde TM, Vakkalanka R, Kolachana B, Weinberger DR, Kleinman JE*, et al*. Expression of oligodendrocyte-associated genes in dorsolateral prefrontal cortex of patients with schizophrenia. *Schizophr Res* 2008; **98**(1-3)**:** 129-138.

302. Aston C, Jiang L, Sokolov BP. Transcriptional profiling reveals evidence for signaling and oligodendroglial abnormalities in the temporal cortex from patients with major depressive disorder. *Mol Psychiatry* 2005; **10**(3)**:** 309-322.

303. Albertson DN, Pruetz B, Schmidt CJ, Kuhn DM, Kapatos G, Bannon MJ. Gene expression profile of the nucleus accumbens of human cocaine abusers: evidence for dysregulation of myelin. *J Neurochem* 2004; **88**(5)**:** 1211-1219.

304. Lee Y, Mattai A, Long R, Rapoport JL, Gogtay N, Addington AM. Microduplications disrupting the MYT1L gene (2p25.3) are associated with schizophrenia. *Psychiatr Genet* 2012; **22**(4)**:** 206-209.

305. Meyer KJ, Axelsen MS, Sheffield VC, Patil SR, Wassink TH. Germline mosaic transmission of a novel duplication of PXDN and MYT1L to two male half-siblings with autism. *Psychiatr Genet* 2012; **22**(3)**:** 137-140.

306. Wang T, Zeng Z, Li T, Liu J, Li J, Li Y*, et al*. Common SNPs in myelin transcription factor 1-like (MYT1L): association with major depressive disorder in the Chinese Han population. *PLoS One* 2010; **5**(10)**:** e13662.

307. Smith EN, Bloss CS, Badner JA, Barrett T, Belmonte PL, Berrettini W*, et al*. Genome-wide association study of bipolar disorder in European American and African American individuals. *Mol Psychiatry* 2009; **14**(8)**:** 755-763.

308. Luciano M, Huffman JE, Arias-Vasquez A, Vinkhuyzen AA, Middeldorp CM, Giegling I*, et al*. Genome-wide association uncovers shared genetic effects among personality traits and mood states. *Am J Med Genet B Neuropsychiatr Genet* 2012; **159B**(6)**:** 684-695.

309. Xing G, Zhang L, Russell S, Post R. Reduction of dopamine-related transcription factors Nurr1 and NGFI-B in the prefrontal cortex in schizophrenia and bipolar disorders. *Schizophr Res* 2006; **84**(1)**:** 36-56.

310. Guillozet-Bongaarts AL, Hyde TM, Dalley RA, Hawrylycz MJ, Henry A, Hof PR*, et al*. Altered gene expression in the dorsolateral prefrontal cortex of individuals with schizophrenia. *Mol Psychiatry* 2014; **19**(4)**:** 478-485.

311. Chuang HC, Huang TN, Hsueh YP. T-Brain-1--A Potential Master Regulator in Autism Spectrum Disorders. *Autism Res* 2015; **8**(4)**:** 412-426.

312. Kerman IA, Bernard R, Bunney WE, Jones EG, Schatzberg AF, Myers RM*, et al*. Evidence for transcriptional factor dysregulation in the dorsal raphe nucleus of patients with major depressive disorder. *Front Neurosci* 2012; **6:** 135.

313. Bannon MJ, Pruetz B, Manning-Bog AB, Whitty CJ, Michelhaugh SK, Sacchetti P*, et al*. Decreased expression of the transcription factor NURR1 in dopamine neurons of cocaine abusers. *Proc Natl Acad Sci U S A* 2002; **99**(9)**:** 6382-6385.

314. Wei YM, Du YL, Nie YQ, Li YY, Wan YJ. Nur-related receptor 1 gene polymorphisms and alcohol dependence in Mexican Americans. *World J Gastroenterol* 2012; **18**(37)**:** 5276-5282.

315. Reif A, Herterich S, Strobel A, Ehlis AC, Saur D, Jacob CP*, et al*. A neuronal nitric oxide synthase (NOS-I) haplotype associated with schizophrenia modifies prefrontal cortex function. *Mol Psychiatry* 2006; **11**(3)**:** 286-300.

316. Candemir E, Kollert L, Weissflog L, Geis M, Muller A, Post AM*, et al*. Interaction of NOS1AP with the NOS-I PDZ domain: Implications for schizophrenia-related alterations in dendritic morphology. *Eur Neuropsychopharmacol* 2016.

317. Kittel-Schneider S, Reuss M, Meyer A, Weber H, Gessner A, Leistner C*, et al*. Multi-level biomarker analysis of nitric oxide synthase isoforms in bipolar disorder and adult ADHD. *J Psychopharmacol* 2014; **29**(1)**:** 31-38.

318. Kim HW, Cho SC, Kim JW, Cho IH, Kim SA, Park M*, et al*. Family-based association study between NOS-I and -IIA polymorphisms and autism spectrum disorders in Korean trios. *Am J Med Genet B Neuropsychiatr Genet* 2009; **150B**(2)**:** 300-306.

319. Abkevich V, Camp NJ, Hensel CH, Neff CD, Russell DL, Hughes DC*, et al*. Predisposition locus for major depression at chromosome 12q22-12q23.2. *Am J Hum Genet* 2003; **73**(6)**:** 1271-1281.

320. Galecki P, Maes M, Florkowski A, Lewinski A, Galecka E, Bienkiewicz M*, et al*. Association between inducible and neuronal nitric oxide synthase polymorphisms and recurrent depressive disorder. *J Affect Disord* 2011; **129**(1-3)**:** 175-182.

321. Ro M, Park J, Nam M, Bang HJ, Yang J, Choi KS*, et al*. Association between peroxisomal biogenesis factor 7 and autism spectrum disorders in a Korean population. *J Child Neurol* 2012; **27**(10)**:** 1270-1275.

322. Yu TW, Chahrour MH, Coulter ME, Jiralerspong S, Okamura-Ikeda K, Ataman B*, et al*. Using whole-exome sequencing to identify inherited causes of autism. *Neuron* 2013; **77**(2)**:** 259-273.

323. Kane JK, Konu O, Ma JZ, Li MD. Nicotine coregulates multiple pathways involved in protein modification/degradation in rat brain. *Brain Res Mol Brain Res* 2004; **132**(2)**:** 181-191.

324. Yang BZ, Han S, Kranzler HR, Farrer LA, Gelernter J. A genomewide linkage scan of cocaine dependence and major depressive episode in two populations. *Neuropsychopharmacology* 2011; **36**(12)**:** 2422-2430.

325. Maziade M, Roy MA, Chagnon YC, Cliche D, Fournier JP, Montgrain N*, et al*. Shared and specific susceptibility loci for schizophrenia and bipolar disorder: a dense genome scan in Eastern Quebec families. *Mol Psychiatry* 2005; **10**(5)**:** 486-499.

326. Yang Z, Ma X, Wang Y, Wang J, Xiang B, Wu J*, et al*. Association of APC and REEP5 gene polymorphisms with major depression disorder and treatment response to antidepressants in a Han Chinese population. *Gen Hosp Psychiatry* 2012; **34**(5)**:** 571-577.

327. Etain B, Jamain S, Milhiet V, Lajnef M, Boudebesse C, Dumaine A*, et al*. Association between circadian genes, bipolar disorders and chronotypes. *Chronobiol Int* 2014; **31**(7)**:** 807-814.

328. Lai YC, Kao CF, Lu ML, Chen HC, Chen PY, Chen CH*, et al*. Investigation of associations between NR1D1, RORA and RORB genes and bipolar disorder. *PLoS One* 2015; **10**(3)**:** e0121245.

329. Hu VW, Sarachana T, Kim KS, Nguyen A, Kulkarni S, Steinberg ME*, et al*. Gene expression profiling differentiates autism case-controls and phenotypic variants of autism spectrum disorders: evidence for circadian rhythm dysfunction in severe autism. *Autism Res* 2009; **2**(2)**:** 78-97.

330. Nguyen A, Rauch TA, Pfeifer GP, Hu VW. Global methylation profiling of lymphoblastoid cell lines reveals epigenetic contributions to autism spectrum disorders and a novel autism candidate gene, RORA, whose protein product is reduced in autistic brain. *Faseb J* 2010; **24**(8)**:** 3036-3051.

331. Terracciano A, Tanaka T, Sutin AR, Sanna S, Deiana B, Lai S*, et al*. Genome-wide association scan of trait depression. *Biol Psychiatry* 2010; **68**(9)**:** 811-817.

332. Ming Q, Wang X, Chai Q, Yi J, Yao S. Retinoid-related orphan receptor alpha (RORA) gene variation is associated with trait depression. *Psychiatry Res* 2015; **229**(1-2)**:** 629-630.

333. Verma R, Holmans P, Knowles JA, Grover D, Evgrafov OV, Crowe RR*, et al*. Linkage disequilibrium mapping of a chromosome 15q25-26 major depression linkage region and sequencing of NTRK3. *Biol Psychiatry* 2008; **63**(12)**:** 1185-1189.

334. Wang KS, Liu X, Zhang Q, Zeng M. ANAPC1 and SLCO3A1 are associated with nicotine dependence: meta-analysis of genome-wide association studies. *Drug Alcohol Depend* 2012; **124**(3)**:** 325-332.

335. El-Ansary A, Al-Ayadhi L. Neuroinflammation in autism spectrum disorders. *J Neuroinflammation* 2012; **9:** 265.

336. de Mooij-van Malsen JG, van Lith HA, Laarakker MC, Brandys MK, Oppelaar H, Collier DA*, et al*. Cross-species genetics converge to TLL2 for mouse avoidance behavior and human bipolar disorder. *Genes Brain Behav* 2013; **12**(6)**:** 653-657.

337. Schwab SG, Handoko HY, Kusumawardhani A, Widyawati I, Amir N, Nasrun MW*, et al*. Genome-wide scan in 124 Indonesian sib-pair families with schizophrenia reveals genome-wide significant linkage to a locus on chromosome 3p26-21. *Am J Med Genet B Neuropsychiatr Genet* 2008; **147B**(7)**:** 1245-1252.

338. Xu C, Aragam N, Li X, Villla EC, Wang L, Briones D*, et al*. BCL9 and C9orf5 are associated with negative symptoms in schizophrenia: meta-analysis of two genome-wide association studies. *PLoS One* 2013; **8**(1)**:** e51674.
