# Supplementary material for: Preliminary evidence for association of genetic variants in pri-miR-34b/c and abnormal miR-34c expression with attention deficit and hyperactivity disorder
Source: Transl Psychiatry. 2016 Aug 30;6(8):e879–. doi: 10.1038/tp.2016.151 (PMC5022091; doi:10.1038/tp.2016.151)
Supplement: Supplementary Table 7 [file tp2016151x8.doc]

**Supplementary Table 7** MiRNA binding site enrichment analyses.

| **hsa_ACTGCCT, MIR-34BC=215 O=13; E=5.49; R=2.37; rawP=3.8e-03; adjP=8.4e-03** | | | |
| --- | --- | --- | --- |
| **microRNA Target ID** | **Ensembl Gene ID** | **Entrez Gene ID** | **Description** |
| *SNTA1* | ENSG00000101400 | 6640 | syntrophin, alpha 1 |
| *XKR6* | ENSG00000171044 | 286046 | XK, Kell blood group complex subunit-related family, member 6 |
| *BRD4* | ENSG00000141867 | 23476 | bromodomain containing 4 |
| *HTR2C* | ENSG00000147246 | 3358 | 5-hydroxytryptamine (serotonin) receptor 2C, G protein-coupled |
| *MARCH5* | ENSG00000198060 | 54708 | membrane-associated ring finger (C3HC4) 5 |
| *ELMOD1* | ENSG00000110675 | 55531 | ELMO/CED-12 domain containing 1 |
| *VAMP2* | ENSG00000220205,ENSG00000263620 | 6844 | vesicle-associated membrane protein 2 (synaptobrevin 2) |
| *MORN4* | ENSG00000171160 | 118812 | MORN repeat containing 4 |
| *COL12A1* | ENSG00000111799 | 1303 | collagen, type XII, alpha 1 |
| *ABCC1* | ENSG00000103222 | 4363 | ATP-binding cassette, sub-family C (CFTR/MRP), member 1 |

**C:** number of reference genes in the category; **O:** number of genes in the gene set and also in the category; **E:** expected number in the category; **R:** Ratio of enrichment; **RawP:** P-value from hypergeometric test; **AdjP:** p value adjusted by the multiple test adjustment
