# Supplementary material for: Preliminary evidence for association of genetic variants in pri-miR-34b/c and abnormal miR-34c expression with attention deficit and hyperactivity disorder
Source: Transl Psychiatry. 2016 Aug 30;6(8):e879–. doi: 10.1038/tp.2016.151 (PMC5022091; doi:10.1038/tp.2016.151)
Supplement: Supplementary Table 8 [file tp2016151x9.doc]

**Supplementary Table 8** Results from the GO term enrichment analyses using the DAVID tool. A total of 36 categories were found nominally associated with genes in the dataset from the *trans*-eQTL analysis regarding the rs4938723T risk variant in the pri-miR-34b/c promoter (P-value<0.05).

| **Category** | **Term** | **Count** | **Fold Enrichment** | **P-Value** | **Benjamini** |
| --- | --- | --- | --- | --- | --- |
| GOTERM_BP_FAT | GO:0006690~icosanoid metabolic process | 7 | 4.81 | 0.0031 | 1.00 |
| GOTERM_BP_FAT | GO:0006508~proteolysis | 49 | 1.50 | 0.0042 | 0.99 |
| GOTERM_BP_FAT | GO:0006691~leukotriene metabolic process | 5 | 7.34 | 0.0042 | 0.95 |
| GOTERM_BP_FAT | GO:0033559~unsaturated fatty acid metabolic process | 7 | 4.43 | 0.0047 | 0.91 |
| GOTERM_BP_FAT | GO:0043449~cellular alkene metabolic process | 5 | 7.02 | 0.0050 | 0.88 |
| GOTERM_BP_FAT | GO:0007411~axon guidance | 10 | 3.02 | 0.0059 | 0.87 |
| GOTERM_BP_FAT | GO:0007409~axonogenesis | 14 | 2.34 | 0.0070 | 0.88 |
| GOTERM_BP_FAT | GO:0007586~digestion | 9 | 3.19 | 0.0071 | 0.84 |
| GOTERM_BP_FAT | GO:0048741~skeletal muscle fiber development | 5 | 5.77 | 0.0102 | 0.91 |
| GOTERM_BP_FAT | GO:0040007~growth | 13 | 2.29 | 0.0115 | 0.91 |
| GOTERM_BP_FAT | GO:0048667~cell morphogenesis involved in neuron differentiation | 14 | 2.16 | 0.0131 | 0.92 |
| GOTERM_BP_FAT | GO:0032990~cell part morphogenesis | 16 | 2.02 | 0.0135 | 0.91 |
| GOTERM_BP_FAT | GO:0050778~positive regulation of immune response | 11 | 2.45 | 0.0146 | 0.91 |
| GOTERM_BP_FAT | GO:0046456~icosanoid biosynthetic process | 5 | 5.21 | 0.0146 | 0.89 |
| GOTERM_BP_FAT | GO:0048584~positive regulation of response to stimulus | 15 | 2.05 | 0.0151 | 0.88 |
| GOTERM_BP_FAT | GO:0048812~neuron projection morphogenesis | 14 | 2.12 | 0.0151 | 0.87 |
| GOTERM_BP_FAT | GO:0030182~neuron differentiation | 23 | 1.70 | 0.0175 | 0.89 |
| GOTERM_BP_FAT | GO:0048589~developmental growth | 8 | 2.97 | 0.0180 | 0.88 |
| GOTERM_BP_FAT | GO:0000904~cell morphogenesis involved in differentiation | 15 | 1.98 | 0.0196 | 0.89 |
| GOTERM_BP_FAT | GO:0043450~alkene biosynthetic process | 4 | 6.80 | 0.0196 | 0.88 |
| GOTERM_BP_FAT | GO:0019370~leukotriene biosynthetic process | 4 | 6.80 | 0.0196 | 0.88 |
| GOTERM_BP_FAT | GO:0006636~unsaturated fatty acid biosynthetic process | 5 | 4.75 | 0.0200 | 0.87 |
| GOTERM_BP_FAT | GO:0048858~cell projection morphogenesis | 15 | 1.98 | 0.0202 | 0.86 |
| GOTERM_BP_FAT | GO:0048747~muscle fiber development | 5 | 4.61 | 0.0221 | 0.87 |
| GOTERM_BP_FAT | GO:0001657~ureteric bud development | 5 | 4.61 | 0.0221 | 0.87 |
| GOTERM_BP_FAT | GO:0016485~protein processing | 9 | 2.59 | 0.0227 | 0.87 |
| GOTERM_BP_FAT | GO:0007166~cell surface receptor linked signal transduction | 73 | 1.27 | 0.0231 | 0.86 |
| GOTERM_BP_FAT | GO:0002253~activation of immune response | 8 | 2.75 | 0.0263 | 0.88 |
| GOTERM_BP_FAT | GO:0031175~neuron projection development | 15 | 1.89 | 0.0281 | 0.89 |
| GOTERM_BP_FAT | GO:0007186~G-protein coupled receptor protein signaling pathway | 47 | 1.35 | 0.0297 | 0.90 |
| GOTERM_BP_FAT | GO:0048666~neuron development | 18 | 1.71 | 0.0347 | 0.92 |
| GOTERM_BP_FAT | GO:0051604~protein maturation | 9 | 2.38 | 0.0354 | 0.92 |
| GOTERM_BP_FAT | GO:0045596~negative regulation of cell differentiation | 13 | 1.94 | 0.0362 | 0.92 |
| GOTERM_BP_FAT | GO:0051960~regulation of nervous system development | 12 | 2.02 | 0.0363 | 0.91 |
| GOTERM_BP_FAT | GO:0001656~metanephros development | 5 | 3.67 | 0.0462 | 0.95 |
| GOTERM_BP_FAT | GO:0050768~negative regulation of neurogenesis | 5 | 3.59 | 0.0495 | 0.96 |
