# Supplementary material for: Preliminary evidence for association of genetic variants in pri-miR-34b/c and abnormal miR-34c expression with attention deficit and hyperactivity disorder
Source: Transl Psychiatry. 2016 Aug 30;6(8):e879–. doi: 10.1038/tp.2016.151 (PMC5022091; doi:10.1038/tp.2016.151)
Supplement: Supplementary Table 9 [file tp2016151x10.doc]

**Supplementary Table 9** Significant GO terms and clusters considering the gene set identified in the *trans*-eQTL analysis in 45 adults with ADHD (681 transcripts; enrichment score>2 and P-value<0.05).

| **Cluster 1** | **Enrichment Score: 1.60** | | | | |
| --- | --- | --- | --- | --- | --- |
| **Category** | **Term** | **Count** | **Fold Enrichment** | **P-value** | **B-H P-value** |
| GOTERM_BP_FAT | GO:0007411~axon guidance | 10 | 3.017 | 5.9e-03 | 0.872 |
| GOTERM_BP_FAT | GO:0007409~axonogenesis | 14 | 2.342 | 7.0e-03 | 0.879 |
| GOTERM_BP_FAT | GO:0048667~cell morphogenesis involved in neuron differentiation | 14 | 2.163 | 0.013 | 0.920 |
| GOTERM_BP_FAT | GO:0032990~cell part morphogenesis | 16 | 2.018 | 0.013 | 0.907 |
| GOTERM_BP_FAT | GO:0048812~neuron projection morphogenesis | 14 | 2.122 | 0.015 | 0.865 |
| GOTERM_BP_FAT | GO:0030182~neuron differentiation | 23 | 1.695 | 0.018 | 0.887 |
| GOTERM_BP_FAT | GO:0000904~cell morphogenesis involved in differentiation | 15 | 1.985 | 0.020 | 0.887 |
| GOTERM_BP_FAT | GO:0048858~cell projection morphogenesis | 15 | 1.977 | 0.020 | 0.857 |
| GOTERM_BP_FAT | GO:0031175~neuron projection development | 15 | 1.892 | 0.028 | 0.891 |
| GOTERM_BP_FAT | GO:0048666~neuron development | 18 | 1.714 | 0.035 | 0.922 |
| GOTERM_BP_FAT | GO:0006928~cell motion | 22 | 1.495 | 0.063 | 0.965 |
| GOTERM_BP_FAT | GO:0032989~cellular component morphogenesis | 19 | 1.545 | 0.067 | 0.963 |
| GOTERM_BP_FAT | GO:0000902~cell morphogenesis | 17 | 1.542 | 0.086 | 0.966 |
| GOTERM_BP_FAT | GO:0030030~cell projection organization | 16 | 1.404 | 0.168 | 0.986 |
| **Cluster 2** | **Enrichment Score: 1.55** | | | | |
| **Category** | **Term** | **Count** | **Fold Enrichment** | **P value** | **B-H P-value** |
| GOTERM_BP_FAT | GO:0006690~icosanoid metabolic process | 7 | 4.809 | 3.1e-03 | 0.998 |
| GOTERM_BP_FAT | GO:0006691~leukotriene metabolic process | 5 | 7.338 | 4.2e-03 | 0.948 |
| GOTERM_BP_FAT | GO:0033559~unsaturated fatty acid metabolic process | 7 | 4.431 | 4.7e-03 | 0.914 |
| GOTERM_BP_FAT | GO:0043449~cellular alkene metabolic process | 5 | 7.019 | 5.0e-03 | 0.878 |
| GOTERM_BP_FAT | GO:0046456~icosanoid biosynthetic process | 5 | 5.207 | 0.015 | 0.890 |
| GOTERM_BP_FAT | GO:0019370~leukotriene biosynthetic process | 4 | 6.797 | 0.020 | 0.875 |
| GOTERM_BP_FAT | GO:0043450~alkene biosynthetic process | 4 | 6.797 | 0.020 | 0.875 |
| GOTERM_BP_FAT | GO:0006636~unsaturated fatty acid biosynthetic process | 5 | 4.748 | 0.020 | 0.868 |
| GOTERM_BP_FAT | GO:0006631~fatty acid metabolic process | 11 | 1.794 | 0.087 | 0.963 |
| GOTERM_BP_FAT | GO:0006633~fatty acid biosynthetic process | 6 | 2.452 | 0.097 | 0.969 |
| GOTERM_BP_FAT | GO:0046394~carboxylic acid biosynthetic process | 8 | 1.666 | 0.204 | 0.988 |
| GOTERM_BP_FAT | GO:0016053~organic acid biosynthetic process | 8 | 1.666 | 0.204 | 0.988 |
| GOTERM_BP_FAT | GO:0008610~lipid biosynthetic process | 10 | 1.000 | 0.671 | 0.999 |
