# Supplementary material for: Preliminary evidence for association of genetic variants in pri-miR-34b/c and abnormal miR-34c expression with attention deficit and hyperactivity disorder
Source: Transl Psychiatry. 2016 Aug 30;6(8):e879–. doi: 10.1038/tp.2016.151 (PMC5022091; doi:10.1038/tp.2016.151)
Supplement: Supplementary Table 10 [file tp2016151x11.doc]

**Supplementary Table 10** Significant Ingenuity Pathway networks (score>3) and top associated functions and diseases.

| **ID** | **Molecules in Network** | **Score** | **Focus Molecules** | **Top Diseases and Functions** |
| --- | --- | --- | --- | --- |
| 1 | ACP2, ACTA1, ALDH3A2, BDNF, BMPR1A, CD59, Cg, COL12A1, CRP, DLX5, ERK, ERK1/2, estrogen receptor, F2, Focal adhesion kinase, FSH, GAREM, GNRH1, GPR183, GPRC5A, ITGA3, KIDINS220, Lh, MMP7, NMUR2, NOTCH3, OSTF1, RAPGEF3, RGS4, SMAD5, SULT2A1, TAP2, TFF3, TNK2, ZDHHC2 | 37 | 28 | Cell Signaling, Small Molecule Biochemistry, Vitamin and Mineral Metabolism |
| 2 | ADAMTS9, BCR (complex), CFB, CLDN1, COPS5, DGKZ, EIF4A3, FMR1, GDNF, IFI44L, IFN Beta, IFNB1, IRAK2, Jnk, KHSRP, KSR2, LTA, MFN1, mir-29, MX1, NFkB (complex), PI3K (complex), PRKAA, PTPN11, Ras, RSAD2, SENP2, TCR, TLR3, TNFRSF14, TRIM9, TRIM38, UBE2N, WTAP, XIAP | 35 | 27 | Cell-To-Cell Signaling and Interaction, Hair and Skin Development and Function, Embryonic Development |
| 3 | ABCC1, Actin, Akt, BRD4, DCTPP1, DGKD, EPHB2, ERCC8, FBXL19, GPSM3, HBE1, Histone h3, Histone h4, IgG, KDM5B, KLHDC10, KLK6, LGALS3BP, MED4, MED6, MED20, MED22, MED26, Myosin, NR2F2, P38 MAPK, PLS3, PSD3, RELN, RNA polymerase II, SORBS3, SORCS3, TES, TUBB2A, UBB | 25 | 22 | Gene Expression, Infectious Diseases, Cancer |
| 4 | AGRN, BAG5, CCDC77, CROT, DHTKD1, DPYSL5, ELMOD1, LRRC16A, MGME1, NUPR1, PEX5, PEX12, PPFIBP2, PXDC1, RAB29, RNU11, SLC2A12, SYTL2 | 20 | 15 | Developmental Disorder, Hereditary Disorder, Metabolic Disease |
| 5 | ALOX12B, CCNT1, CPNE6, CXCL8, GRB2, HIST3H2A, INTS7, IRS4, L3MBTL3, LPA, MBD2, MBD3, MED28, NCOA3, NFE2L2, NISCH, PAK2, PIP5K1A, POLR2A, PPP2R1A, PSMA3, PTOV1, Rnr, RPS4X, SSRP1, STAU1, SUPT16H, TIRAP, TUBA1B, XIAP, XRCC6, ZMYND8, ZNF592, ZNF609, ZNF687 | 13 | 14 | Embryonic Development, Carbohydrate Metabolism, Lipid Metabolism |
| 6 | ABCA1, ACTL6A, ADNP, AREG, ARHGAP4, ARID1A, ARID1B, BANF1, CXCR6, DLX2, ENTPD3, ETS2, FABP4, GSG1, HS3ST3B1, IL23A, INHBA, INPP1, KCNJ2, LAMP3, MAGEB2, MAGT1, OXCT1, PLCG1, PTP4A1, SERPINB2, SMARCA2, SMARCA4, SMARCB1, SMARCC1, SMARCC2, SMARCE1, SNTA1, TNFSF14, TREM1 | 13 | 14 | Cellular Compromise, Cell Cycle, DNA Replication, Recombination, and Repair |
| 7 | ACSL3, BLM, CDC6, CDC7, CDC25C, CXXC1, DBP, DHODH, E2f, GDF15, GPR87, MCM2, MDM4, MEG3, mir-194, NASP, NDUFA4L2, NQO1, NUMB, P-TEFb, PGM3, PHB, POLH, RAD51, RBBP5, RCHY1, SETD8, SETD1A, TOP1, TP53, TTK, USP39, WRN, WRNIP1, XRCC6 | 13 | 14 | DNA Replication, Recombination, and Repair, Cell Cycle, Embryonic Development |
| 8 | AKAP13, BCL10, BRAF, COMMD1, COMMD6, COMMD7, COPS5, DIABLO, FADD, FHDC1, Ggt, GLDN, GPR101, GSTM1, GSTM2, GSTT1, IRF1, KCNN2, KPNA1, LCMT1, LTB, MAP3K14, MRPL39, NR0B1, NR3C1, PDLIM2, PPP2CA, PPP2R1B, RELA, STAT5A, TRAF5, TSC22D3, TSPYL5, XIAP, ZNF300 | 13 | 14 | Lymphoid Tissue Structure and Development, Organ Morphology, Gene Expression |
| 9 | AGO1, AGO2, BTBD7, BTG2, CDC20, CDH6, CEBPA, CYP2B6, EP300, ERN1, GATA2, JUN, JUND, KLF5, let-7a-5p (and other miRNAs w/seed GAGGUAG), LIN28A, mir-17, mir-23, mir-30, mir-31, mir-134, mir-208, mir-219, mir-342, MMP9, MYOG, OAS2, PTX3, SERPINI1, SFTPD, TARBP2, TMPRSS2, WNT10A, WWOX, XPO5 | 13 | 14 | Cancer, Organismal Injury and Abnormalities, Respiratory Disease |
| 10 | AFTPH, AKR1C1/AKR1C2, BMP4, CD44, CLDN7, CTGF, DDAH1, DHX57, EGR1, EPCAM, Focal adhesion kinase, GAP43, ID1, ISL1, MET, mir-21, MITF, MMP7, NANOG, PCCA, PDX1, PLA2G1B, POU5F1, PRKCE, PTBP3, SBDS, SNAI2, SOX2, SPP1, SRRM1, TCF, TSPAN8, UPF2, XIAP, ZFP42 | 13 | 14 | Cellular Movement, Tissue Development, Cancer |
| 11 | ANXA7, APEX1, ATG12, BHMT2, C9orf3, DCLK1, DNER, EXOG, FIGLA, FRG1, HNRNPA2B1, HNRNPK, HNRNPU, HYAL1, ID2, IFNAR1, LMO2, mir-8, mir-96, mir-223, MKNK1, MYC, NFYA, PMS2, PTBP1, SRSF1, TAL1, TCF3, TWIST1, WT1, WTAP, XRCC6, YBX1, ZNF224, ZP2 | 11 | 13 | Cancer, Hematological Disease, Immunological Disease |
| 12 | ANAPC2, BTG2, CCND1, CDC16, CDC20, CDC27, CELF1, CRLF1, DNMT1, EPCAM, ESR1, FZR1, KCNIP3, KDELR2, KIAA1524, LMNA, LTB, N-cor, NFYA, NFYB, PDYN, PTTG1, RASSF1, RB1, RBCK1, RNF31, S100A16, TBCK, TMEM74, TOP2A, UBE2D3, UBE2N, VAMP2, VPS36, ZNF280C | 11 | 13 | Cell Cycle, Cellular Development, Cancer |
| 13 | ADAM15, ADH5, BACE1, CBS/LOC102724560, CD59, CTSC, DGKQ, IDO1, IFFO1, IFNGR1, IL13, IRAK3, LIPA, LONP1, LPL, LTB, MEOX1, MT-CO2, MT-CYB, NCLN, NHLH1, NID1, NR0B2, Nr1h, NR1H3, PAPSS1, PDCD1, PPP2R1B, REST, SCTR, SP1, SREBF1, SRRM4, TNF, TUB | 11 | 13 | Immunological Disease, Organismal Survival, Hematological System Development and Function |
| 14 | ADCY1, BCL2L1, BCL2L2, CCR5, CDX2, CFTR, CGA, CGB (includes others), EGF, Erm, FADS1, FPR2, GAB2, GCG, HAVCR2, HOXA3, IFNG, IGFBP1, IL23, IL32, INS, KLRC1, LHCGR, mir-15, MTNR1B, OAS3, PIK3CG, PTPRT, SELE, SMC1B, TCF7L2, TNFSF13B, UBXN4, USP33, VCP | 10 | 12 | Cell Signaling, Nucleic Acid Metabolism, Small Molecule Biochemistry |
| 15 | ASF1A, BTRC, C17orf85, CCNA2, CDK1, CDK2, CDKN1B, CDKN2A, CUL1, DDX39A, DNMT1, DNMT3B, ERG, ERH, ESRP2, FBXO11, GAPVD1, LONRF1, MID1, MYO1D, PSMD13, PTTG1, RASSF1, RBMS1, SARNP, SKP1, SKP2, SLIT2, SNAI1, SUZ12, THOC2, TRIM52, WNT1, YOD1, YWHAG | 10 | 12 | Cell Cycle, Respiratory System Development and Function, Connective Tissue Development and Function |
| 16 | APOB, ARNT, BARD1, BRAT1, BRCA1, BRCA2, BRIP1, CHEK2, CYP3A5, EXO1, FOSL2, G6PC, Growth hormone, GTF2IRD1, IFI16, KAT5, KPNA2, KRT35, LDL-cholesterol, LDLR, LPL, MAPK9, MED21, MICALL2, mir-95, MLH1, MSH6, PCBP3, RHO, RUVBL2, SHFM1, SMC1A, ST5, WDR62, WIZ | 10 | 12 | DNA Replication, Recombination, and Repair, Cancer, Hereditary Disorder |
| 17 | APOA1, APOC3, CDC7, ETS2, hemoglobin, HP, KBTBD6, KLHL9, MIF, miR-542-3p (miRNAs w/seed GUGACAG), NUMB, NUP153, PHB, PRKAA, PRKAA2, PTTG1, RAE1, RPL22, SIAH1, SNCAIP, SPATS2, SPIN2A/SPIN2B, TERC, TERT, THRB, TOP3A, TP53, UBE3A, XPNPEP2, XPO1, XRCC6, ZKSCAN3, ZNF18, ZNF24, ZNF552 | 10 | 12 | Cellular Assembly and Organization, Cellular Function and Maintenance, DNA Replication, Recombination, and Repair |
| 18 | 26s Proteasome, ABCA1, APP, AR, ARL3, BACE1, CUL3, DAXX, DHCR24, EIF4A1, EIF4G1, ENTPD7, FGF10, GNB2L1, IREB2, KIAA0368, LARP4B, LMAN1, LMNB1, MDM4, MIF, PABPC1, PAIP1, PAX3, PDE6D, RAB28, SFPQ, SH3BP4, SMARCE1, SMPD2, SPOP, TCF7L2, TOP1, YWHAG, YWHAZ | 9 | 11 | Protein Synthesis, Nervous System Development and Function, Tissue Morphology |
| 19 | ALKBH8, BST2, CCDC82, CCNC, CCT2, CCT3, CCT4, CCT5, CCT7, CCT8, CCT6A, CDK19, CYP1A1, DAB2, EHF, F9, GCN1L1, IFIH1, IL1B, ITGAM, ITPA, KLKB1, KNG1, LHX8, MAPK1, MED12, MED13, MYOC, SERPINA3, SETMAR, SOX11, SPRR1B, TCP1, THY1, TRMT112 | 9 | 11 | Cellular Assembly and Organization, Cell-To-Cell Signaling and Interaction, Reproductive System Development and Function |
| 20 | ABCC5, ABI1, ADGRF1, AKAP12, ALDOC, CAV1, CD9, CDC42EP5, CDH2, CTGF, EGF, EGFR, EPAS1, EPS8L3, ESR1, ESRRA, ETS1, FAM13A, FOS, GYS2, HAMP, HIF1A, KDR, KLF16, NDUFAF5, PRKCA, PTGS2, RASAL1, SCAMP1, SERPINE1, SOX9, SP1, TMPRSS6, TRIP4, VEGFA | 7 | 10 | Cellular Movement, Cardiovascular System Development and Function, Cancer |
