# Supplementary material for: Preliminary evidence for association of genetic variants in pri-miR-34b/c and abnormal miR-34c expression with attention deficit and hyperactivity disorder
Source: Transl Psychiatry. 2016 Aug 30;6(8):e879–. doi: 10.1038/tp.2016.151 (PMC5022091; doi:10.1038/tp.2016.151)

Supplementary Figure 1 Graphical representation of the chromosomal region containing the miR-34b/c cluster and the 3' Untranslated Regions (3'UTRs) of the *MET*, *NOTCH2* and *HMGA2* genes, including the SNPs selected for the association study and the putative miRNA binding sites. (*) In bold, SNPs associated with ADHD.

MIR34B* and *MIR34C

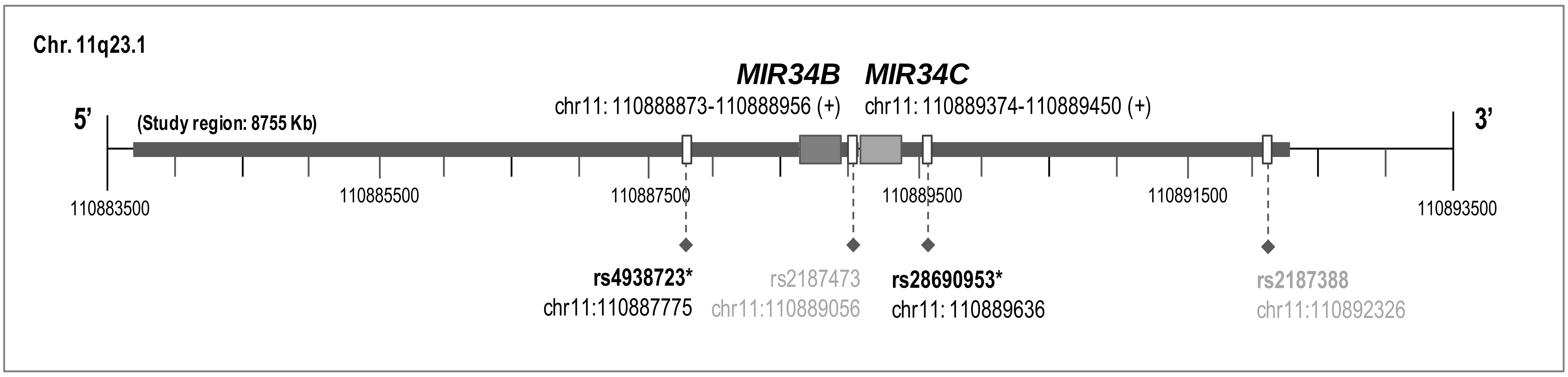

MET

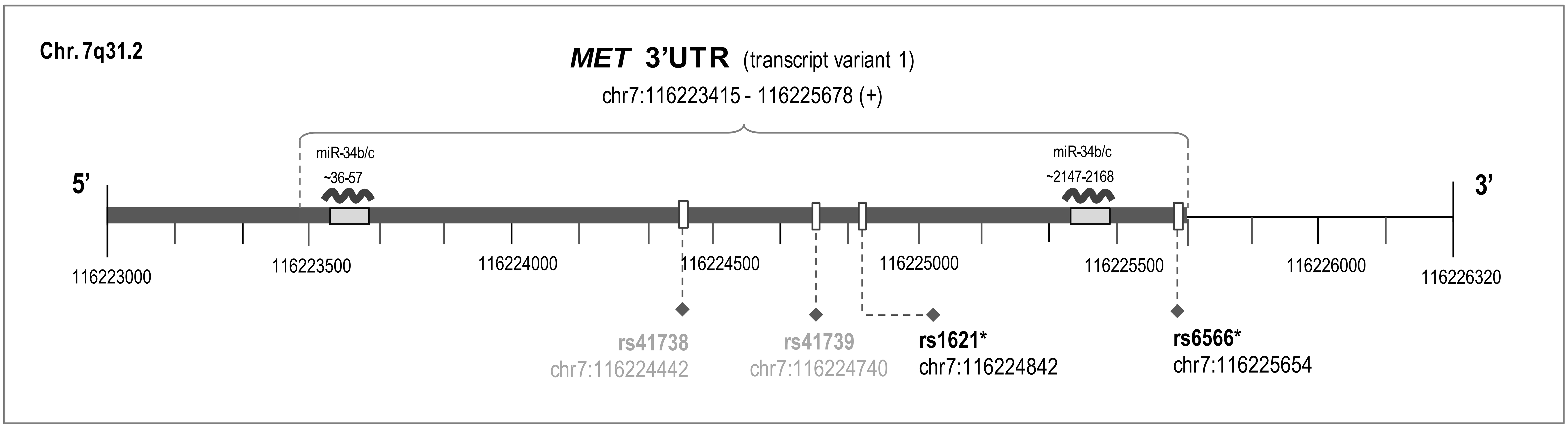

NOTCH2

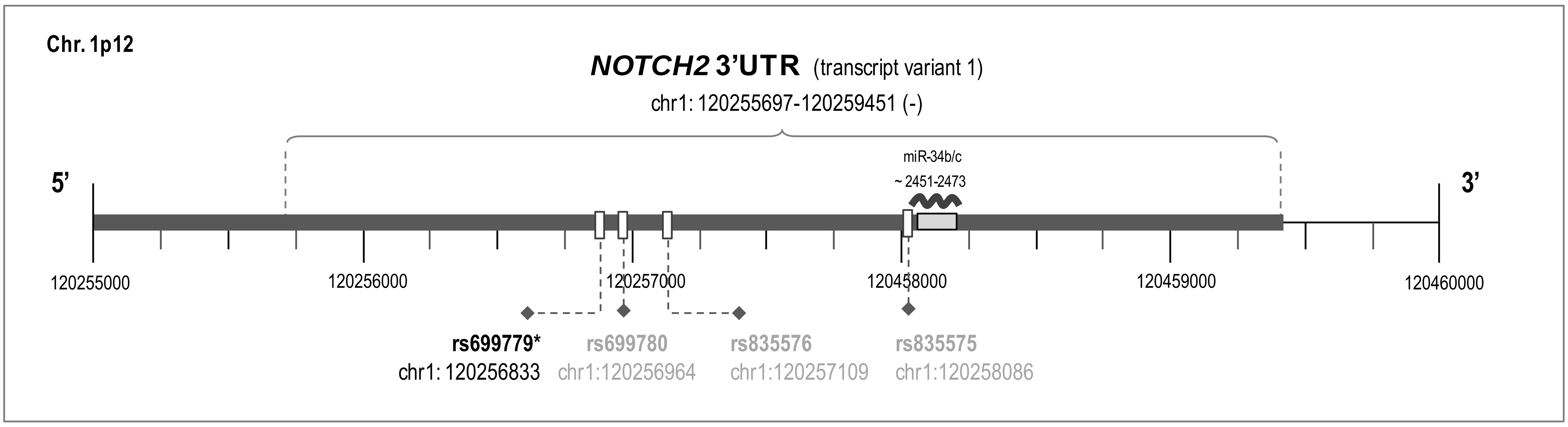

HMGA2

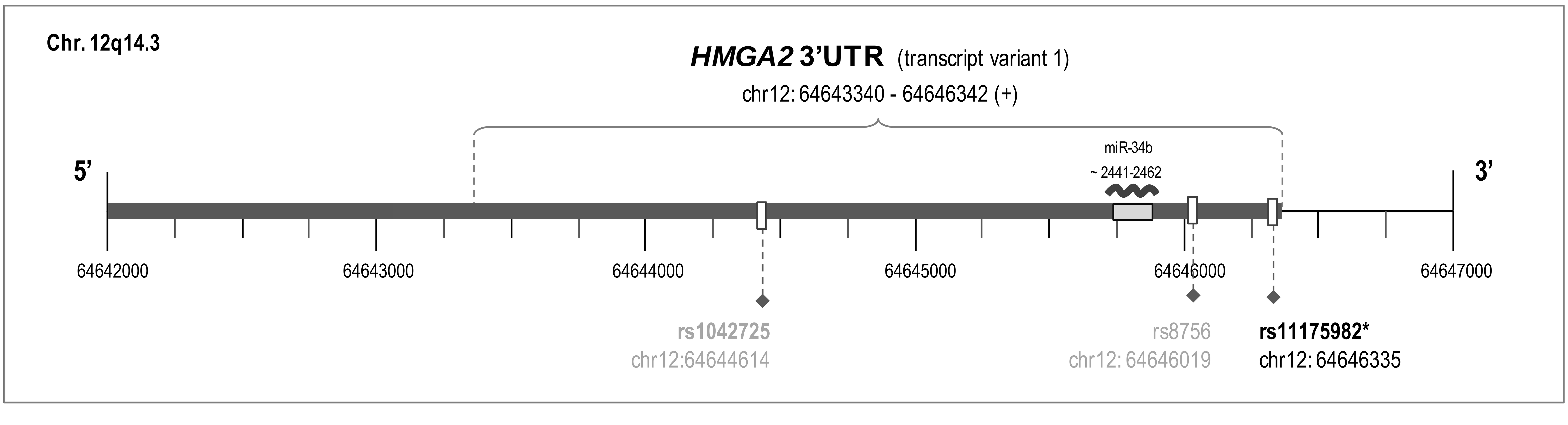

Supplement: Supplementary Figure 1 [file tp2016151x12.pdf]
